# A systematic review and network meta-analysis of interventions to preserve insulin-secreting beta cell function in people newly diagnosed with type 1 diabetes: results from randomised controlled trials of immunomodulatory therapies

**DOI:** 10.1186/s12916-025-04201-z

**Published:** 2025-07-01

**Authors:** Sophie E. Beese, Malcolm J. Price, Claire Tomlinson, Pawana Sharma, Isobel M. Harris, Ada Adriano, Lauren M. Quinn, Ritu Gada, Thomas J. Horgan, Fiona Maggs, Martin Burrows, Krishnarajah Nirantharakumar, G. Neil Thomas, Robert C. Andrews, David J. Moore, Parth Narendran

**Affiliations:** 1 Department of Applied Health Sciences, School of Health Sciences, University of Birmingham, Birmingham, B15 2TT UK; 2 Department of Public Health, Canadian University Dubai, Dubai, United Arab Emirates; 3 Department of Immunology and Immunotherapy, University of Birmingham, and University Hospitals Birmingham NHS Foundation Trust, Birmingham, UK; 4 Department of Oral and Maxillofacial Surgery, Shrewsbury and Telford Hospital NHS Trust, Telford, UK; 5 University of Exeter Medical School, Exeter, UK

**Keywords:** Systematic review, Network meta-analysis, Type 1 diabetes, Beta cells

## Abstract

**Background:**

Type 1 diabetes is characterised by the immune-mediated destruction of pancreatic beta cells. We aimed to determine the effectiveness of immunotherapies for preserving residual beta cell function in newly diagnosed (stage 3) type 1 diabetes.

**Methods:**

Searches were carried out in MEDLINE, Embase, Cochrane CENTRAL and trial registries until 31st Jul 2024. RCTs of immunotherapies to preserve beta cells in newly diagnosed type 1 diabetes were included. Data were extracted using a bespoke, piloted extraction sheet. Risk of bias was assessed using Cochrane Risk of Bias Tool 1. A random effects network meta-analysis was undertaken in R. The primary outcome was C-peptide. Interventions were analysed by class.

**Results:**

Sixty trials were included (4597 patients, 32 intervention classes). Forty-one trials of 42 interventions were eligible for network meta-analysis. Eleven interventions demonstrated statistically significantly higher levels of C-peptide than placebo at 12 months, mesenchymal stem cells (autologous and Wharton’s jelly-derived cells), azathioprine, interferon-alpha (5000 IU), autologous dendritic cells, anti-TNF golimumab, low-dose ATG, 3 mg 1-course anti-CD3 teplizumab, baricitinib, cyclosporin and 9/11 mg 2-course anti-CD3 teplizumab but with substantial heterogeneity present (*I*^2^ = 66%). Azathioprine ranked highest (median ranking 3rd); however, rankings demonstrated relatively wide confidence intervals and thus uncertainty in exact rank order of near adjacent therapies. Risk of bias assessment identified poor reporting, particularly in older trials, but few studies demonstrated high risk overall.

**Conclusions:**

Eleven of 42 interventions demonstrated statistically significantly higher C-peptide levels than placebo at 12 months in the network meta-analysis. These results have identified the 11 most promising therapies trialled and help to direct future head-to-head clinical trials to support approvals for interventions to treat those newly diagnosed with type 1 diabetes. However, data for some interventions originated from small studies (mesenchymal stem cell therapies, azathioprine, autologous dendritic cells) and findings should be considered as hypothesis generating and interpreted with caution due to evidence heterogeneity.

**Systematic review registration:**

The protocol for the systematic review was registered on PROSPERO, the international database of prospectively registered systematic reviews (registration: CRD42018107904).

**Supplementary Information:**

The online version contains supplementary material available at 10.1186/s12916-025-04201-z.

## Background

Type 1 diabetes is a chronic autoimmune condition resulting from immune-mediated destruction of pancreatic insulin-secreting beta cells and insulin insufficiency [1]. A significant number of functioning beta cells remain at diagnosis and persistence of these cells is associated with better glucose control and fewer microvascular complications [2]. Therefore, therapies that preserve beta cell function could have real clinical benefit.

Clinical trials over the last four decades have tested many therapies with the aim of slowing the loss of beta cells in people newly diagnosed with type 1 diabetes [3]. The efficacy of these therapies has not previously been formally synthesised and reviewed in a comprehensive manner. A network meta-analysis is important to understand the relative effects of the therapies. This paper reports the effectiveness of interventions which modulate the immune system, specifically those that do so in a broad way and do not specifically target pancreatic autoimmunity.

The rationale for immunomodulatory therapy for beta cell preservation is based on immune cells playing a central role in the aetiology of type 1 diabetes, both through failure in immune regulation and in orchestrating inflammatory beta cell death [4]. The rationale for exploring immunomodulatory therapies also comes from evidence of clinical benefit of such therapies which are now licenced for routine use in other autoimmune diseases [5].

Several systematic reviews of the efficacy of immunomodulatory agents for preservation of beta cell function in newly diagnosed type 1 diabetes have previously been undertaken. Gandhi (2008) included a review of only a small number of potential immunomodulatory agents and Felton (2023) employed a limited search strategy, focusing on features potentially linked to treatment response, with little information about overall effectiveness of, or comparison between therapies [6, 7]. More recently, a cross-trial efficacy comparison of seven agents for beta cell preservation trialled in the last 10 years has been reported (Jacobsen 2020), as well as a systematic review and meta-analysis of immunotherapies (Lin 2024) [8, 9]. Through analysis of co-variance modelling of the different outcome measures, two different agents, anti-thymocyte globulin (ATG) and anti-CD3 T-cell agent anti-CD3 teplizumab, were shown to demonstrate most efficacy by Jacobsen. However, this study did not include older trials and restricted their analysis to the chosen agents. Conversely, Lin combined antigen-specific and non-antigen-specific immunotherapies together respectively and demonstrated that collectively non-antigen-specific immunotherapies significantly improved C-peptide, rather than which therapies were relatively most effective. Not all relevant trials and interventions were included. This paper aims to overcome these limitations.

A systematic review and network meta-analysis of randomised controlled trials of immunomodulatory therapies in patients newly diagnosed with type 1 diabetes that report C-peptide was undertaken with a view to comparing the relative effectiveness of these agents.

## Methods

### Search strategy and selection criteria

The systematic review methods were guided by current best practice. The protocol for the systematic review was registered on PROSPERO, the international database of prospectively registered systematic reviews (registration: CRD42018107904) and the review is reported in accordance with the Preferred Reporting Items for Systematic Reviews involving Network Meta-Analyses statement [10].

Bibliographic database searches of MEDLINE, Embase, Cochrane CENTRAL, Clinical Trials.gov and WHO International Clinical Trials Registry were undertaken, supplemented by citation checking. Bibliographic databases were searched using a combination of index and free text terms (search strategies available in Additional file 1: Search strategies). A study design filter with maximised sensitivity for randomised controlled trials (RCTs) was used in MEDLINE and Embase (via Ovid) [11]. Databases were searched from inception to 31 st July 2024. Searches were not restricted by language or publication date. Study authors were contacted where necessary for further information.

RCTs were eligible if they included adults or children diagnosed with type 1 diabetes within the previous 3 years (or population mean duration of diabetes ≤ 3 years) randomised to any immunomodulatory therapies or any comparator in any setting with at least 1 month follow-up. The primary outcome was defined as beta cell function through any measurement of C-peptide. Secondary outcomes were insulin doses, glucose control measured by HbA1c and adverse events. Journal articles, conference abstracts and trial registration records were eligible.

Endnote 20 (X9 Clarivate Analytics) was used to facilitate deduplication automatically and manually [12]. Titles and abstracts were screened for relevance using Rayyan [13]. Full text copies of relevant articles were assessed against inclusion criteria. Studies meeting all selection criteria were included. Reasons for exclusion of articles were recorded. Study selection was conducted by two reviewers independently with disagreements resolved by discussion or a third reviewer. Screening and selection were undertaken by SEB, CT, PS, IMH, AA, LMQ, RG, TJH, DJM, PN, FM and MB.

### Data analysis and risk of bias

#### Data extraction

Data were extracted using a bespoke and piloted sheet in MS Excel by a single reviewer with 10% checked by a second reviewer, with additional checking during analysis (see Additional file 1: Further details on data extraction and data synthesis, for more details). Authors were contacted regarding any missing or unclear data, and this was included where acquired. Where missing data were not acquired, these were computed where possible. No imputations were undertaken.

Risk of bias in included studies was assessed using the Cochrane Risk of Bias tool (version 1).

#### Data synthesis and analysis

Included studies were grouped by intervention class. Within each grouping, data for each relevant outcome and the method of assessment were considered. Where reported, outcomes were assessed at 6, 9, 12, 18, 24, 36, 48 and 60 months after initiation of intervention. Studies were categorised by population age; children < 12 (C), adolescents 12–18 (AD) or adults > 18 years (A), or a mixed group of these (MI). Details of pairwise meta-analysis methods can be found in the Additional file 1: Further details on data extraction and data synthesis.

#### Network meta-analysis

Suitability of a network meta-analysis was considered based on assessment of clinical heterogeneity by comparing study population characteristics across trials. The transitivity assumption was evaluated by comparing key clinical and methodological characteristics of studies.

Analyses were carried out in R (R version 4.3.3) [14]. A frequentist approach was taken using the R package “netmeta”, fit using the graph theoretical model [15, 16].

The primary outcome was difference in C-peptide (any meal, glucagon or glucose stimulated measure; fasting, peak or AUC levels) at 12 months follow-up reported as standardised mean difference (SMD). Reports of urinary C-peptide were not included due to contrasts in measurement methodology. Correlations between different measures of C-peptide were assessed and deemed similar enough to be combined (Additional file 1: Table 1 [17–21]). Where studies reported multiple methods of C-peptide measurement, meal-stimulated 2-h AUC was prioritised. Analysis at 24 months follow-up was considered, but there was insufficient trial data to carry this out. Where doses or frequency of delivery of the same interventions differed, those strategies that were most similar (when considering mean cumulative dose by body surface or weight, and decided with speciality clinical input) were combined within the same node (e.g. Anti-CD3 teplizumab, ATG). Analysis was performed by drug class.

Secondary outcomes were HbA1c (%) and insulin dose (units/kg/day) presented using mean differences. Adverse events were summarised and reported but were not suitable for combination in NMA due to differences in the expected types of events elicited from different classes of interventions.

A random-effects model was utilised due to underlying differences in the studies included. Appropriate pooled effect estimates with 95% confidence intervals were reported for each contrast. Correlation effects in multi-arm studies were accounted for by re-weighting all comparisons of each multi-arm study (as per the “netmeta” package) [22]. Placebo or no treatment were the reference standard.

Overall heterogeneity was assessed using *I*^2^ and tau^2^statistic [23]. Total network inconsistency was assessed using the design-by-treatment interaction model [24]. Where significant heterogeneity or inconsistency was found, this was explored, and subgroup and sensitivity analyses were carried out where sufficient studies were available. Sensitivity analyses were considered based on the key effect modifiers of baseline C-peptide, age and risk of bias.

It was planned that small study effects would be assessed using a comparison-adjusted funnel plot. However, as many of our included comparisons had only one trial contributing data, the funnel plots did not provide meaningful results as the pooled effect for each comparison is used as the reference, resulting in the effect being centred on 1 for all comparisons consisting of only one trial.

The main analysis included all available C-peptide data at 12 months. Some trials reported both 2- and 4-h AUC. In this case, the 2-h data were used in the primary analysis (and throughout the sensitivity analyses).

Several sensitivity analyses were also planned and undertaken as follows:

- Restriction to analysis of trials from the main analysis that were assessed as low risk of bias (allocation concealment, double-blinding and loss to follow-up < 20%).
- Analysis of only those trials without imbalances of > 20% in baseline C-peptide.
- Analysis of trials that report change from baseline data only.
- Analysis of trials that report endpoint data only.
- Repeated main analysis of all C-peptide data but using 4-h AUC data where both 2- and 4-h data are reported in a trial.
- Analysis of trials reporting 2- and 4-h AUC data only with 2-h data used where both 2- and 4-h are reported.

Analysis of secondary outcomes included all trials that contributed to the main C-peptide NMA if they also reported insulin dose and/or HbA1c. A sensitivity analysis of trials that were assessed as low risk of bias for each secondary outcome was also undertaken.

Details of assessment of potential effect modifiers are reported in Additional file 1: Further details on data extraction and data synthesis.

Forest plots were used to display results. A drug class hierarchy was produced using the frequentist *P*-value approach as well as the Surface Under the Cumulative Ranking Curve (SUCRA) statistic. The *P*-value is a frequentist approach to ranking treatments and is based on the point estimates and standard errors of the NMA estimates under normality assumption. The *P*-values measure the degree of certainty that a treatment is better than those it is being compared against [25]. The probability of each intervention being at each rank was also calculated, and median rankings with 95% confidence intervals were derived. These analyses were presented in tables and ranking curves.

## Results

**Fig. 1:**
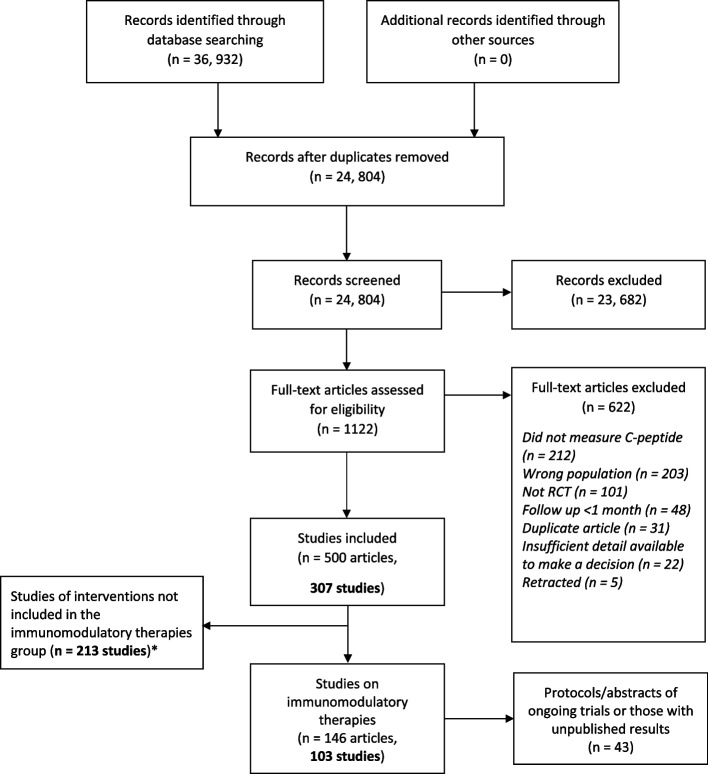
PRISMA flow diagram of studies included in the systematic review and the immunomodulatory group from both the original and updated searches. *The study selection process was utilised for all trials of therapies in T1D across the series of reviews. Some studies compared immunomodulatory therapies with other T1D interventions, and these studies are represented more than once in the flow diagram. Results of the immunomodulatory therapies only are reported here

There were 36,932 records identified from the searches (Fig. 1 PRISMA flowchart). Following removal of duplicates, screening and selection, 307 studies (reported in 500 articles) were included in the wider review on all therapies. Of these, 60 published studies (106 articles) were related to immunomodulatory interventions. There were also 43 ongoing or unpublished studies related to immunomodulatory interventions (Additional file 1: Table 2).

Included studies were categorised into 32 classes by intervention type (53 different interventions). Study characteristics are described in Table 1. Some studies with multiple arms (*n* = 11) or combination therapies (*n* = 7) were present in multiple classes. There were 4597 participants in total. Sample sizes varied from 10 to 516 patients. The mean age of participants ranged from 7.9 to 34.9 years. All but two trials reported inclusion criteria of duration of type 1 diabetes of no more than 12 months.

**Table 1:** Table of characteristics of studies included in the systematic review

**Study ID (No. articles, Age category)** *Inclusion criteria for time since diagnosis and mean/median duration (where reported)*	**Sample Size at randomisation** **(Intervention; comparator)**	**Mean duration of disease (SD)**	**Mean age at baseline (SD)**	**Baseline C-pep (nmol/L)**	**Intervention (Dose; frequency)**	**Comparator (Dose; Frequency)**	**Intervention/Comparator Duration**	**C-Peptide Measurement; Stimulant**	**Timepoints at which C-Peptide measurement reported**	**Time outcome reported (months)**	**Other outcomes of relevance measured**
**Anti-CD3 (Teplizumab)**											
**Herold 2002 ** [26]** (1, MI)** *Within 6 weeks*	24 (12; 12)	NR	Intervention median 13 Control median 16	4H AUC Intervention 0.46 Control 0.56	Teplizumab (14-day course of 1.42 μg per kg of body weight on day 1; 5.67 μg per kg day 2; 11.3 μg per kg day 3; 22.6 μg per kg day 4; 45.4 μg per kg days 5 through 14)	Nothing (placebo not used)	14 days/N/A	Fasting, AUC; Meal stimulated	4H AUC	0, 6, 12	HbA1c; Insulin dose; Adverse events
**Herold 2005 ** [27]** (1, MI)** *Within 6 weeks*	42 (21; 21)	NR	Intervention 13.9 (1.17) Control 14.9 (1.31)	4H AUC Intervention 0.49 Control 0.48	Teplizumab (2 or 4 days of incremental dose escalation to 10 days of a “full dose”. The dose was modified for patients 13–21, approximately 3 mg cumulatively per m^2^)	Nothing (placebo not used)	12–14 days/N/A	AUC; Meal stimulated	0, 15, 30, 60, 120, 150, 180, 210, 240 min, 4H AUC	0, 6, 12, 18, 24	HbA1c; Insulin dose; Adverse events
**Herold 2009 ** [28]** (1,MI)** *Within 6 weeks*	10 (6; 4)	1 month (range 19–54 days)	Intervention 15.5 (1.8) Control 9 (0.9)	4H AUC Intervention 0.88 Control 0.41	Teplizumab (day 1: 460 μg/m^2^, day 2: 919 μg/m^2^, and days 3–12: 1818 μg/m^2^. approximately 3 mg cumulatively per m^2^)	Nothing (placebo not used)	12 days/N/A	AUC; Meal stimulated	0, 15, 30, 60, 90, 120, 180, 210 and 240 min, 4H AUC	0, 12, 24	HbA1c; Insulin dose; Adverse events
**Sherry 2011 ** [29]** (2, MI)** *4 arm trial* *Within 12 weeks*	516 (14-day full dose 209, 14-day low dose 102, 6-day full dose 106; 99)	(weeks) 14-day full dose 8.4 (2.6), 14-day low dose 8.4 (2.6), 6-day full dose 9 (4.5), placebo 8.3 (2.6)	Intervention 14-day full dose 18.9 (7.6) 14-day low dose 17.9 (6.1) 6-day full dose 18.1 (6.9) Control 18.2 (7.3)	4H AUC Intervention 14-day full dose 0.65 14-day low dose NR 6-day full dose NR Control 0.65	Teplizumab (2 groups each with escalating dose daily for 14 days, 1 group escalating dose daily for 6 days plus 8 days of placebo. All doses were repeated at 26 weeks)	Placebo (daily for 14 days, repeated at week 26)	6–14 days × 2/14 days × 2	AUC; Meal stimulated	4H AUC	0, 6, 12, 15, 18, 24	HbA1c; Insulin dose; Hypoglycaemia, Clinical remission, Adverse events
**Herold 2013a ** [30]** (1, MI)** *Between 4 and 12 months*	63 (34; 29)	Intervention 7.09 (2.45) months, control 7.14 (2.44) months	Intervention 12.9 (4.18) Control 12.0 (5.2)	4H AUC Intervention 0.62 Control 0.6	Teplizumab (escalating dose daily up to 826 μg/m^2^ over 14 days.)	Placebo (154 mmol/l NaCl daily for 14 days)	14 days/14 days	AUC; Meal stimulated	4H AUC (11 other timepoints not specified)	0, 6, 12	HbA1c; Insulin dose; Adverse events
**Herold 2013b ** [31]** (2, MI)** *Within 8 weeks*	83 (56; 27)	Intervention 40.4 (8.3) days, control 37.6 (9.0) days	Intervention 12.7 (4.9) Control 12.3 (4.1)	4H AUC Intervention 0.72 Control 0.67	Teplizumab (escalating dose daily with median cumulative dose 11.6 mg over 14 days, course repeated at 12 months)	Standard insulin therapy only	14 days × 2/N/A	AUC; Meal stimulated	4H AUC	0, 12	HbA1c; Insulin dose; Adverse events
**Mathieu 2024 ** [32]** (2, A, AD)** *This study consisted of 2 parts only the second part was randomised* *Within 150 days*	18 (15; 3)	Intervention adults 101 (35.9) days, control 70 (15.6) days; adolescents 122.6 (36.3) days, control 90 (0) days	Intervention adults 27.9 (6.7) adolescents 13.6 (1.1) Control adults 29 (5.7) adolescents 12 (NR, *n* = 1)	2H AUC Intervention adults 0.48 adolescents 0.57 Control (combined) 0.57	Teplizumab (12 days of infusions) and AG019 (a genetically modified Lactococcus lactis secreting human proinsulin and interleukin-10, 6 capsules twice daily)	Placebo and AG019 (Placebo delivered as teplizumab)	Teplizumab (12 days) AG019 (8 weeks)/Placebo as intervention	AUC; Meal stimulated	4H AUC	0, 3, 6, 12	HbA1c; Insulin dose; Adverse events
**Ramos 2023 ** [33]** (1, C, AD)** *Within 6 weeks*	329 (217; 111)	Whole study population 5.3 (0.8) weeks	Intervention 12 (2.5) Control 12.3 (2.6)	4H AUC Intervention 0.51 Control 0.39	Teplizumab (106 μg/m^2^ body surface area on day 1, 425 μg/m^2^ on day 2, and 850 μg/m^2^ on days 3 through 12; total cumulative dose of 9 mg/m^2^ repeated at week 26)	Placebo (Delivered as intervention)	26 weeks/26 weeks	AUC, Meal stimulated	2 and 4H AUC	0, weeks 26, 52, and 78	HbA1c; Insulin dose; Hypoglycaemia; Ketoacidosis; Clinical remission; Quality of Life; Adverse events
**Anti-CD3 (Otelixizumab)**											
**Keymeulen 2005 ** [34]** (4, AD, A)** *Within 4 weeks*	80 (40; 40)	Intervention median 21 (IQR 18–24) days, control 25 (IQR 19–27 days)	Intervention 27 (7) Control 26 (7)	6 min AUC Intervention 0.86 Control 0.94	Otelixizumab (day 1 24 mg then 8 mg for 5 days, this was then reduced to 8 mg on day 1 then 8 mg for 5 days after the first 9 patients due to side effects)	Placebo (delivered as the intervention)	6 days/6 days	C-peptide release during a glucose-clamp, C-peptide as AUC in absence/presence	AUC before glucagon injection (at 60, 90, 120, 140) AUC afterward (140 and 146 min)	0, 6, 12, 18	HbA1c; Insulin dose; Adverse events; Weight;
**Ambery 2014 ** [35]** (2, AD, A)** *Within 90 days*	179 (118; 61)	NR	Intervention 23.6 (8.3) Control 22.5 (8.2)	2H AUC Intervention 0.77 Control 0.7	Otelixizumab (3.1 mg over 8 consecutive days)	Placebo (delivered as the intervention)	8 days/8 days	AUC, Max stimulated; Meal stimulated Urinary c-peptide	2H AUC, Max stimulated (time NR)	0, 6, 12	HbA1c; Insulin dose; Adverse events
**Aronson 2014 ** [36]** (1, AD, A)** *Within 90 days*	272 (181; 91)	NR	Intervention 24.8 (6.6) Control 25.3 (7.2)	2H AUC Intervention 0.77 Control 0.7	Otelixizumab (3.1 mg over 8 consecutive days)	Placebo (delivered as the intervention)	8 days/8 days	AUC, Max stimulated; Meal stimulated	−10, 0, 15, 30, 60, 90, 120 min, 2H AUC	0, 6, 12	HbA1c; Insulin dose; Glucose variability; Hypoglycaemia; Adverse events
**Keymeulen 2021 ** [37]** (2, AD, A)** *4 arm trial* *Within 32 days*	30 (9 mg group 9, 18 mg group 8, 27 mg group 7; 6)	NR	Intervention 9 mg 22.4 (2.13), 18 mg 20.5 (4.31), 27 mg 22.1 (3.98) Control 24.8 (4.4)	2H AUC Intervention (groups combined) 0.7 Control 0.55	Otelixizumab (9, 18 or 27 mg over 6 days)	Placebo (delivered as the intervention)	6 days/6 days	AUC, Meal stimulated	−10, 0, 15, 30, 60, 90 and 120 min, 2H AUC	0, 6, 12, 18, 24	HbA1c; Insulin dose; Glucose variability; Adverse events
**Cyclosporin**											
**Vague 1989 ** [38]** (2, AD, A)** *Within 6 months*	28 (15; 13)	NR	Intervention 26.1 (NR) Control 23.2 (NR)	Fasting Intervention 0.17 Control 0.16 Glucagon stimulated 5 min Intervention 0.31 Control 0.27	Cyclosporin (7.5 mg/kg initial dose; increased up to 10 mg/kg after 3 months if insulin requirements exceeded 50% of initial needs. Discontinued at 6 months if remission achieve, continued until 12 if not)	Placebo (delivered as the intervention)	6–12 months/6–12 months	Fasting; 5 min stimulated, Glucagon stimulated	0, 5 min	0, 12, 24, 36	HbA1c; Insulin dose; Adverse events
**Chase 1990 ** [39]** (2, MI)** *“At diagnosis”*	43 (22; 21)	NR	Intervention 15.3 (6.2) Control 17.0 (8.3)	NR	Cyclosporin (10 mg/kg body weight per day, decreased if necessary)	No intervention (no further details)	4 months/N/A	Max stimulated 6, 12 min; Glucagon stimulated	6, 12 min	4, 12, 36 months	HbA1c; Insulin dose; Clinical remission; Adverse events
**Martin 1991 ** [40]** (4, MI)** *Within 6 weeks*	188 (93; 94)	Intervention 3.9 (2.7) weeks, control 4 (2.6) weeks	Intervention 21.3 (6.4) Control 22.2 (5.1)	Fasting Intervention 0.37 Control 0.29 Glucagon stimulated 6 min Intervention 0.33 Control 0.4	Cyclosporin (initial dose 10 mg/kg daily orally in divided doses at 12-h intervals, adjusted based on serum creatinine levels)	Placebo (delivered as the intervention)	12 months/12 months	Fasting, Max stimulated 6 min; Glucagon stimulated	0, 6 min	0, 6, 9, 12, 18, 24	HbA1c; Glucose variability; Insulin dose; Weight; Clinical remission; Adverse events
**Skyler 1992 ** [41]** (2, MI)** *Within 6 weeks*	25 (12; 13)	NR	Intervention 19 (2.5) Control 20.2 (2.6)	Fasting Intervention 0.37 Control 0.29 2H AUC Intervention 0.68 Control 0.57	Cyclosporin (initial dose 10 mg/kg once daily, adjusted based on serum creatinine levels)	Placebo (olive oil-Labrafil solution delivered daily as intervention)	12 months/12 months	Fasting, Max stimulated, stimulated at various timepoints, AUC; Glucagon stimulated, Meal stimulated, Glucose stimulated	M- before meal, and at 15, 30, 60, 90, 120, 150, 180, 210, and 240 min after the meal, AUC.G- before meal and 6 min after meal GS—0, 1, 3, 5, 10, 20, 30,45, and 60 min after meal	0, 6, 9, 12	HbA1c; Insulin dose; Adverse events
**Pozzilli 1994 ** [42]** (2, MI)** *3 arm trial* *Within 4 weeks*	90 (Cyclosporin and nicotinamide 30, nicotinamide 30; 30)	NR	Intervention 20.1 (9.4) Control 18.5 (8.8)	Fasting Intervention 0.33 Control 0.26 Glucagon stimulated Intervention 0.59 Control 0.43	Cyclosporin and nicotinamide group (Cyclosporin 5 mg/kg of bodyweight daily in divided doses at 12-h intervals, adjusted based on serum creatinine levels)	Insulin therapy only	12 months/N/A	Fasting, Stimulated; Glucagon stimulated	0, time stimulated measured NR	0 only	HbA1c; Insulin dose; Clinical remission; Adverse events
**CD2 (Alefacept)**											
**Rigby 2013 ** [43]** (4, AD, A)** *Within 100 days*	49 (33; 16)	NR	Intervention 20.3 (6.41) Control 19.5 (6.15)	2H AUC Intervention 0.85 Control 0.64	Alefacept (15 mg weekly for 12 weeks, 12 week break then further 12 week treatment)	Placebo (Delivered as intervention)	36 weeks/36 weeks	AUC, 4H peak; Meal stimulated	2H and 4H AUC, no other timepoints reported	0, 6, 12, 24	HbA1c; Insulin dose; Hypoglycaemia; Adverse events
**Anti-CD20 (Rituximab)**											
**Pescovitz 2009 ** [44]** (2, MI)** *Between 3 weeks and 3 months*	87 (57; 30)	Intervention 80 (22) days, control 83 (19) days	Intervention 19 (8.6) Control 17.3 (7.8)	2H AUC Intervention 0.71 Control 0.71	Rituximab (375 mg per square metre of body-surface area given on study days 1, 8, 15, and 22)	Placebo (identical solution without rituximab, delivered as intervention)	22 days/22 days	AUC; Meal stimulated	Intervals of 15 to 30 min for 2 h, AUC	0, 6, 12, 18, 24, 30	HbA1c; Insulin dose; Adverse events
**Zielinski 2022 ** [45]** (2, C, AD)** *(Also included in the T-cell group)* *NR, mean duration 6 months*	25 (Tregs/rituximab 12; Tregs/Placebo 13)	Tregs/Rituximab 6 (4.2) months, Tregs/Placebo 6.5 (4.2) months	Intervention (Tregs/Rituximab) 12.9 (1.2) Control (Tregs/Placebo) 13.3 (1.5)	4H AUC Intervention 0.91 Control 0.836 Fasting Intervention 0.36 Control 0.36	Tregs/Rituximab (4 infusions of rituximab, 375 mg/m^2^ of body surface area on days 14, 22, 29 and 36)	Tregs/Placebo (2 doses of Tresg, 30 × 10^^^6Tregs/kg body weight; each at day 0 and month 3 and placebo delivered as intervention)	36 days/36 days	Fasting, AUC; Meal stimulated	0, 15, 30, 60, 90, 120, 180, 210 and 240 min, AUC	Weeks 2, 5, 12, 26, 39, 52, 65, 78, 92 and 104	HbA1c; Insulin dose; Hypoglycaemia; Ketoacidosis; Clinical remission; Adverse events
**CTLA-4—Abatacept**											
**Orban 2011 ** [46]** (3, MI)** *Within 100 days*	112 (77; 35)	Intervention 87.9 (14.1) days, control 83.2 (17.8) days	Intervention 13.9 (6.9) Control 13.7 (5.3)	2H AUC Intervention 0.74 Control 0.75	Abatacept (10 mg/kg, maximum 1000 mg per dose, given on days 1, 14, and 28, and then every 28 days with the last dose on day 700)	Placebo (saline infusion, delivered as intervention)	700 days/700 days	AUC; Meal stimulated	2H AUC	0, 6, 12, 18,24	HbA1c; Insulin dose; Hypoglycaemia; Adverse events
**Anti-thymocyte globulin (ATG)** *(Haller 2014b and Haller 2018 are also in the GCSF group)*											
**Saudek 2004 ** [47]** (1, A)** *Within 1 month*	17 (11; 6)	Intervention 10.9 (9.46) days, control 8.4 (9.5) days	Intervention 28.8 (5.2) Control 26 (NR)	Fasting Intervention 0.39 Control 0.3	ATG (9 mg/kg first dose, additional doses of 3 mg/kg on further 3 days)	Placebo (Saline delivered as intervention)	4 days/4 days	Fasting, Stimulated; Glucagon stimulated	Post-glucagon stimulated timepoint NR	0, 6, 12	HbA1c; Insulin dose; Clinical remission; Adverse events
**Gitelman 2013 ** [48]** (2, MI)** *Within 100 days*	58 (38, 20)	Intervention 69 (21) days, control 76.5 (18) days	Intervention 19.4 (6.6) Control 20.5 (7.0)	2H AUC Intervention 0.86 Control 0.93	ATG (6.5 mg/kg on day 1, and 2 mg/kg on days 2–4)	Placebo (saline delivered as intervention)	4 days/4 days	AUC; Meal stimulated	2H and 4H AUC	0, 6, 12, 18, 24	HbA1c; Insulin dose; Hypoglycaemia; Ketoacidosis; Adverse events
**Haller 2018 ** [49]** (2, A, AD)** *Also listed in GCSF group. 3-arm trial with ATG/GCSF combination group and ATG alone group* *Within 100 days*	89 (ATG 29, ATG/GCSF 29; 31)	ATG/GCSF median 83 (range 49–97) days, ATG 81 (range 47–100) days, control 84 (range 52–99) days	Intervention ATG (and GCSF placebo) 18.1 (6.9), Control 16.9 (4.6)	2H AUC Intervention ATG 0.88 Control 0.97	ATG/GCSF placebo (2.5 mg/kg as two divided intravenous infusions of 0.5 mg/kg and 2 mg/kg)	Placebo (delivered as intervention)	1 day/1 day	AUC; Meal stimulated	2H AUC	0, 6, 9, 12, 18, 24,	HbA1c; Insulin dose; Hypoglycaemia; Adverse events
**Haller 2014b ** [50]** (3, A, AD)** *Also listed in GCSF group due to combination* > *4 months (but mean duration 12 months)*	25 (17; 8)	Whole study population 12 (6) months	Intervention 24.7 (9.9) Control 24.5 (11)	2H AUC Intervention 0.71 Control 0.71	Combination ATG/GCSF (2.5 mg/kg given intravenously as 0.5 mg per kilogram on day 1 and 2 mg/kg on day 2))	Placebo (delivered as intervention)	12 weeks (GCSF)/12 weeks	AUC, Peak stimulated; Meal stimulated	2H, 4H AUC, peak stimulated (timepoint NR)	0, 6, 12, 18, 24, 30, 36, 42, 48, 54, 60	HbA1c; Insulin dose; Hypoglycaemia; Adverse events
**Chujo ** [51]** 2023 (1, A)** *Conference abstract only* *Within 12 months*	12 (6; 6)	NR	NR	NR	ATG/pegfilgrastim (2.0 mg/kg ATG and six times of 3.5 mg PEG)	Described only as “control”	NR	AUC; Meal stimulated	AUC time NR	NR	HbA1c; Insulin dose; Adverse events
**Granulocyte colony-stimulating factor (GCSF)** *(Haller 2014b and Haller 2018 are also in the ATG group)*											
**Haller 2014a ** [52]** (2, AD, A)** *Within 6 months*	21 (14; 7)	Intervention 3.74 (SAE 0.61) months, control 4.12 (SE 0.81) months	Intervention 17.61 (7.8) Control 19.34 (6.3)	2H AUC Intervention 0.51 Control 0.84	Pegylated GCSF (6 mg/dose, every 2 weeks)	Placebo (delivered as intervention)	10 weeks/10 weeks	Peak stimulated, AUC; Meal simulated	2H AUC, peak time NR	0, 6, 9, 12	HbA1c; Insulin dose; Adverse events
**Haller 2014b ** [50]** (3, A, AD)** *Also listed in ATG group due to combination* > *4 months (but mean duration 12 months)*	25 (17; 8)	Whole study population 12 (6) months	Intervention 24.7 (9.9) Control 24.5 (11)	2H AUC Intervention 0.71 Control 0.71	Combination ATG/GCSF (GCSF 6 mg subcutaneously every 2 weeks for a total of 6 doses)	Placebo (delivered as intervention)	12 weeks (GCSF)/12 weeks	AUC, Peak stimulated; Meal stimulated	2H, 4H AUC, peak stimulated (timepoint NR)	0, 6, 12, 18, 24, 30, 36, 42, 48, 54, 60	HbA1c; Insulin dose; Hypoglycaemia; Adverse events
**Haller 2018 ** [49]** (2, A, AD)** *Also listed in ATG group. 3-arm trial with ATG/GCSF combination group and ATG alone group* *Within 100 days*	89 (ATG 29, ATG/GCSF 29; 31)	ATG/GCSF median 83 (range 49–97) days, ATG 81 (range 47–100) days, control 84 (range 52–99) days	Intervention ATG/GCSF 17.2 (5.0) Control 16.9 (4.6)	2H AUC Intervention ATG/GCSF 0.8 Control 0.97	Combination ATG/GCSF (GCSF 6 mg every 2 weeks for 6 doses or, if the patient weighed < 44.5 kg, a dose of 100 mg/kg)	Placebo (delivered as intervention)	1 day/1 day	AUC; Meal stimulated	2H AUC	0, 6, 9, 12, 18, 24,	HbA1c; Insulin dose; Hypoglycaemia; Adverse events
**IL-1**											
**Hessner 2013 ** [53]** (1, AD, A)** *Within 12 weeks*	47 (22; 25)	NR	NR	NR	Human recombinant (h) IL-1 receptor antagonist (100 mg, once daily)	Placebo (delivered as intervention)	9 months/9 months	AUC; Meal stimulated	2H AUC	9	N/A
**Moran 2013a ** [54]** (1, MI)** *Within 100 days*	69 (47; 22)	Intervention 75.8 (17.9) days, control 75.6 (21.8) days	Intervention 11 (4.0) Control 12.5 (6.4)	2H AUC Intervention 0.63 Control 0.61	Canakinumab (2 mg/kg, max 300 mg, monthly) [binds IL1beta]	Placebo (delivered as intervention)	12 months/12 months	AUC; Meal stimulated	–10, 0, 15, 30, 60, 90, and 120 min, AUC	0, 6, 9, 12	HbA1c; Insulin dose; Adverse events
**Moran 2013b ** [54]** (1, MI)** *Both Moran studies are the same paper, but report 2 separate trials* *Within 12 weeks*	69 (35;34)	Intervention 64.2 (18.0) days, control 59.8 (17.1)	Intervention 26.6 (5.3) Control 25.0 (4.5)	2H AUC Intervention 0.63 Control 0.7	Anakinra (100 mg daily) [stimulates IL1alpha and beta]	Placebo (delivered as intervention)	9 months/9 months	AUC; Meal stimulated	−10, 0, 15, 30, 60, 90, and 120 min, AUC	0, 6, 9	HbA1c; Adverse events
**Mesenchymal stem cell (MSC)**											
**Hu 2013 ** [55]** (1, MI)** *Within 6 months*	29 (15;14)	NR	Intervention 17.6 (8.7) Control 18.2 (7.9)	Fasting Intervention 0.27 Control 0.29	Intravenous delivery of Wharton’s jelly-derived mesenchymal stem cells (2 doses, 4 weeks apart. Dose NR)	Placebo (delivered as intervention)	1 month/1 month	Fasting; Meal stimulated	0	0, 6, 9, 12, 15, 18, 21, 24	HbA1c; Insulin dose; Adverse events
**Carlsson 2015 ** [56]** (1, A)** *Within 3 weeks*	20 (10; 10)	NR	Intervention 24 (2) Control 27 (2)	Fasting Intervention 0.29 Control 0.28	Intravenous infusion of Mesenchymal Stromal Cells (2.1–3.6 × 10^6^ autologous cells/kg, one dose)	Control group (no further details given)	One dose/NR	Fasting; Meal stimulated	0, 15, 30, 60, 90, 120, AUC	10 weeks, 12 months	HbA1c; Insulin dose; Adverse events
**Izadi 2022 ** [57]** (2, MI)** *Within 6 weeks*	21 (11; 10)	NR	Intervention 10.27 (1.67) Control 11.5 (2.63)	Measure of c-peptide NR Intervention 0.24 Control 0.31	Intravenous infusion of Mesenchymal stem cell (MSC) (1 × 10^6^ autologous MSCs per kilogram of body weight, in two infusions each at week 0 and 3)	Placebo (Saline delivered as intervention)	3 weeks/3 weeks	NR	NR	0, 6, 9, 12	HbA1c; Glucose variability; Insulin dose; Hypoglycaemia; Quality of life; Adverse events
**Carlsson 2023 ** [58]** (2, A)** *Within 2 years*	15 (10; 5)	Intervention median 1 (SD 0.7) years, control 1 (SD 0.3) years*	Median (SD) Intervention 31 (4) Control 31 (9)*	2H AUC (median) Intervention 0.77 Control 0.4	Intravenous infusion of Wharton’s jelly mesenchymal stromal cell (ProTrans) (200 million cells procured from donated Wharton’s jelly tissue and expanded in adherent culture over approximately 4–5 weeks. One dose)	Placebo (Excipients as the intervention without cell product, delivered as intervention)	One dose/one dose	Fasting; Meal stimulated	0, 15, 30, 60, 90, 120 min, AUC	0, 12	HbA1c; Glucose variability; Insulin dose; Hypoglycaemia; Adverse events
**Azathioprine**											
**Silverstein 1988 (1, MI)** *Also in the Prednisolone group)* *Within 2 weeks*	46 (23; 23)	NR	Intervention 11.7 (NR) Control 10.4 (NR)	Peak c-peptide Intervention 0.4 Control 0.41	Combination Prednisolone/Azathioprine (azathioprine 2 mg/kg, adjusted to 1–3 mg/kg/day, max 150 mg/day, daily)	Control (Unclear, appears to be no treatment)	12 months/NR	Peak; Meal stimulated	0, 30, 60, 90, 120 min	0, 12	HbA1c; Insulin dose; Clinical remission; Adverse events
**Cook 1989 ** [59]** (1, MI)** *Within 20 days*	50 (25;25)	NR	Intervention 11.7 (NR) Control 9.9 (NR)	Fasting (Median) Intervention 0.14 Control 0.14	Azathioprine (2 mg/kg day 1. Dosage adjusted according to increases in body weight. Daily)	Placebo (delivered as intervention)	12 months/12 months	Fasting, Peak; Meal stimulated	0, 60 min	0, 6, 9, 12	HbA1c; Insulin dose; Clinical remission; Adverse events
**Harrison 1985 ** [60]** (1, AD, A)** *Within 12 weeks*	24 (13; 11)	Intervention 2.2 (1.5) weeks, control 3.5 (3.5) weeks	Intervention 24.7 (7.5) Control 24.8 (7.5)	Fasting Intervention 0.42 Control 0.31	Azathioprine (2 mg/kg, daily)	No treatment	12 months/N/A	Fasting, Peak; Glucagon stimulated	0, 6 min	0, 12	Insulin dose; Clinical remission; Adverse events
**Anti-Immunoglobulin**											
**Panto 1990 ** [61]** (1, MI)** *Within 6 weeks*	16 (8; 8)	Whole study population 45.3 (9 days)	Intervention 19 (2) Control 14.1 (2.7)	Non-fasting Intervention 0.23 Control 0.36	7S immunoglobulin (0.4 g/kg body weight, once weekly after 1 intensive week)	No treatment	6 months/N/A	Non-fasting	NR	0, 6	HbA1c; Insulin dose;
**Lorini 1991 ** [62]** s(1, C, AD)** *“Newly diagnosed”,*	25 (10; 15)	Mean duration of symptoms intervention 20 (16) days, control 21 (17) days	Intervention 7.9 (3.9) Control 9.2 (3.4)	Glucagon stimulated 6 min Intervention 0.36 Control 0.32	Gamma-globulin (400 mg/kg body weight, daily first week, then once a week up to 6 months, then twice a month up to 12 months)	No treatment	12 months/N/A	Max simulated; Glucagon stimulated	6 min	0, 6, 7.5, 9, 10.5, 12	HbA1c; Insulin dose; Clinical remission; Adverse events
**Colagiuri 1996 ** [63]** (1, C, AD)** *3 arm trial* *Within 2 years, but mean duration 24 weeks*	52 (Gammaglobulin 17; transfer factor 18; 17)	Gammaglobulin 155 (148) days, transfer factor 171 (196) days, control 176 (107) days	Gammaglobulin 16.6 (7.8) Control 17.6 (7.1)	Fasting Intervention (Immunoglobulin) 0.22 Control 0.32	Gammaglobulin group (2 g/kg body weight, given over 2 days every 2 months)	Placebo (saline, delivered as intervention)	24 months/24 months	Fasting; Glucagon stimulated, Meal stimulated	0, 120, 300 min	0, 6, 12	HbA1c; Insulin dose; Adverse events
**Interferon α**											
**Rother 2009 ** [64]** (1, MI)** *3 arm trial* *Within 6 weeks*	128 (5000 units 39, 30,000 units 45; 44)	NR	Intervention 5000 units 10.5 (5.1) 30,000 units 9.9 (4.4) Control 10.8 (4.8)	Fasting 5000 units 0.39 30,000 units 0.5 Control 0.46 2H AUC 5000 units 0.91 30,000 units 0.85 Control 0.89	Interferon-α (5000 or 30,000 units, once daily)	Placebo (saline with 6 mg human serum albumin, daily)	12 months/12 months	Fasting, AUC; Meal stimulated	–10, 0, 30, 60, 90, 120 min, and AUC	0, 6, 9, 12	HbA1c; Insulin dose; Adverse events
**IL2 (Daclizumab) + Mycophenolate mofetil (MMF)**											
**Gottlieb 2010 ** [65]** (1, MI)** *3 arm trial* *Within 3 months*	126 (MMF/daclizumab 41, MMF/daclizumab placebo 31; 42)	Whole study population 76 days	Intervention MMF/daclizumab 18.3 (9.1) MMF/daclizumab placebo 17.1 (6.7) Control 18.8 (10.5)	2H AUC MMF/Daclizumab 0.71 MMF/Daclizumab placebo 0.65 Control 0.71	MMF/Daclizumab (Daclizumab 1 mg/kg at study day 0 and 2 weeks later)	Placebo (delivered as intervention)	2 weeks/2 weeks	AUC; Meal stimulated	15–30 min intervals, 2H AUC	0, 6, 12, 18, 24	HbA1c; Insulin dose; Adverse events
**Prednisolone**											
**Secchi 1990 ** [66]** (1, A)** *3 arm trial (Also in the wider “others group of interventions, focus only on prednisolone group here)* *Within 8 weeks*	25 (Prednisolone 10, indomethacin 5; 10)	Prednisolone 4.0 (SE 0.9) weeks, control 4.0 (SE 0.8) weeks	Prednisolone 26 (7.9) Control 23 (4.7)	NR	Prednisolone (15 mg daily)	Placebo (delivered as intervention)	8 months/8 months	Urinary c-peptide only	N/A	0, 6, 9, 12, 18, 24, 30, 36	HbA1c; Insulin dose; Clinical remission; Adverse events
**Hehmke 1994 ** [67]** (1, C, AD)** *(Also in the Anti-CD4 group)* *Within 1 week*	11 (5; 6)	NR	Intervention 12 (2) Control 15 (1)	NR	Prednisolone and Anti-CD4 (Pred 1.0 mg/kg, 5 days intravenous, 5 days oral)	Conventional insulin therapy only	10 days/N/A	Fasting; Stimulation NR	NR	6, 9, 12	HbA1c; Insulin dose; Clinical remission; Adverse events
**Silverstein 1988 ** [68]** (1, MI)** *(Also in the azathioprine group)* *Within 2 weeks*	46 (23; 23)	NR	Intervention 11.7 (NR) Control 10.4 (NR)	Peak c-peptide Intervention 0.4 Control 0.41	Prednisolone/Azathioprine (Pred 30 mg/kg alternate days for 4 doses, then 2 mg/kg, max 100 mg/day. Then tapered every 2 weeks decreasing first to 1 mg/kg/day then to alternate-day administration with a gradual discontinuation in 10 weeks.)	Control (Unclear, appears to be no treatment)	12 months/NR	Peak; Meal stimulated	0, 30, 60, 90, 120 min	0, 12	HbA1c; Insulin dose; Clinical remission; Adverse events
**Anti-CD4**											
**Hehmke 1994 ** [67]** (1, C, AD)** *(Also in the prednisolone group)* *Within 1 week*	11 (5; 6)	NR	Intervention 12 (2) Control 15 (1)	NR	Prednisolone and Anti-CD4 (Anti-CD4 0.5 mg/kg IV 30 min on a 5 consecutive days)	Conventional insulin therapy only	10 days/N/A	Fasting; Stimulation NR	NR	6, 9, 12	HbA1c; Insulin dose; Clinical remission; Adverse events
**Anti-TNF (Golimumab)**											
**Quattrin 2020 ** [69]** (4, C, AD)** *Within 100 days*	84 (56; 28)	Intervention 73.6 (19.6) days, control 75.7 (19.0) days	Intervention 13.9 (3.7) Control 14.0 (4.0)	4H AUC Intervention 0.78 Control 0.64	Golimumab (body weight < 45 kg induction dose 60 mg per square metre body-surface area at weeks 0 and 2, body weight ≥ 45 kg induction dose 100 mg at weeks 0 and 2. Maintenance dose 30 mg/50 mg per square metre week 4 and every 2 weeks until 52 weeks)	Placebo (delivered as intervention)	12 months/12 months	AUC; Meal stimulated	2H and 4H AUC	0, 6, 9, 12, 18, 24	HbA1c; Insulin dose; Hypoglycaemia; Ketoacidosis; Clinical remission; Adverse events
**Tyrosine kinase Inhibitor**											
**Gitelman 2021 ** [70]** (1, A)** *Within 100 days*	67 (45; 22)	Intervention median 82 (IQR 70–91) days, control 94 (IQR 73–96) days	Intervention median 26.6 (IQR 22.4–32.4) Control median 23.4 (IQR 21.6–29.9)	4H AUC Intervention 0.74 Control 0.68	Imatinib (4 × 100 mg film-coated tablets per day)	Placebo (delivered as intervention)	6 months/6 months	AUC; Meal stimulated	0, 15, 30, 60, 90, 120 min; 2H and 4H AUC	0, 6, 12, 18, 24,	HbA1c; Insulin dose; Hypoglycaemia; Ketoacidosis; Body weight; BMI; Adverse events
**Anti-IL 6 receptor**											
**Greenbaum 2021 ** [71]** (1, MI)** *Within 100 days*	Adults 55 (35; 20) Paediatric 81 (54; 27)	Adults intervention 82.5 (13.63) days, control 84.6 (12.39) days Paediatric intervention 85.9 (15.47) days, control 83.9 (16.65) days	Adults Intervention 27.9 (7.37) Control 29.2 (9.33) Paediatric Intervention 11.1 (2.90) Control 11.1 (2.47)	2H AUC Adults Intervention 0.77 Control 0.97 Paediatric Intervention 0.73 Control 0.66	Toclizumab (≥ 30 kg body weight, 8 mg/kg to a maximum of 800 mg. < 30 kg, the dose was 10 mg/kg, every 4 weeks for 7 doses)	Placebo (delivered as intervention)	6 months/6 months	AUC; Meal stimulated	–10 and 0, then at min 15 and 30, and then every 30 min for 2 h, AUC	0, 6, 9, 12	HbA1c; Insulin dose; Hypoglycaemia; Ketoacidosis; Adverse events
**Anti-IL 8 receptor**											
**Piemonti 2022 ** [72]** (3, AD, A)** *Within 100 days*	76 (50; 26)	NR	Intervention 27.6 (7.06) Control 8 (6.35)	Fasting Intervention 0.22 Control 0.23 2H AUC Intervention 0.5 Control 0.49	Ladarixin (400 mg twice daily for three cycles of 14 days on/14 days off)	Placebo (delivered as intervention)	3 months/3 months	Fasting, Peak stimulated, AUC; Meal stimulated	0, 15–120 min, 2H AUC	0, 6, 12	HbA1c; Insulin dose; Hypoglycaemia; Adverse events
**Regulatory T-cell (Low dose IL-2, Aldesleukin)**											
**Rosenzwajg 2020 ** [73]** (1, C, AD)** *4 arm trial* *Within 3 months*	24 (0.125MIU 5, 0.25MIU 6, 0.5MIU 6; 7)	NR	Intervention 0.125MIU 10.6 (1.1), 0.25MIU 9.7 (1.6), 0.5MIU 10.2 (2) Control 9.3 (1.4)	Fasting Intervention 0.125MIU 0.33, 0.25MIU 0.3, 0.5MIU 0.33 Control 0.232H AUC Intervention 0.125MIU 0.62, 0.25MIU 0.57, 0.5MIU 0.6 Control 0.48	IL-2 Aldesleukin (0.125, 0.25 or 0.5 MIU m^−2^ day^−1^ based on body surface area)	Placebo (delivered as intervention)	12 months/12 months	Fasting, AUC; Meal stimulated	0, 2H AUC	0, 6, 12, 14	HbA1c; Glucose variability; Insulin dose; Hypoglycaemia; Adverse events
**Regulatory T-cell (Tregs)**											
**Zielinski 2022 ** [45]** (2, C, AD)** *(Also included in the B-cell group)* *NR, mean duration 6 months*	25 (Tregs/rituximab 12; Tregs/Placebo 13)	Tregs/Rituximab 6 (4.2) months, Tregs/Placebo 6.5 (4.2) months	Intervention (Tregs/Rituximab) 12.9 (1.2) Control (Tregs/Placebo) 13.3 (1.5)	4H AUC Intervention 0.91 Control 0.836 Fasting Intervention 0.36 Control 0.36	Tregs/Rituximab (Tregs 30 × 10^6^ of Tregs/kg body weight each, 2 doses 3 months apart)	Tregs/Placebo (2 doses of Tregs, 30 × 10^^^6Tregs/kg body weight; each at day 0 and month 3 and placebo delivered as intervention)	36 days/36 days	Fasting, AUC; Meal stimulated	0, 15, 30, 60, 90, 120, 180, 210 and 240 min, 4H AUC	Weeks26, 39, 52, 65, 78, 92 and 104	HbA1c; Insulin dose; Hypoglycaemia; Ketoacidosis; Clinical remission; Adverse events
**Bender 2024 ** [74]** (3, C, AD)** *Within 100 days*	131 (low dose 38; high dose 37; placebo 39)	Intervention low dose 85.12 (13.43) days, high dose 86.54 (14.69) days, control 85.15 (13.83)	Intervention low dose 13.49 (2.48) High dose 13.78 (2.86) Control 13.67 (2.46)	2H AUC Intervention low dose 0.75 High dose 0.78 Control 0.72	Polyclonal autologous Tregs (IV infusion of 1 × 106 to 7 × 106 cells/kg of body weight, or 11 × 106 to 24 × 106 cells/kg of body weight)	Placebo (Infusion solution only: 0.5% human albumin USP in an equal mixture of USP PlasmaLyte-A and USP 5% dextrose/0.45% NaCl; delivered as intervention)	One infusion/one infusion	AUC; Meal stimulated	2 and 4H AUC	0, weeks 26, 52, and 104 for 4 h and weeks 13 and 78 for 2 h	HbA1c; Insulin dose; Hypoglycaemia; Ketoacidosis; Adverse events
**IL-21**											
**Von Herrath 2021 ** [75]** (3, A)** *(3 arm trial, focus given to those which included Anti IL-21)* *Within 20 weeks*	308 (Anti IL-21/liraglutide 77, Anti IL-21/placebo 63, liraglutide/placebo 77; 77)	Intervention Anti IL-21/liraglutide 11.6 (5.7) weeks, Anti IL-21/placebo 11.5 (5.3 weeks), liraglutide/placebo 10.8 (4.8) weeks, control 10.2 (4.7) weeks	Intervention Anti IL-21/liraglutide 28 (7.5), Anti IL-21/placebo 28.6 (7.9), liraglutide/placebo 28 (7.1) Control 29 (7.0)	Fasting Intervention Anti IL-21/liraglutide 0.13 Anti IL-21/placebo 0.23 Control 0.23 4H AUC Intervention Anti IL-21/liraglutide 0.48 Anti IL-21/placebo 0.53 Control 0.56	Anti IL-21 and liraglutide (Anti IL-21 12 mg/kg every 6 weeks)	Placebo (matching Anti IL-21 and liraglutide delivered as intervention)	12 months/12 months	Fasting, AUC; Meal stimulated	(0), and at 15, 30, 60, 90, 120, 150, 180, 210 and 240 min, 2 and 4H AUC	0, 6, 9, 12, 15, 18	HbA1c; Insulin dose; Glucose variability; Hypoglycaemia; Ketoacidosis; Body weight; Adverse events
**Deflazacort**											
**Pozzilli 1994 ** [76]** (1, MI)** *Within 4 weeks*	36 (18; 18)	NR	Intervention Adult 25.1 (5.7) children 11. (1.5) Control Adult 23.2 (4.8) Children 11 (1.7)	Fasting Intervention (Deflazacort and nicotinamide) 0.17 Control (Nicotinamide and placebo) 0.2	Deflazacort and nicotinamide (Deflazacort 0.6 mg/kg body weight daily for 1 month, reduced to 0.3 mg/kg of body weight daily for 2 months)	Nicotinamide and placebo (placebo delivered as intervention, nicotinamide 25 mg/kg of body weight daily)	3 months/3 months	Fasting, stimulated; Glucagon stimulated	NR	0, 6, 12	HbA1c; Insulin dose; Clinical remission; Adverse events
**Methotrexate**											
**Buckingham 2000 ** [77]** (1, C, AD)** *Within 11 days*	10 (5; 5)	NR	Intervention 9.8 (SE 0.8) Control 10.7 (SE 0.9)	Meal stimulated 60 min Intervention 0.011 Control 0.014	Methotrexate (5 mg/m^2^ per week)	No methotrexate	36 months/N/A	Fasting, Max stimulated; Meal stimulated	0, 60 min	0	HbA1c; Insulin dose; Clinical remission; Adverse events
**Photophoresis**											
**Ludvigsson 2001 ** [78]** (1, C, AD)** *Within 25 days of symptoms*	49 (25; 24)	NR	Intervention 13.8 (1.6) Control 13.4 (1.9)	Fasting Intervention 0.48 Control 0.58	Photophoresis, (oral 8-methoxypsoralen, 1 h later, apheresis was started, administered on two consecutive days (double treatment). First double treatment given at day 5–6 after diagnosis, then repeated after 2, 4, 8 and 12 weeks so that every patient should receive five double treatments in 3 months)	Placebo (placebo tablets and sham pheresis with saline solution. Same frequency as intervention)	3 months/3 months	Fasting, Stimulated; Meal stimulated, also urinary	0, stimulated timepoint NR	1, 6, 12, 18, 24, 30, 36	HbA1c; Insulin dose; Clinical remission; Adverse events
**Etanercept**											
**Mastrandrea 2009 ** [79]** (1, C, AD)** *Within 4 weeks*	18 (10; 8)	Intervention 21.4 (4.4) days, control 28.5 (7.5) days	Intervention 12.5 (3.3) Control 12.4 (3.6)	2H AUC Intervention 0.51 Control 0.78	Etanercept (0.4 mg/kg up to a maximum dose of 25 mg/dose, twice weekly)	Placebo (no further information)	24 weeks/24 weeks	Fasting, AUC; Meal stimulated	0, 30, 60, and 120 min and AUC	0, 6	HbA1c; Glucose variability; Insulin dose; Adverse events
**Humanised monoclonal antibody IgG1 (Itolizumab)**											
**Cabrera-Rode 2022 ** [80]** (1, NR)** *4 arm trial* *Within 12 weeks*	12 (0.4 mg 3, 0.8 mg 2, 1.6 mg 3; 3)	NR	Reported for all patients only 24.08 (4.68)	Fasting Intervention 0.4 mg 0.35 0.8 mg 0.36 1.6 mg 0.32 Control 0.37 2H AUC Intervention 0.4 mg 0.84 0.8 mg 0.57 1.6 mg 0.7 Control 0.62	Humanised monoclonal antibody Itolizumab (0.4/0.8 mg/kg body weight once a week or 1.6 mg/kg body weight every 2 weeks for 9 weeks then every 4 weeks until 24 weeks of treatment)	Placebo (delivered as intervention)	6 months/6 months	Fasting, AUC; Meal stimulated	0, 30, 60, 90, 120, 2H AUC	0, 12	HbA1c; Insulin dose; BMI; Adverse events
**Humanised monoclonal antibody IgG4 (Temelimab)**											
**Curtin 2020 ** [81]** (1, MI)** *Within 3 years*	64 (43; 21)	NR	Intervention 31.4 (10.2) Control 34.9 (10.1)	2H AUC Intervention 0.47 Control 0.77	Temelimab, humanised IgG4 monoclonal antibody) (6 mg/kg, 6 administrations at 4 week intervals)	Placebo (placebo solution delivered as intervention)	6 months/6 months	AUC; Meal stimulated	0, 90 min peak, 120 min, AUC 2HR	0, 6	HbA1c; Insulin dose; Hypoglycaemia; Adverse events
**Autologous immature dendritic cells**											
**Gaglia 2024 ** [82]** (2, MI)** *Within 12 months*	**27 (16; 11)**	Intervention 7.9 (2.7) months, control 8.8 (2.0) months	Intervention 26.5 (9.0) Control 26.2(6.2)	Intervention 0.53 Control 0.61	AVT001 (Individualised preparation of autologous immature dendritic cells derived from participant’s adherent primary monocytes. 20 ml AVT001 suspension in 10% dimethyl sulfoxide and 12.5% human serum albumin; approximately 10 million synthetic oligopeptide- loaded, CD11cþ immature dendritic cells per dose; monthly for 3 doses)	Placebo (Volume also 20 ml, containing only 12.5%humanserum albumin with 10% dimethyl sulfoxide; delivered as intervention)	3 months/3 months	AUC, Peak; Meal stimulated	4H AUC, Peak time NR	0, 5, 12	HbA1c; Insulin dose; Hypoglycaemia; Adverse events
**Janus kinase inhibitor (Baricitinib)**											
**Waibel 2023 ** [83]** (2, MI)** *Within 100 days*	91 (60; 31)	Intervention median 88 (IQR 70–95) days, control 72 (IQR 59–90) days	Intervention 18.5 (5.7) Control 18.7 (5.9)	2H AUC Intervention median 0.6 Control median 0.67	Baricitinib (4 mg per day orally)	Placebo (Delivered as intervention)	48 weeks/48 weeks	AUC; Meal stimulated	2H AUC	0, 6, 9, 12 months	HbA1c; Insulin dose; Hypoglycaemia; Ketoacidosis; Adverse events
**Humanised monoclonal antibody IgG1k (Ustekinumab)**											
**Tatovic 2024 ** [84]** (3, AD)** *Within 100 days*	72 (47; 25)	NR	Intervention 14.49 (1.78) Control 15.0 (1.63)	2H AUC Intervention 0.89 Control 0.92	Ustekinumab (2 mg/kg body weight if the participant was ≤ 40 kg and 90 mg if > 40 kg; weeks 0, 4, 12, 20, 28, 36 and 44)	Placebo (Delivered as intervention)	44 weeks/44 weeks	Peak, AUC; Meal stimulated	0, 15, 30, 60, 90 and 120 min, AUC	− 2, 28 and 52 weeks	Hba1c; Insulin dose; Glucose variability; Hypoglycaemia; Quality of LifAdverse events

*Abbreviations*: *HbA1c *haemoglobin A1c, *Age categories C *children, *AD *adolescents, *A *adults, *MI *mixed population, *NR *not reported; *N/A *not applicable, *AUC *area under the curve, *ATG Antithymocyte globulin, GCSF *Granulocyte colony-stimulating factor, *BCG *Bacillus Calmette–Guérin, *MMF *Mycophenolate mofetil, *MIU *Milli-international units, *SE *standard error. *Paper only reported median and SD, no further information could be acquired on this

Risk of bias varied (Additional file 1: Table 3), though few studies were considered high risk of bias. Issues with reporting were indicated due to the large number of domains assigned as “unclear,” particularly for blinding of C-peptide measurement which was generally not described (though unlikely to largely impact upon the outcome). Most trials were double-blind resulting in mostly low risk of bias within this domain. There may be issues within this domain as some immunomodulatory drugs may incur side effects that could potentially have led patients to believe they were in a particular group; however, this was unlikely to have impacted upon the outcomes. Over time, it seems that generally reporting and risk of bias have improved, with many newer studies demonstrating low risk across all domains compared to older studies which resulted in uncertainty and/or high risk.

### Pairwise meta-analysis

Results of the pairwise meta-analyses were varied (results not shown). Several interventions demonstrated statistically significantly preserved C-peptide levels at 12 months versus placebo or no treatment. For example, granulocyte colony-stimulating factor (GCSF), anti-CD3 teplizumab, interferon-alpha, anti-CD3 otelixizumab, anti-TNF golimumab, ATG, azathioprine and cyclosporin demonstrated statistically significantly higher C-peptide (various C-peptide measurement methods and varying number of studies contributing). In the few trials that included longer follow-up at 24 months, several interventions showed statistically significantly higher C-peptide levels than comparator (GCSF, ATG, Anti-CD3 teplizumab, anti-TNF golimumab, mesenchymal stem cells (MSC)).

### Network meta-analysis

#### Main analysis

Forty-one trials of 42 interventions (from the wider 60 trials of 53 interventions) were included in the main network analysis of all C-peptide data (Additional file 1: Fig. S1). Some trials could not be included due to uncommon methods of measuring C-peptide, lack of reporting of certainty data (e.g., standard deviations, standard errors, 95% CI) or short follow-up (e.g. less than 12 months) (see Additional file 1: Table 4).

**Fig. 2:**
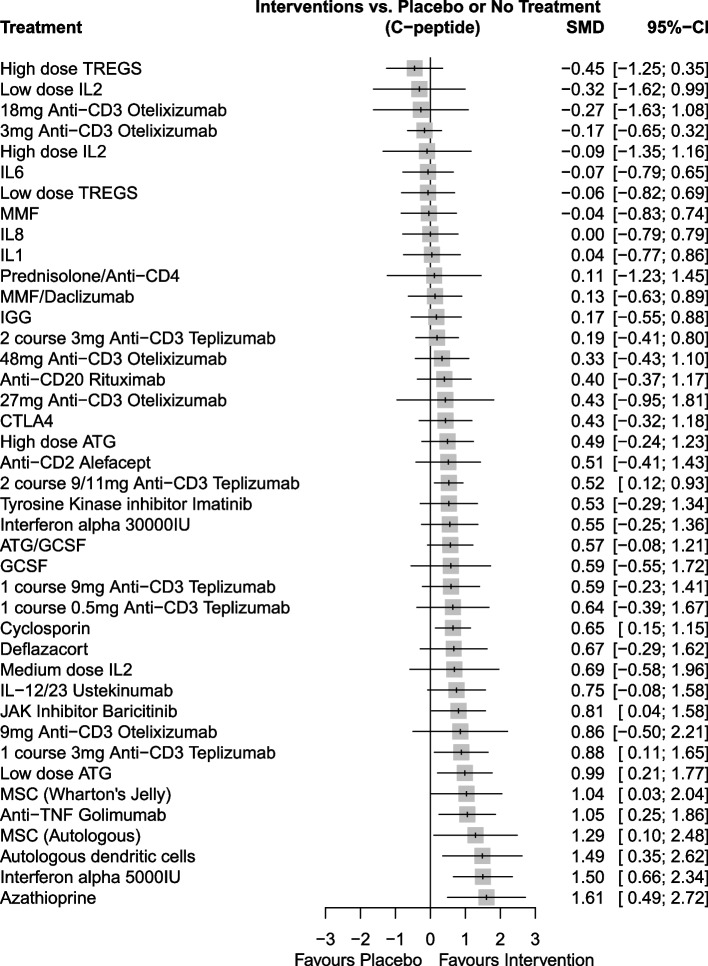
Forest plot of standardised mean differences (95% confidence intervals) in C-peptide of all therapies versus placebo or no comparator included in main network meta-analysis at 12 months. Effect estimates favouring intervention are seen to the right of the line of no effect

Eleven interventions demonstrated statistically significantly higher levels of C-peptide than placebo/no comparator at 12 months; (in order of highest effect size) azathioprine, interferon-alpha (dose of 5000 IU), autologous dendritic cells (ADC), MSC (autologous), anti-TNF golimumab, MSC (Wharton’s Jelly derived), low-dose ATG, 3 mg 1-course anti-CD3 teplizumab, baricitinib, cyclosporin and 9/11 mg 2-course anti-CD3 teplizumab, Fig. 2). All of these interventions, except for 9/11 mg 2-course anti-CD3 teplizumab and cyclosporin, demonstrated > 60% chance of being ranked first, with interferon-alpha (5000 IU) having a 91% chance (frequentist p-scores) and azathioprine a 92% chance (SUCRA scores, Table 2) (see Additional file 1: Fig. S2 for non-cumulative ranking curves). Order of rankings remained comparable between both methods. Whilst median rankings and associated 95% CIs were generally comparable in terms of the higher ranked interventions (Table 2), a high level of uncertainty was present across almost all intervention rankings. Some interventions showed a clear trend towards higher c-peptide levels but were not statistically significant (e.g. Ustekinumab). Therapies that were found to show no evidence of effect were consistently at the bottom of the rankings. It should be noted that rankings should be interpreted with caution as for some therapies data are from smaller pilot or safety trials (e.g. MSC trials) that were not powered to detect efficacy. Some comparisons were also based on data from only one trial (e.g. anti-TNF golimumab, baricitinib, azathioprine, interferon-alpha).

**Table 2:** Probabilities of each intervention being ranked first both as frequentist p-scores and SUCRA scores as well as median rankings and 95% confidence intervals for each intervention based on the outcome of C-peptide at 12 months follow-up

Frequentist *P*-score Rankings		SUCRA Score Rankings		Median rankings and 95% confidence interval		
**Intervention**	***P*-score**	**Intervention**	**SUCRA Score**	**Intervention**	**Median rank**	**95% confidence interval**
Interferon-alpha 5000 IU	0.908	Azathioprine	0.9151	Azathioprine	3	1–14
Azathioprine	0.9072	Autologous dendritic cells	0.908	Autologous dendritic cells	4	1–19
Autologous dendritic cells	0.8804	Interferon-alpha 5000 IU	0.9073	Interferon-alpha 5000 IU	4	1–14
MSC (Autologous)	0.8247	MSC (Autologous)	0.8424	MSC (Autologous)	5	1–26
Anti-TNF Golimumab	0.7807	MSC (Wharton’s Jelly)	0.781	MSC (Wharton’s Jelly)	8	2–28
Low-dose ATG	0.7577	Anti-TNF Golimumab	0.7744	Anti-TNF Golimumab	9	2–25
MSC (Wharton’s Jelly)	0.757	Low-dose ATG	0.7563	Low-dose ATG	9	3–24
1 course 3 mg Anti-CD3 Teplizumab	0.7133	1 course 3 mg Anti-CD3 Teplizumab	0.7276	1 course 3 mg Anti-CD3 Teplizumab	11	4–27
JAK Inhibitor Baricitinib	0.6791	JAK Inhibitor Baricitinib	0.662	9 mg Anti-CD3 Otelixizumab	12	1–37
9 mg Anti-CD3 Otelixizumab	0.666	IL-12/23 Ustekinumab	0.6585	JAK Inhibitor Baricitinib	13	4–30
IL-12/23 Ustekinumab	0.6485	9 mg Anti-CD3 Otelixizumab	0.6202	IL-12/23 Ustekinumab	14	4–31
Cyclosporin	0.6115	Cyclosporin	0.6146	Medium-dose IL2	15	2–39
Deflazacort	0.602	Deflazacort	0.6127	Cyclosporin	16	7–27
Medium-dose IL2	0.6004	Medium-dose IL2	0.5902	Deflazacort	16	3–34
1 course 0.5 mg Anti-CD3 Teplizumab	0.5881	1 course 9 mg Anti-CD3 Teplizumab	0.5773	1 course 0.5 mg Anti-CD3 Teplizumab	17	3–37
1 course 9 mg Anti-CD3 Teplizumab	0.5698	1 course 0.5 mg Anti-CD3 Teplizumab	0.569	1 course 9 mg Anti-CD3 Teplizumab	18	5–34
ATG/GCSF	0.5615	ATG/GCSF	0.5505	GCSF	18	3–38
GCSF	0.5572	Interferon-alpha 30,000 IU	0.5451	ATG/GCSF	19	8–32
Interferon-alpha 30,000 IU	0.5499	Anti-CD2 Alefacept	0.5383	Interferon-alpha 30,000 IU	19	6–35
2 course 9/11 mg Anti-CD3 Teplizumab	0.5449	GCSF	0.5256	Tyrosine Kinase inhibitor Imatinib	19	6–35
Tyrosine Kinase inhibitor Imatinib	0.5371	2 course 9/11 mg Anti-CD3 Teplizumab	0.519	2 course 9/11 mg Anti-CD3 Teplizumab	20	11–29
Anti-CD2 Alefacept	0.5265	Tyrosine Kinase inhibitor Imatinib	0.5024	Anti-CD2 Alefacept	20	5–37
High-dose ATG	0.5208	High-dose ATG	0.4988	High-dose ATG	21	8–35
CTLA4	0.488	CTLA4	0.4778	27 mg Anti-CD3 Otelixizumab	22	3–41
27 mg Anti-CD3 Otelixizumab	0.4849	Anti-CD20 Rituximab	0.4693	CTLA4	22	8–36
Anti-CD20 Rituximab	0.4688	48 mg Anti-CD3 Otelixizumab	0.4437	Anti-CD20 Rituximab	23	9–37
48 mg Anti-CD3 Otelixizumab	0.4346	27 mg Anti-CD3 Otelixizumab	0.4407	48 mg Anti-CD3 Otelixizumab	25	10–38
2 course 3 mg Anti-CD3 Teplizumab	0.3534	Prednisolone/Anti-CD4	0.3927	2 course 3 mg Anti-CD3 Teplizumab	27	15–38
Prednisolone/Anti-CD4	0.3507	IGG	0.3698	IGG	28	13–39
IGG	0.3455	MMF/Daclizumab	0.3571	MMF/Daclizumab	29	14–40
MMF/Daclizumab	0.3317	2 course 3 mg Anti-CD3 Teplizumab	0.3507	Prednisolone/Anti-CD4	29	6–42
IL1	0.2901	High-dose IL2	0.3273	IL1	31	16–41
IL8	0.2679	IL1	0.2744	IL8	32	15–41
High dose IL2	0.2651	MMF	0.2651	Placebo/No treatment	32	29–36
MMF	0.2467	IL8	0.2605	IL6	34	19–41
Low-dose TREGS	0.2387	Placebo/No treatment	0.2385	Low-dose TREGS	34	17–41
Placebo/No treatment	0.2336	IL6	0.2305	MMF	34	17–41
IL6	0.2327	Low-dose TREGS	0.2171	High-dose IL2	35	10–42
18 mg Anti-CD3 Otelixizumab	0.2073	18 mg Anti-CD3 Otelixizumab	0.21	3 mg Anti-CD3 Otelixizumab	36	26–41
Low-dose IL2	0.1921	Low-dose IL2	0.2085	18 mg Anti-CD3 Otelixizumab	38	12–42
3 mg Anti-CD3 Otelixizumab	0.1717	3 mg Anti-CD3 Otelixizumab	0.1666	Low-dose IL2	38	15–42
High-dose TREGS	0.1043	High-dose TREGS	0.1032	High-dose TREGS	39	27–42

The Surface Under the Cumulative Ranking curve (SUCRA) is the rank of treatment *i* within the range of treatments, measured on a scale from 0 (worst) to 1 (best). Frequentist *p*-scores can be interpreted as the mean extent of certainty that treatment *i* is better than another treatment. Interpretation of both scores is comparable

#### Low risk of bias analysis

Twenty-one trials of 29 treatments were included in the subset of trials from the main analysis that were rated low risk of bias. Only two interventions still demonstrated statistically higher C-peptide levels than placebo/no comparator at 12 months: interferon-alpha (5000 IU) and ADC. MSC (Wharton’s Jelly), low-dose ATG, anti-TNF golimumab, baricitinib, ustekinumab and 9/11 mg 2-course anti-CD3 teplizumab showed an improvement versus comparator, but these comparisons were not statistically significant. This was following removal of trials contributing data to some of these comparisons. Interferon-alpha (5000 IU) and ADC were consistently the highest ranked therapies (median rank 3, 95% CI 1–12 and 3 (95% CI 1–14) respectively, Additional file 1: Table 5). However, there was large uncertainty around most of the rankings. Azathioprine and cyclosporin were removed from this analysis.

#### Secondary outcomes

Thirty-five trials from the main C-peptide analysis were included in the analysis of insulin dose and 37 in the analysis of HbA1c. Most studies found no evidence of a difference in daily insulin dose with interventions when compared to placebo/no treatment (Additional file 1: Fig. S3). CTLA4, anti-TNF golimumab, baricitinib and both 1 course 3 mg dose and 2 course 9/11 mg dose anti-CD3 teplizumab therapies showed a small but statistically significant reduction in daily insulin dose versus comparator at 12 months. Several interventions showed a trend towards a small reduction in insulin dose, but this was not significant. CTLA4 reduced daily insulin dose by − 0.18 units/kg/day (95% CI − 0.35 to − 0.01), median rank 5 (95% CI 1–21, Additional file 1: Table 6) but did not demonstrate any significant difference in C-peptide levels.

Several interventions demonstrated a reduction in HbA1c, but only two were statistically significantly lower than placebo or no comparator (mean difference low-dose ATG − 0.91% (− 9.9 mmol/mol), 95% CI − 1.59 (− 17.4 mmol/mol) to − 0.22% (− 2.4 mmol/mol); CTLA4 − 0.57% (− 6.2 mmol/mol), 95% CI − 1.12 (− 12.2 mmol/mol) to − 0.02% (0.2 mmol/mol), Additional file 1: Fig. S4). MSC, autologous and Wharton’s jelly-derived, both had a median rank of third (95% CIs 1–19 and 1–35 respectively), with low-dose ATG fifth (95% CI 2–14, Additional file 1: Table 7). This suggests that MSC and low-dose ATG may be effective in reducing HbA1c and maintaining levels of C-peptide, though data from the MSC interventions were based on individual trials with small sample sizes.

When only trials with low risk of bias were included in the analysis of secondary outcomes, few differences were noted in the findings (Additional file 1: Figs. S5 and S6).

#### Heterogeneity and bias

In the main analysis of all available C-peptide data, moderate to high heterogeneity was present (*I*^2^ 66.5%, 95% CI 34.5–82.1%, tau^2^ = 0.101). There was also clear evidence of inconsistency across the network (design-by-treatment interaction *p* = 0.004). When only those trials which were considered low risk of bias were included in the analysis, heterogeneity was not attenuated (*I*^2^ 94%, 95% CI 81–98.1%, tau^2^ = 0.232) and in fact increased. There was also further evidence of inconsistency (*p* = < 0.001). Detailed results for each analysis are available in Additional file 1: Table 8.

In the analysis of secondary outcomes HbA1c and insulin dose, there was no evidence of heterogeneity or inconsistency.

#### Sensitivity analyses

Planned sensitivity analyses resulted in few differences in highest ranked interventions, with MSC, ADC, baricitinib, interferon-alpha 5000 IU, anti-TNF golimumab, anti-CD3 teplizumab (2 course 9/11 mg dose) and low-dose ATG remaining near the top and consistently demonstrating significantly higher levels of C-peptide than comparator at 12 months. When considering only 2H and 4H AUC (the most highly correlated measures of C-peptide), interferon-alpha 5000 IU, ADC, MSC (Autologous), anti-TNF golimumab, low-dose ATG, both 1 course 3 mg and 2 course 9/11 mg anti-CD3 teplizumab and JAK inhibitor baricitinib continued to show statistically significantly higher C-peptide levels compared to control. In this analysis, some interventions were removed completely due to measuring C-peptide via alternative approaches. Furthermore, there was sometimes less certainty in these analyses, often due to removal of contributing trials. Heterogeneity remained moderate to high when removing trials with baseline imbalances in C-peptide, though was demonstrably lower when including only endpoint data. Further results from sensitivity and post hoc analyses can be found in the Additional file 1: Figs. S7 – S11, Tables 9–13.

#### Adverse events

Adverse events varied (see Additional file 1: Table 14) and were sometimes poorly reported. In terms of interventions showing statistically significantly higher C-peptide levels and found to be ranked as most effective, no significant differences were found in number of events between groups in the anti-TNF golimumab trial and the interferon -alpha trial. In the ATG trials, minor events were noted within the first month in the ATG arm (e.g. cytokine release syndrome) but these generally did not persist. Findings were similar in studies of anti-CD3 teplizumab; some cases of transient lymphopenia were noted, but this was expected due to the mechanism of action. Trials of azathioprine, MSC (both autologous and Wharton’s jelly derived), baricitinib and cyclosporin reported no serious side effects that were thought to be related to the intervention, though some minor events were observed.

## Discussion

### Summary of findings

This systematic review and network meta-analysis identifies eleven interventions that demonstrate efficacy in preserving beta cells and provides a ranking of the relative effectiveness of these therapies. Ten of these eleven therapies demonstrated > 60% chance of being ranked first; however, given the wide confidence intervals of median rankings, there is uncertainty in exact rank order of near adjacent therapies. Therefore, findings should be considered as hypothesis generating to inform future trials.

### Strengths and limitations of the evidence

Whilst the evidence included in this review are vast, trials contributing to the NMA were sometimes limited. For example, only one published trial has been undertaken of anti-TNF golimumab [69], baricitinib [83], MSC interventions [55, 56] and interferon- alpha [64]. The sample size was small in four of the intervention trials (both MSC trials, ADC and azathioprine), though it is unjustifiable to exclude these trials based on sample size alone as this may introduce further issues to the analysis. Data for most interventions stems from larger sample sizes. However, the results of the analysis should be considered as hypothesis generating. There were also only eleven direct head-to-head trials. Planned and ongoing trials may add value to the network in the future and also demonstrate the importance of continuing research in this field [85].

Dosing of interventions is also an important consideration. For example, anti-CD3 teplizumab groups with different cumulative doses (mg/m^2^) and different course lengths (one or two during the trial) were analysed separately. Only some of these doses demonstrated statistically significantly better C-peptide levels and there were no trials delivering the same dosing strategy as approved by the FDA for the prevention of stage 3 type 1 diabetes [86]. In some interventions, increasing dose appeared to have a greater effect, whereas in others, increasing dose worsened the effect, reiterating the importance of dose-finding studies.

Heterogeneity was often substantial across different analyses which may be due to differences in populations or other clinical or methodological characteristics. Whilst we considered this in our planning, we could not account for the levels of heterogeneity observed in sensitivity analyses by risk of bias and baseline imbalances. There was also evidence of inconsistency within the network in some of the analyses.

#### Strengths and limitations of the review

This is the most comprehensive systematic review of its kind, including more trials and interventions than previous reviews. The NMA approach is novel in this field and is key for identifying which interventions are the most likely to be effective and should be studied further, from a large pool of clinical trial evidence.

Efforts were made to address potential effect modifiers (e.g. baseline C-peptide and age) via sensitivity analyses and assessment of correlations of effect modifiers with treatment effects. However, it would be difficult to consider all possible effect modifiers.

There are also issues interpretating SMDs, as the units are standard deviations so determining the clinical relevance of effect sizes is difficult. Following Cochrane Handbook guidance on re-expressing SMDs, when thresholds were applied to group SMDs into small, moderate or large effects, most of the statistically significant interventions demonstrated “large” effects. Further approaches recommended by Cochrane were deemed inappropriate and either inflated or underestimated findings of individual trials. Even in its natural units, C-peptide may be difficult to interpret, particularly relating it to clinically important effects. However, recent evidence supports C-peptide as an appropriate surrogate outcome measure for therapeutic efficacy in trials of newly diagnosed type 1 diabetes patients, with the FDA recently acknowledging C-peptide as a surrogate endpoint to predict clinical benefit [87, 88].

To incorporate as many trials as feasibly possible, we used both difference between endpoint and difference between change from baseline data in the SMD meta-analyses. Some could consider this a limitation; however, the approach is supported by recent evidence [89, 90] and was mitigated through sensitivity analyses.

Whilst we included 60 trials in this review, only 41 were eligible for inclusion in the NMA due to poor reporting and differences in C-peptide measurement methods. There may also be limitations when combining different C-peptide measurements within this analysis due to the differences in stimulation (e.g. glucagon vs mixed meal or fasting) or duration of measurement (e.g. 2-h or 4-h AUC, peak at different 90 or 120 min). This may result in varied results even in similar populations. However, as noted previously, many studies demonstrate that most of these measures are highly correlated [18, 19]. Sometimes there were differences in findings between studies of the same interventions included in the NMA and those not eligible. For example, two azathioprine studies not included in the NMA reported unclear findings, whereas a further MSC (Wharton’s Jelly derived) study ineligible for inclusion agreed with the NMA findings. Measurement and reporting of C-peptide should be standardised in future research to aid in evidence synthesis.

Finally, it could be questioned whether differences in HbA1c between treatment arms reflect the success of immunomodulatory therapies due to improved patient care via optimal glycaemic control through standard care. However, in a well-designed, blinded RCT, both intervention and control groups follow the same standardised diabetes management protocols and, consequently, any differences observed in outcomes such as HbA1c, or insulin dose are more likely attributable to the immunomodulatory intervention itself.

### Findings in context

Some of the eleven therapies identified in this review as being potentially effective have been identified in other reviews, e.g. teplizumab, low-dose ATG, golimumab (Gandhi, Felton, Jacobson) [6–8]. However, this NMA included new therapies from recently published trials, identified older therapies not previously considered (e.g. azathioprine), has considered the effect of dose, and furthermore, has been able to rank effectiveness. Therapies such as azathioprine may have previously been dismissed due to side effects or a belief of minimal incremental benefits when patients are already on insulin therapy. For example, Lin 2024 excluded azathioprine from their analysis. However, they are used in the management of other immune disorders. It is also noteworthy that many of the therapies (anti-CD3 teplizumab, ATG, cyclosporin, azathioprine) directly target T cell function, a central cell in type 1 diabetes pathogenesis.

Whilst this analysis has identified some of the more effective therapies for beta cell preservation, the benefits to glucose control and insulin dose were not consistent. CTLA4 demonstrated the strongest significant effect on HbA1c but showed no C-peptide benefit, and the C-peptide benefit of anti-CD3 teplizumab translated only mildly to HbA1c and insulin dose. However, this review focusses on C-peptide and will have skewed study selection to RCTs with a C-peptide focus.

Other factors that will influence choice of agent include mode of administration and duration of treatment. Whilst intravenous infusion of MSC may be the highest-ranking therapy in this NMA, the practicalities of administration are likely more complex than that of taking a tablet of azathioprine. Similarly, the short administration of teplizumab is likely to be more acceptable to patients than repeated anti-TNF infusion. Therefore, studies of acceptability with patients and healthcare professionals, and review of long-term safety will be important aspects to consider going forward and will support decisions on which interventions should be prioritised in future trials.

C-peptide preservation will be able to prevent type 1 diabetes in those predisposed. Indeed, anti-CD3 teplizumab is now licenced by the FDA for type 1 diabetes prevention [91]. The results of our review potentially inform which other therapies could be investigated in this space.

## Conclusions

Whilst several interventions were found to preserve beta cell function compared to placebo/no treatment at 12 months, more research is needed. These results should be interpreted with caution, due to uncertainty in some statistical findings and potential issues with heterogeneity and inconsistency. Rather than recommend interventions for practice, these findings highlight the interventions that should be the focus of future head-to-head, high-quality clinical trials with the goal of finding the best therapies for treating newly diagnosed type 1 diabetes. The difference across dose ranges suggests that dose finding studies also need to be considered for new agents and before larger studies are conducted.

## Supplementary Information

Additional file 1.

## Data Availability

The datasets used and/or analysed during the current study are available from the corresponding author on reasonable request.

## Abbreviations

ADC: Autologous dendritic cells
ATG: Anti-thymocyte globulin
FDA: US Food and Drug Administration
GCSF: Granulocyte colony-stimulating factor
IU: International unit
MSC: Mesenchymal stem/stromal cells
NMA: Network meta-analysis
SMD: Standardised mean difference
SUCRA: Surface under the cumulative ranking curve
