# Supplementary material for: A systematic review and network meta-analysis of interventions to preserve insulin-secreting beta cell function in people newly diagnosed with type 1 diabetes: results from randomised controlled trials of immunomodulatory therapies
Source: BMC Med. 2025 Jul 1;23:351. doi: 10.1186/s12916-025-04201-z (PMC12211534; doi:10.1186/s12916-025-04201-z)
Supplement: Supplementary file 1 — Additional file 1. [file 12916_2025_4201_MOESM1_ESM.docx]

**Supplementary Material**

Contents

[Search Strategies 2](#_Toc198210090)

[Search strategy for MEDLINE database 2](#_Toc198210091)

[Search strategy for Embase database 3](#_Toc198210092)

[Search strategy for Cochrane Central Register of Controlled Trials 4](#_Toc198210093)

[Further details on data extraction and data synthesis 5](#_Toc198210094)

[Data Extraction 5](#_Toc198210095)

[Pairwise meta-analysis 5](#_Toc198210096)

[Network meta-analysis: consideration of effect modifiers 5](#_Toc198210097)

[Correlations of different C-peptide measures 6](#_Toc198210098)

[Network structure 7](#_Toc198210099)

[Ongoing Trials 8](#_Toc198210100)

[Risk of bias of included studies 12](#_Toc198210101)

[Studies contributing to each analysis 15](#_Toc198210102)

[Further results from main analysis 22](#_Toc198210103)

[Further results from secondary outcomes and sensitivity analyses 30](#_Toc198210104)

[Post-hoc analysis 47](#_Toc198210105)

[Adverse events 48](#_Toc198210106)

# Search Strategies

## Search strategy for MEDLINE database

Ovid MEDLINE(R) ALL <1946 to July 31, 2024>

1 diabetes mellitus, Type 1/

2 (T1D or TIDDM or T1DM).mp.

3 ((type 1 or type one or type I) adj2 diabet$).ti,ab.

4 ((newly diagnos$ or early onset or recent onset or sudden onset or childhood or young or teenager$) adj2 diabet$).ti,ab.

5 ((auto immune or auto-immune or autoimmune or insulin dependent) adj2 diabet$).mp.

6 or/1-5

7 randomized controlled trial.pt.

8 controlled clinical trial.pt.

9 randomized.ab.

10 randomised.ab.

11 placebo.ab.

12 clinical trials as topic.sh.

13 randomly.ab.

14 trial.ti.

15 or/7-14

16 6 and 15

17 exp animals/ not humans.sh.

18 16 not 17

## Search strategy for Embase database

Embase <1974 to 2024 July 31>

1 diabetes mellitus, Type 1/

2 insulin dependent diabetes mellitus/

3 (T1D or IDDM or T1DM).mp.

4 ((type 1 or type one or type I) adj2 diabet$).ti,ab.

5 ((newly diagnos$ or early onset or recent onset or sudden onset or acute onset or teenage$ or childhood or young) adj2 diabet$).ti,ab.

6 ((auto immune or auto-immune or autoimmmune or insulin dependent) adj2 diabet$).ti,ab.

7 or/1-6

8 controlled trial.ti,ab.

9 randomized controlled trial/

10 placebo$.ab.

11 double blind.ti,ab.

12 random$.ab.

13 single blind.ti,ab.

14 trial$.ti.

15 or/8-14

16 7 and 15

17 limit 16 to human

## Search strategy for Cochrane Central Register of Controlled Trials

ID Search Hits

#1 MeSH descriptor: [Diabetes Mellitus, Type 1] this term only

#2 (T1D or IDDM or T1DM):ti,ab

#3 (("type 1" or "type one" or "type I") near/2 diabet*):ti,ab

#4 ((newly NEXT diagnos* or "early onset" or "recent onset" or "sudden onset" or "acute onset" or teenage* or childhood or young) near/2 diabet*):ti,ab

#5 (("auto immune" or auto-immune or autoimmmune or "insulin dependent") near/2 diabet*):ti,ab

#6 #1 or #2 or #3 or #4 or #5

# Further details on data extraction and data synthesis

## Data Extraction

For each study, data were sought on: study design (including: participant allocation, methods of blinding, sample size; participations (including participant inclusion and exclusion criteria, methods of recruitment, participant characteristics, length of follow-up, completeness of follow-up and reasons for drop outs); intervention/comparator (including: type, mode of delivery, dose, duration adherence); outcomes (outcome, measurement method, time points; participant numbers at each time point; type of analysis (ITT/per protocol, data and associated uncertainty, statistical methods employed, imputation method of missing data). Detailed descriptions of how C-peptide was measured in each study including fasting/stimulated and method of stimulation, time points of measurement or duration of continuous measurement, units, and details on estimation of area under the curve measures). Where possible C-peptide data were converted to nmol/L. Authors of relevant abstracts were contacted for further information and data.

Data were taken from text or tables where possible, with data read from graphs as a secondary option using appropriate software. Means and standard deviations (or medians and IQR where data were skewed) were extracted for all outcomes, missing standard deviations were calculated from standard errors or confidence intervals where possible. Data were converted to means and standard deviations where possible and appropriate.

## Pairwise meta-analysis

Suitability for pairwise meta-analysis was assessed based on clinical and methodological homogeneity for each outcome (C-peptide, HbA1c, insulin dose), measurement method and type of data for each intervention-comparator dyad. Where meta-analysis was deemed appropriate the random effects model was used. Heterogeneity is reported using I^2^ and tau-squared statistics^16^ where appropriate. For each analysis, data were prioritised by type, (i) change scores from baseline for each group, calculated using an appropriate model (e.g. ANCOVA), (ii) end point data/final scores for each group at each reported time point during follow-up, (iii) change scores from baseline with no information on methods of calculation. Forest plots were created for each meta-analysis undertaken. Plots including all pooled estimates were created for each outcome. All pairwise meta-analyses were undertaken in RevMan.

Each type of C-peptide data (2-hour AUC, 4-hour AUC, maximum stimulation, fasting) and method of C-peptide stimulation (glucagon stimulated, meal stimulated, glucose stimulated or not stated) were reported separately in the systematic review and pairwise meta-analyses. Nmol/L was the preferred unit for reporting C-peptide. HbA1c was presented as percentages (%), and insulin dose as units/kg/day.

## Network meta-analysis: consideration of effect modifiers

To assess potential effect modifiers, we created bar charts and histograms showing weighted mean baseline C-peptide for each study included in the NMA. These ranged from 0.46 to 1.19. These studies did, however, include different types of C-peptide measurements (e.g. in terms of stimulation and point of measurement). Furthermore, we considered baseline imbalances in C-peptide as differences of >20% between intervention and comparator groups. Seven studies which were eligible for the NMA reported baseline imbalances above this threshold. These imbalances were addressed via a sensitivity analysis.

In terms of age, no studies included just children (0-11 years). The majority of the studies included a mixed population of children, adolescents and adults. N=11 and n=12 studies respectively included adults and adolescents, and children and adolescents.

For the trials eligible for the NMA we calculated correlation coefficients (and CIs) compatible with negligible/weak association in study-level aggregate data between sample-weighted mean age and weighted mean baseline C-peptide level (AUC 2hr/4hr where 2hr not reported), and between mean age and mean difference between therapy and placebo for C-peptide at 12 months follow-up. Such analyses indicated any variation in mean age across studies was acceptable for the main analyses, therefore subgroup analyses by age were not undertaken.

## Correlations of different C-peptide measures

Table 1. Table of correlation coefficients (r) between different types of c-peptide measurement reported in the literature where available. Blank cells indicate a lack of reported correlations

| Different measures of C-peptide and correlations between them | Meal stimulated Fasting | Meal stimulated 2H AUC | Meal stimulated 4H AUC | Glucagon stimulated 6 mins | Glucose stimulated | Meal stimulated peak | Glucagon stimulated fasting |
| --- | --- | --- | --- | --- | --- | --- | --- |
| Meal stimulated Fasting | X | 0.949^18^ |  | 0.875^18^ |  | 0.97^17^  (0.792^21^) | 0.913^18^ |
| Meal stimulated 2H AUC |  | X | 0.98^20^ | 0.936^18^ |  | 0.989^18^ |  |
| Meal stimulated 4H AUC |  |  | X |  |  |  |  |
| Glucagon stimulated 6 mins |  |  |  | X |  | 0.907^18^ | 0.988^19^ |
| Glucose stimulated |  |  |  |  | X |  |  |
| Meal stimulated peak |  |  |  |  |  | X |  |
| Glucagon Stimulated Fasting |  |  |  |  |  |  | X |

# Network structure

Figure S1. Network diagram. This figure demonstrates the structure of the network of interventions included in the main analysis. Each node represents an intervention in the network and each line demonstrates the available direct comparisons between pairs of interventions. As most interventions were compared to placebo/no treatment, the majority of the lines on the figure can be seen to be connected to placebo/no treatment. The numbers given on each line represent the number of trials contributing to a comparison.


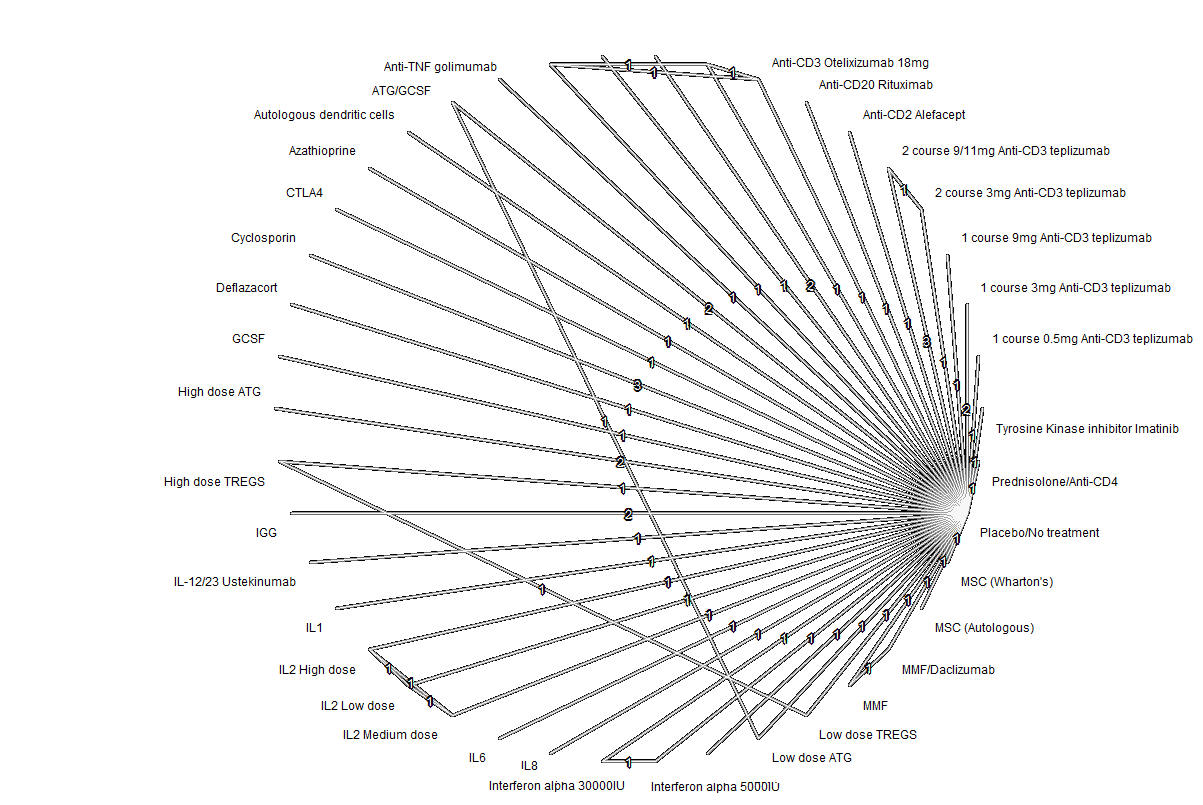


Anti-CD3 Otelixizumab 48mg

Anti-CD3 Otelixizumab 9mg

Anti-CD3 Otelixizumab 3mg

Anti-CD3 Otelixizumab 27mg

Interferon alpha

5000IU

JAK Inhibitor baricitinib

# Ongoing Trials

Table 2 Table of ongoing or unpublished trials in the non-antigen specific immunotherapy group

| **Trial Name/Identifier** | **Population** | **Intervention** | **Comparator** | **Status** | **Expected Completion Date (as per the trial record)** |
| --- | --- | --- | --- | --- | --- |
| NCT00071409 | Adults with history of onset of type 1 diabetes at or <20 years of age | 300 or 600mg daily subcutaneous injections of Islet Neogenesis Associated Protein (INGAP)-Peptide for 90 days | Placebo | Unpublished | Study completed in 2004 after recruiting 63 participants, no results published. |
| NCT00453375 | Adults diagnosed with type 1 diabetes with <5 years since diagnosis. | BHT-3021 intramuscular injections, up to 4 doses weekly for 12 weeks | Placebo | Unpublished | Study completed in 2011, no results published. |
| NCT00662519 | Over the age of 12 with confirmed diagnosis of type 1 diabetes for < 6 months | 12 week course of Neulasta (Pegylated granulocyte colony stimulating factor (GCSF)) subcutaneously every 2 weeks | Placebo | Unpublished | Study completed in 2014, no results published. |
| NCT00870818 | Those with recent-onset type 1 diabetes who were included in the Protégé study. This was an extension of the original trial. | Teplizumab, 3 different dosing groups | Placebo | Terminated | Study was terminated in 2011, no publications or further details given. |
| NCT00920582 | Children and adults with recent-onset type 1 diabetes. Protégé Encore study. | Teplizumab, various dosing groups | Placebo | Unpublished | Study completed in 2012, no publications or further information. |
| NCT01947569 | Patients with new onset T1DM aged 12 – 35 years | Dendritic cells | Placebo | Unpublished | No information, appears that no patients have been recruited |
| NCT02354911 | Patients with new onset T1DM aged 12 – 35 years | Dendritic cells | Placebo | Unpublished | No information, appears that no patients have been recruited |
| NCT01788033 | Patients with type 1 diabetes for at least 2 years | XOMA 052 | Placebo | Unpublished | Completed in 2013, no publications or further information |
| NCT02044848 | Type 1 diabetes patients aged 18-35 years within 100 days of diagnosis | Secukinumab | Placebo | Terminated | Study was terminated prematurely by the sponsor for business reasons |
| NCT02411253 | Children and adults with type 1 diabetes with no more than 3 months between insulin initiation and enrolment | Recombinant human interleukin-2 subcutaneous injections | Placebo | Ongoing | DIABIL-2 study 141 participants recruited, follow up ongoing. Estimated study completion date November 2022 |
| NCT02586831 | Adults with type 1 diabetes diagnosed for no longer than 120 days before randomisation | ATG, GCSF, interleukin-2, Etanercept, Exenatide combined in a regimen | Placebo for each respective intervention | Ongoing | Study ongoing with estimated completion in June 2025 |
| NCT03182426 | Adults with type 1 diabetes, duration less than 180 days | Immunological reset approach: Plerixafor, Alemtuzumab, Anakinra, Etanercept, Liraglutide | No treatment for first 12 months, followed by receiving the intervention from months 12 to 24 | Ongoing | Study ongoing, estimated completion data is December 2022 |
| NCT02691247 | Adolescents aged 8 to 17 years of age diagnosed with type 1 diabetes in <100 days | Autologous Ex Vivo Expanded Polyclonal Regulatory T-cells | Placebo | Completed | Study completed 2020, no results published as of yet |
| NCT03117998 | Adults with type 1 diabetes | REMD-477 (Volagidemab) | Placebo | Completed | Study recently completed in March 2021, results submitted but have not been quality control checked. No publication yet. |
| NCT02057211 | Adults with type 1 diabetes aged 18 – 40 years within 3 weeks of diagnosis | Mesenchymal stem cells | Placebo | Terminated | No results published |
| NCT04061746 | Adults with type 1 diabetes, newly diagnosed within the last 6 months | Mesenchymal Stromal Cells (MSC) | Placebo | Ongoing | March 2026 |
| NCT04129528 | Children and adults aged 6-21 with new onset type 1 diabetes | Iscalimab | Placebo | Ongoing | June 2027 |
| NCT04589325 | Adults diagnosed with type 1 diabetes within the previous 100 days | Ixekizumab | Placebo | Ongoing | December 2027 |
| NCT04509791 | Children and adults aged 5-25 years (starting with 12-25 then stepping down) within 6 weeks of type 1 diabetes diagnosis | ATG | Placebo | Ongoing | December 2024 |
| NCT04279613 | Adults with type 1 diabetes, with a duration of <48 months | NNC0361-0041 plasmid (Recombinant supercoiled plasmid encoding four human proteins: (pre-proinsulin (PPI), transforming growth factor β1 (TGF-β1), interleukin-10 (IL-10), and interleukin-2 (IL-2)) | Placebo | Ongoing | January 2024 |
| NCT04628481 | Patients with recent onset type 1 diabetes aged 14-45 (within 180 days from first insulin administration) | Ladarixin | Placebo | Ongoing | March 2025 |
| NCT05153070 | Patients with type 1 diabetes diagnosed within the previous 3 months, aged 16-45 | Cyclosporin and low dose IL2 | Placebo | Ongoing | July 2025 |
| NCT05018585 | Patients recently diagnosed with type 1 diabetes aged 12-28, within 6 months of screening | Recombinant human glutamic acid decarboxylase and Colecalciferol | Placebo | Ongoing | December 2025 |
| NCT04899271 | Newly diagnosed adults with type 1 diabetes, (within 100 days from first insulin administration) | Ladarixin | Placebo | Ongoing | December 2023 |
| NCT05061030 | Patients between 7-21 years of age that have recently (< 6 months) been diagnosed with type 1 diabetes | Mesenchymal Stromal Cells (MSC) | Placebo | Ongoing | December 2028 |
| NCT05281614 | Adults with a diagnosis of type 1 diabetes between 21 days and 3 years from screening | Vedolizumab and etanercept (anti-TNF) | Vedolizumab alone | Ongoing | April 2025 |
| Marcovecchio 2020: Interleukin-2 Therapy of Autoimmunity in Diabetes (ITAD) | Children and adolescents (6-18 years) initiated within 6 weeks of type 1 diabetes diagnosis | Ultra-low dose IL-2 (aldesleukin) | Placebo | Completed | Estimated completion September 2022, results not published yet |
| Wilhelm-Benartzi 2021: Minimum effective low dose: anti-human thymocyte globulin (MELD-ATG) | Participants 5-25 years diagnosed with type 1 diabetes within 3-9 weeks of planned treatment | ATG | Placebo | Ongoing | July 2024 |
| NCT05626712 | Patients with newly diagnosed type 1 diabetes aged 18 – 35 years, diagnosed within previous 180 days | CELZ-201 (Allostem) perinatal tissue derived cells | Standard care | Ongoing | January 2026 |
| NCT05742243/ ACTRN12622001236785 | Patients with newly diagnosed type 1 diabetes aged 6 – 21 years, diagnosed within last 100 days | Abatacept (CTLA4) and nasal insulin | Abatacept (CTLA4) and nasal placebo | Ongoing | February 2027 |
| NCT05594563 | Males and females 6- ≥40 years of age with a clinical diagnosis of T1D no more than 100 days prior to the time of randomization | Difluoromethylornithine (DFMO) | Placebo | Ongoing | December 2027 |
| NCT05574335 | Individuals aged 8-45 years with a Type 1 diabetes mellitus (T1DM) diagnosis. within 18 months of diagnosis | Siplizumab | 4 different doses of siplizumab compared | Ongoing | December 2027 |
| NCT05743244 | Subjects with recent onset Stage 3 Type 1 Diabetes within 100 days of diagnosis, aged 12 – 35 | Arm 1: Abrocitnib  Arm 2: Ritlecitinib | Placebo | Ongoing | June 2026 |
| CTRI/2022/08/044961 | Children with new-onset T1D | Hydroxychloroquine and imatinib | Placebo | Ongoing (not yet recruiting) | Unclear |
| NCT06111586 | Participants with newly diagnosed T1D on insulin treatment. | Frexalimab | Placebo | Ongoing | October 2028 |
| NCT03243058 | Participants diagnosed with T1D who would have had T1D from 4 months to 1 year at the time of randomization | IL-2 aldesleukin | Placebo | Ongoing | December 2028 |
| NL-OMON48794 | Participants diagnosed with T1D in the previous 8 weeks | Methylprednisolone and diazoxide | Placebo | Ongoing | Unclear |
| NCT06227221 | New-onset type 1 diabetes | Sorafenib | Placebo | Ongoing | December 2026 |
| ACTRN12623001089628 | Adaptive T1D patient arm, to evaluate the safety, tolerability, pharmacokinetics (PK) and pharmacodynamics (PD) of SAB-142 in healthy participants, and (if relevant) T1D patients. | Human biologic (SAB-142) | Placebo | Ongoing | December 2024 |
| NCT06455319 | Participants with newly diagnosed T1D identified as responders using an ex vivo predictive biomarker of response to ATG. | low-dose ATG | Placebo | Ongoing | Due to start April 2025 |
| ISRCTN45965456 | People with newly diagnosed type 1 diabetes | Golimumab or ATG as treatment arms | Verapamil as comparator arm | Ongoing | Recruitment underway and expected to conclude August 2025 |
| NCT06025110 | Adults recently diagnosed with type 1 diabetes | Siplizumab | Placebo | Ongoing | January 2025 |
| NCT06407297 | Paediatric patients diagnosed with newly onset type 1 diabetes | Allogeneic umbilical cord mesenchymal stem cells | Placebo | Ongoing | May 2026 |
| ***Abbreviations:*** Anti-thymocyte globulin; GCSF= Granulocyte colony-stimulating factor; JAK=janus kinase inhibitor | | | | | |
|  | | | | | |

# Risk of bias of included studies

Table 3. Risk of bias assessment results for included studies as assessed with Cochrane Risk of Bias tool version 1

| **Study ID** | **Random sequence generation** | **Allocation concealment** | **Blinding of patients** | **Blinding of  C-peptide outcome** | **Incomplete outcome data C-peptide outcome** | **Selective reporting** |
| --- | --- | --- | --- | --- | --- | --- |
| Herold 2005 | U | U | H | U/L | U/L | L |
| Keymeulen 2005 | U | L | L | L | L | L |
| Herold 2009 | U | U | L | U | L | U |
| Herold 2002 | L | U | H | H | L | U |
| Sherry 2011 | L | L | L | L | L | U |
| Ambery 2014 | U | U | U/L | U | L | U |
| Herold 2013b | U | U | L | U | L | U |
| Herold 2013a | L | L | L | U | U | U |
| Aronson 2014 | U | U | U/L | L | L | U/L |
| Keymeulen 2021 | L | L | L | L | U | L |
| Vague 1989 | L | U | L | L | L | H |
| Chase 1990 | L | L | U | U | H | U |
| Martin 1991 | U | H | L | L | L | U |
| Skyler 1992 | L | U | L | U | L | L |
| Pozzilli 1994 | L | U | H | L | H | U |
| Rigby 2013 | L | L | L | H | L | L |
| Zielinski 2022 | L | L | L | U | L | L |
| Pescovitz 2009 | U | U | L | U | L | L |
| Orban 2011 | L | L | L | U | U | L |
| Saudek 2004 | L | U | L | U | U | U |
| Gitelman 2013 | L | L | L | U | L | L |
| Haller 2018 | L | L | L | U | L | L |
| Haller 2014a | U | U | U | U | U | U |
| Haller 2014b | U | U | L | U | U | L |
| Hessner 2013 | U | U | L | U | U | U |
| Moran 2013a | L | L | L | U | L | U |
| Moran 2013b | L | L | L | U | L | U |
| Hu 2013 | U | L | L | U | L | L |
| Carlsson 2015 | U | U | U | U | L | U |
| Izadi 2022 | L | L | L | L | L | L |
| Silverstein 1988 | U | U | U | U | U | U |
| Harrison 1985 | H | U | U | U | L | U |
| Cook 1989 | U | U | L | U | L | U |
| Panto 1990 | U | U | U | U | L | U |
| Lorini 1991 | U | U | U | U | L | U |
| Colagiuri 1996 | U | U | U | U | H | H |
| Rother 2009 | L | L | L | U | H | L |
| Gottlieb 2010 | U | U | L | U | L | L |
| Secchi 1990 | U | U | U | U | U | U |
| Hehmke 1994 | U | U | U | U | L | U |
| Pozzilli 1994 (deflaz) | U | U | L | L | L | U |
| Buckingham 2000 | L | L | H | H | L | H |
| Ludvigsson 2001 | U | U | L | U | L | U |
| Mastrandrea 2009 | U | U | L | L | L | L |
| Cabrera-Rode 2022 | L | U | L | L | U | H |
| Curtin 2020 | U | U | L | L | L | L |
| Quattrin 2020 | L | L | L | L | L | L |
| Gitelman 2021 | L | L | L | L | L | L |
| Greenbaum 2021 | L | L | L | L | L | L |
| Piemonti 2022 | L | L | L | L | L | L |
| Rosenzwajg 2020 | L | L | L | L | L | L |
| Von Herrath 2021 | L | L | L | L | L | L |
| Carlsson 2023 | L | L | L | U | L | L |
| Bender 2024 | L | L | L | L | H | L |
| Waibel 2023 | L | L | L | L | L | L |
| Ramos 2023 | L | L | L | L | L | L |
| Mathieu 2024 | L | L | L | L | L | L |
| Gaglia 2024 | L | L | L | L | L | L |
| Chujo 2023 | U | U | H | U | L | U |
| Tatovic 2024 | L | L | L | L | L | L |

# Studies contributing to each analysis

Table 4. Studies which contributed to each network meta-analysis undertaken at 12 months

This table displays which of the included trials in the systematic review were eligible to be included in each network meta-analysis undertaken

| **Contributing data to each analysis at 12 Months follow up** | | | | | | | | | | |
| --- | --- | --- | --- | --- | --- | --- | --- | --- | --- | --- |
| **Sub-Group** | **Study ID** | **HbA1c** | **Insulin dose** | **All C-peptide Data Main Analysis (2H AUC data used if both 2/4 reported)** | **Low risk of bias trials only** | **Removal of studies with baseline c-peptide imbalances** | **Mean change data only** | **Endpoint data only** | **All C-pep Data but with 4H AUC data instead of 2H** | **2H/4H AUC trials ONLY but with 2H data prioritised** |
| Anti-CD3 - Tep vs no Tep | Herold 2005 |  |  | ✓ |  | ✓ |  | ✓ | ✓ | ✓ |
| Anti-CD3 – Tep vs no Tep | Herold 2002 | ✓ | ✓ | ✓ |  | ✓ |  | ✓ | ✓ | ✓ |
| Anti-CD3 - Tep vs no Tep | Herold 2009 | ✓ | ✓ | ✓ |  |  |  | ✓ | ✓ | ✓ |
| Anti-CD3 - Tep vs placebo | Sherry 2011 | ✓ | ✓ | ✓ | ✓ | ✓ | ✓ |  | ✓ | ✓ |
| Anti-CD3 - Tep  vs placebo | Herold 2013 a | ✓ | ✓ | ✓ | ✓ | ✓ |  | ✓ | ✓ | ✓ |
| Anti-CD3 - Tep vs no Tep | Herold 2013 b | Certainty data not clear | Not reported at 12m | ✓ |  | ✓ |  | ✓ | ✓ | ✓ |
| Anti-CD3 - Tep  vs placebo | Ramos 2023 | ✓ | ✓ | ✓ | ✓ | ✓ |  | ✓ | ✓ | ✓ |
| Anti-CD3 – Tep + AG019 vs AG019 + placebo | Mathieu 2024 |  |  | No placebo data at 12m |  |  |  |  |  |  |
| Anti-CD3 - Otel vs placebo | Keymeulen 2005 | ✓ | ✓ | ✓ | ✓ | ✓ |  | ✓ | ✓ |  |
| Anti-CD3 - Otel vs placebo | Ambery 2014 |  |  | ✓ |  | ✓ | ✓ | ✓ | ✓ | ✓ |
| Anti-CD3 - Otel vs placebo | Aronson 2014 | ✓ | ✓ | ✓ |  | ✓ | ✓ | ✓ | ✓ | ✓ |
| Anti-CD3 - Otel vs placebo | Keymeulen 2021 | Measured, data not reported | Measured, data not reported | ✓ | ✓ |  | ✓ |  | ✓ | ✓ |
| Cyclosporin  vs placebo | Vague 1989 | ✓ | Data incorrect format | ✓ |  | ✓ |  | ✓ | ✓ |  |
| Cyclosporin vs no cyclosporin | Chase 1990 |  |  | Data incorrect format |  |  |  |  |  |  |
| Cyclosporin vs placebo | Martin 1991 | ✓ | **✓** | ✓ |  | ✓ | ✓ | ✓ | ✓ |  |
| Cyclosporin vs placebo | Skyler 1992 | ✓ | ✓ | ✓ |  | ✓ |  | ✓ | ✓ | ✓ |
| Cyclosporin + Nicotinamide vs insulin only | Pozzilli 1994 |  |  | No data at 12m |  |  |  |  |  |  |
| Anti-CD2 - Alefacept vs placebo | Rigby 2013 | ✓ | ✓ | ✓ | ✓ |  | ✓ |  | ✓ | ✓ |
| B Cell - Rituximab vs placebo | Pescovitz 2009 | ✓ | ✓ | ✓ |  | ✓ |  | ✓ | ✓ | ✓ |
| Regulatory T Cell/B cell - Rituximab and Tregs vs placebo and Tregs | Zielinski 2022 |  |  | No placebo or common comparator so could not be included in network |  |  |  |  |  |  |
| Polyclonal autologous Tregs vs placebo | Bender 2024 | ✓ | ✓ | ✓ | ✓ | ✓ |  | ✓ | ✓ | ✓ |
| CTLA-4 vs placebo | Orban 2011 | ✓ | ✓ | ✓ | ✓ | ✓ |  | ✓ | ✓ | ✓ |
| ATG vs placebo | Saudek 2004 | ✓ | ✓ | ✓ |  |  |  | ✓ | ✓ |  |
| ATG vs placebo | Gitelman 2013 | Median/95% CI only | Median/95% CI only | ✓ | ✓ | ✓ | ✓ | ✓ | ✓ | ✓ |
| ATG/PEG vs placebo | Chujo 2023 |  |  | Data incorrect format (conference abstract only) |  |  |  |  |  |  |
| ATG/GCSF vs placebo | Haller 2014b | ✓ | ✓ | ✓ |  | ✓ |  | ✓ | ✓ | ✓ |
| ATG/GCSF vs placebo | Haller 2018 | ✓ | ✓ | ✓ | ✓ | ✓ |  | ✓ | ✓ | ✓ |
| GCSF vs placebo | Haller 2014a | ✓ | ✓ | ✓ |  |  | ✓ |  | ✓ | ✓ |
| IL-1 vs placebo | Hessner 2013 |  |  | Nine-month data only |  |  |  |  |  |  |
| IL-1 vs placebo | Moran 2013a | ✓ | ✓ | ✓ | ✓ | ✓ |  | ✓ | ✓ | ✓ |
| IL-1 vs placebo | Moran 2013b |  |  | Nine-month data only |  |  |  |  |  |  |
| MSC  vs placebo | Hu 2013 | ✓ | No certainty data reported | ✓ | ✓ | ✓ |  | ✓ | ✓ |  |
| MSC vs insulin only | Carlsson 2015 | ✓ | ✓ | ✓ |  | ✓ | ✓ |  | ✓ |  |
| MSC vs placebo | Carlsson 2023 | Report medians, data cannot be transformed | Report medians, data cannot be transformed | Report medians, data cannot be transformed |  |  |  |  |  |  |
| MSC vs placebo | Izadi 2022 |  |  | Not enough information about C-pep measurement |  |  |  |  |  |  |
| ADC vs placebo | Gaglia 2024 | ✓ | ✓ | ✓ |  | ✓ | ✓ |  | ✓ | ✓ |
| Azathioprine vs no aza | Silverstein 1988 |  |  | Not enough information about C-pep measurement |  |  |  |  |  |  |
| Azathioprine  vs placebo | Harrison 1985 | NR | Data in incorrect format | ✓ |  |  |  | ✓ | ✓ |  |
| Azathioprine vs no aza | Cook 1989 |  |  | Medians only reported |  |  |  |  |  |  |
| Immunoglobulin vs no immunoglobulin | Panto 1990 |  |  | No 12-month data |  |  |  |  |  |  |
| Immnuoglobulin vs insulin only | Lorini 1991 | ✓ | ✓ | ✓ |  | ✓ |  | ✓ | ✓ |  |
| Immunoglobulin vs placebo | Colagiuri 1996 | ✓ | ✓ | ✓ |  |  |  | ✓ | ✓ |  |
| Interferon α vs placebo | Rother 2009 | ✓ | ✓ | ✓ | ✓ | ✓ |  | ✓ | ✓ | ✓ |
| Daclizumab Placebo | Gottlieb 2010 | ✓ | ✓ | ✓ |  | ✓ |  | ✓ | ✓ | ✓ |
| 1: Prednisolone 2: Indomethacin vs placebo | Secchi 1990 |  |  | Urinary c-peptide only |  |  |  |  |  |  |
| Anti-CD4 + Prednisolone vs placebo | Hehmke 1994 | ✓ | ✓ | ✓ |  |  |  | ✓ | ✓ |  |
| Others Deflazacort and nicotinamide vs nicotinamide alone | Pozzilli 1994 | ✓ | ✓ | ✓ |  |  |  | ✓ | ✓ |  |
| Others Methotrexate vs no methotrexate | Buckingham 2000 |  |  | Three-month data only |  |  |  |  |  |  |
| Others Photophoresis vs placebo | Ludvigsson 2001 |  |  | No certainty data reported |  |  |  |  |  |  |
| Others Etanercept vs placebo | Mastrandrea 2009 |  |  | Six-month data only |  |  |  |  |  |  |
| Other  humanized IgG1 monoclonal antibody (Itolizumab) vs placebo | Cabrera-Rode 2022 |  |  | Full data not reported, no response from authors |  |  |  |  |  |  |
| Other immunoglobulin (Ig) G4 monoclonal antibody (Temelimab) vs placebo | Curtin 2020 |  |  | Six-month data only |  |  |  |  |  |  |
| Anti-TNF Golimumab vs placebo | Quattrin 2020 | ✓ | ✓ | ✓ | ✓ | ✓ | ✓ | ✓ | ✓ | ✓ |
| Anti-receptor tyrosine kinases Imatinib | Gitelman 2021 | ✓ | ✓ | ✓ | ✓ | ✓ |  | ✓ | ✓ | ✓ |
| JAK inhibitor Baricitinib vs placebo | Waibel 2023 | ✓ | ✓ | ✓ | ✓ | ✓ |  | ✓ | ✓ | ✓ |
| Anti-IL6 Receptor Toclizumab vs placbo | Greenbaum 2021 | ✓ | ✓ | ✓ | ✓ |  |  | ✓ | ✓ | ✓ |
| Anti IL-8 Receptor Ladarixin vs placebo | Piemonti 2022 | ✓ | ✓ | ✓ | ✓ | ✓ |  | ✓ | ✓ | ✓ |
| Regulatory T-cell Low dose IL-2 vs placebo | Rosenzwajg 2020 | ✓ | ✓ | ✓ | ✓ |  | ✓ | ✓ | ✓ | ✓ |
| Anti IL-21 Receptor vs placebo | Von Herrath 2021 |  |  | No certainty data |  |  |  |  |  |  |
| IgG1k Ustekinumab vs placebo | Tatovic 2024 | ✓ | ✓ | ✓ | ✓ | ✓ |  | ✓ | ✓ | ✓ |
| *Secondary outcomes HbA1c and insulin dose were only considered in trials that also contributed to the c-peptide analysis.* | | | | | | | | | | |

#
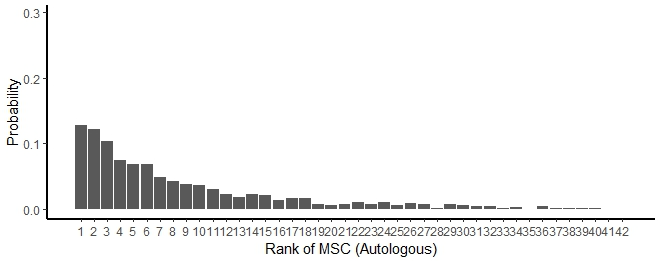

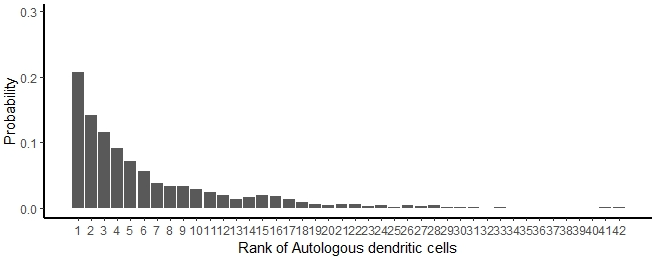

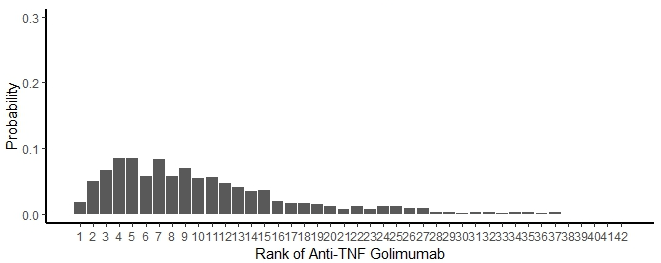

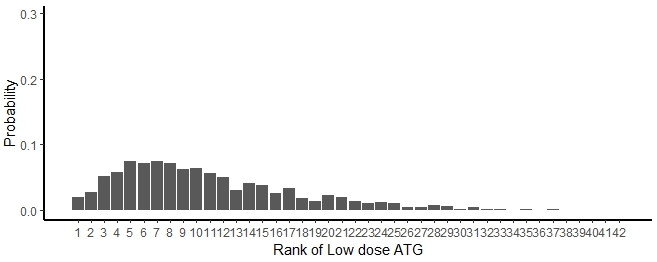

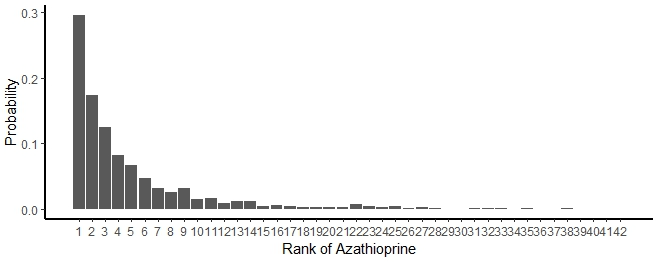

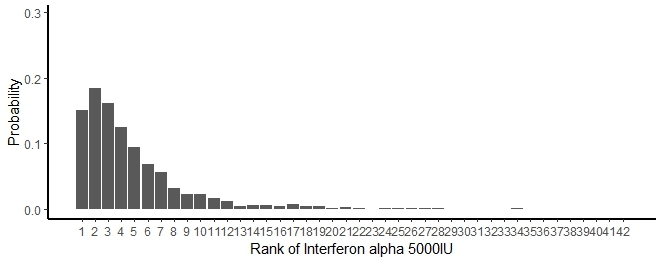
Further results from main analysis


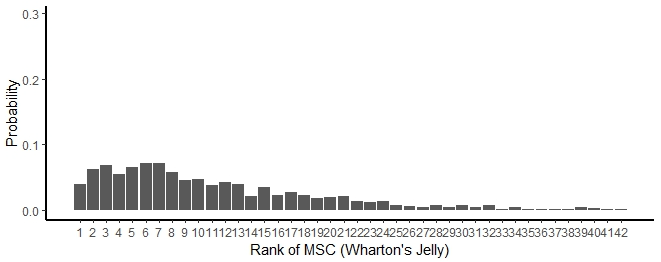

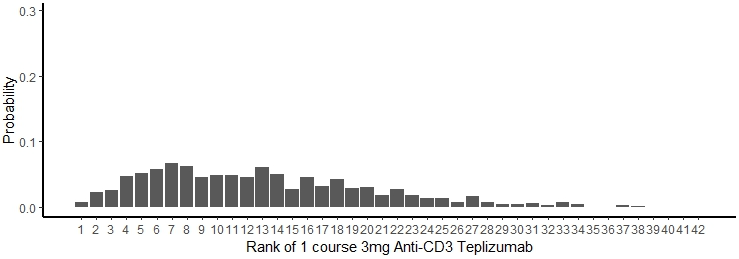


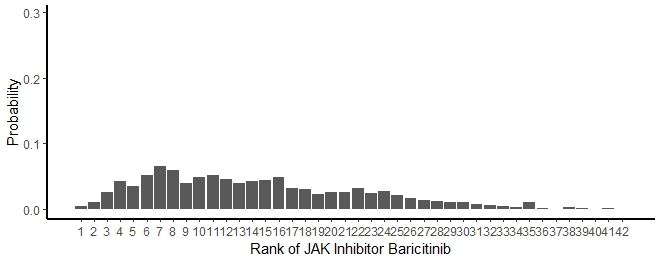

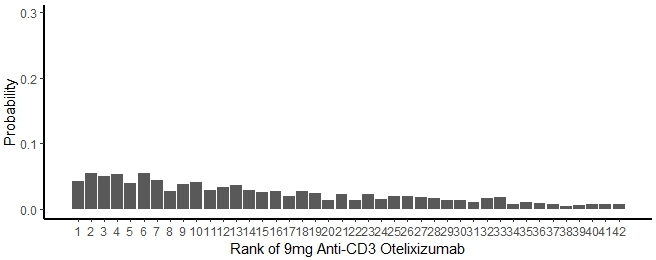


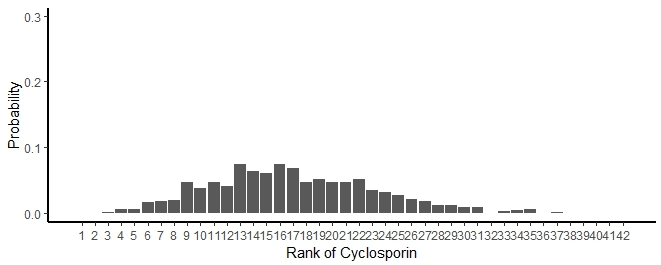

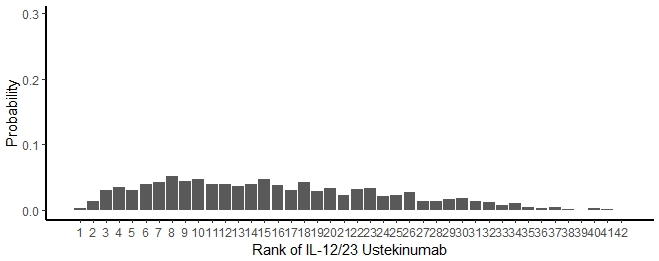


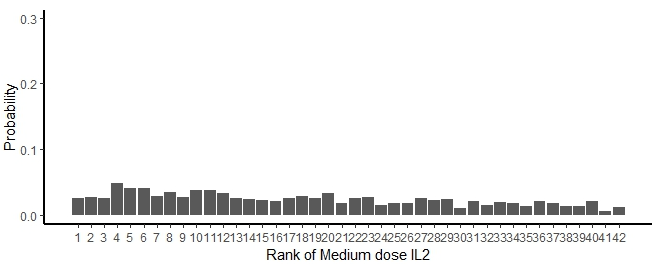

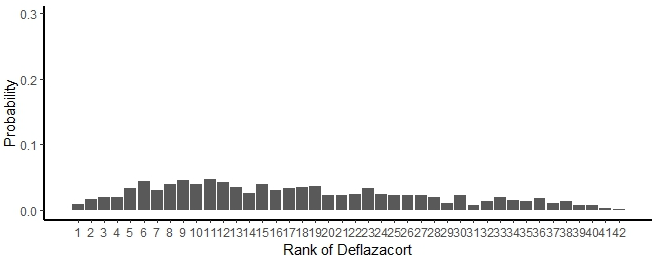


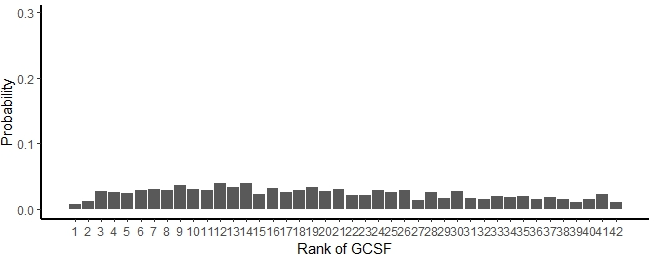

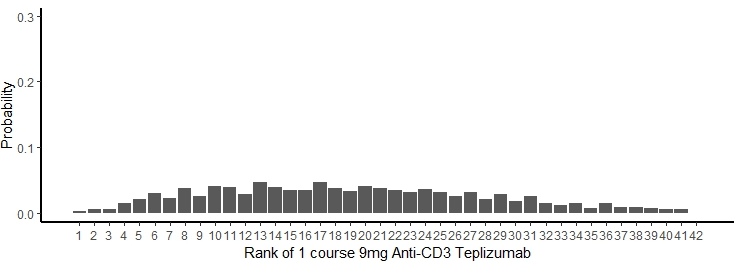

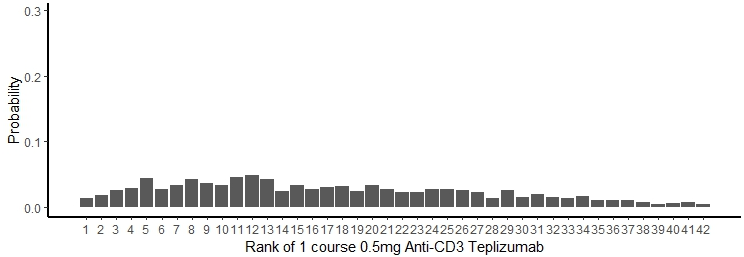


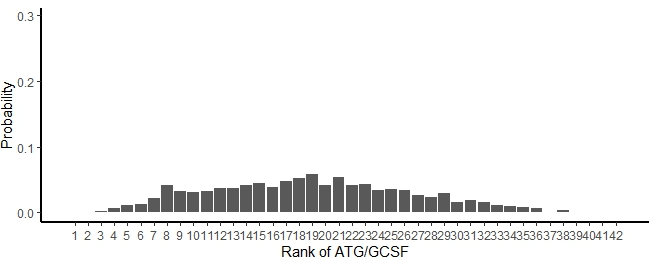


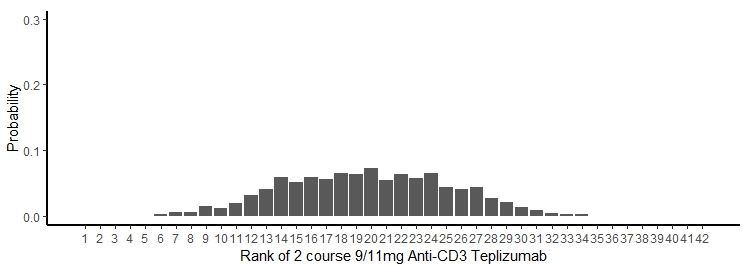

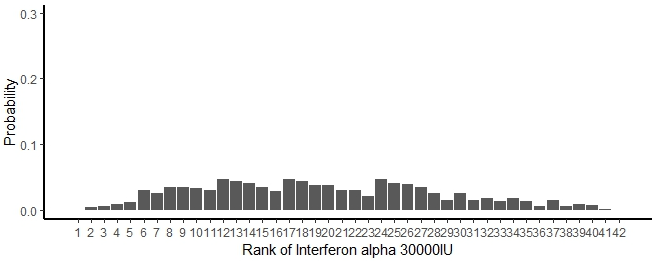


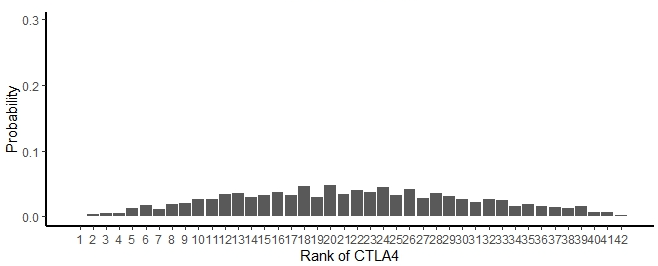

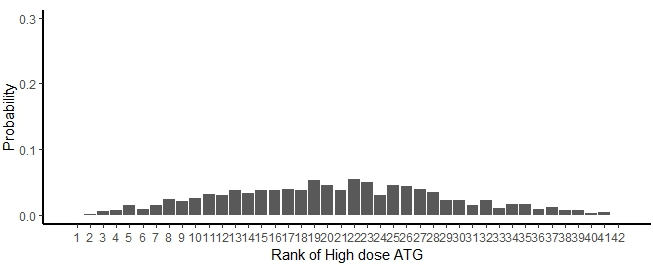

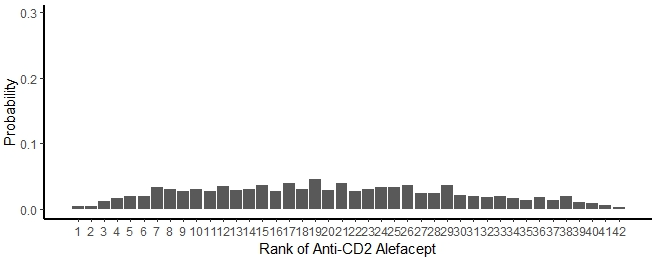


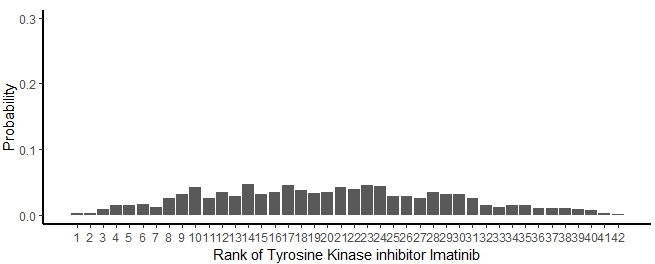


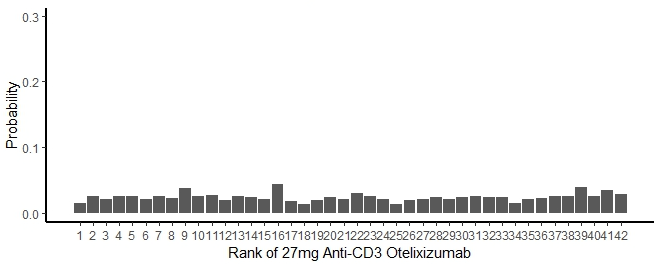

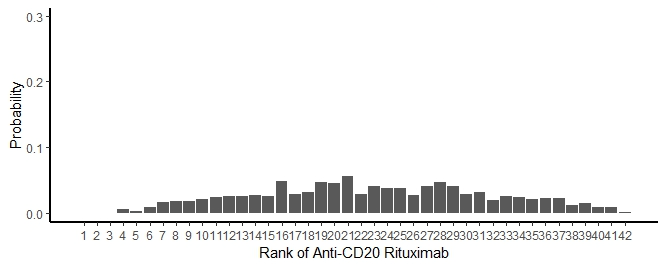


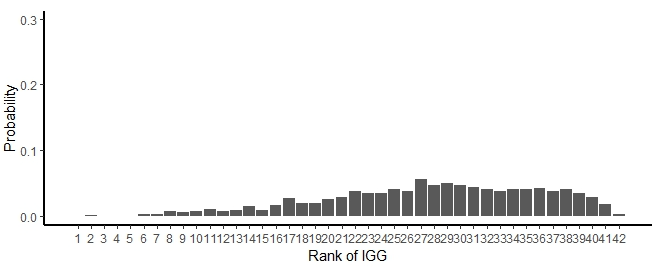

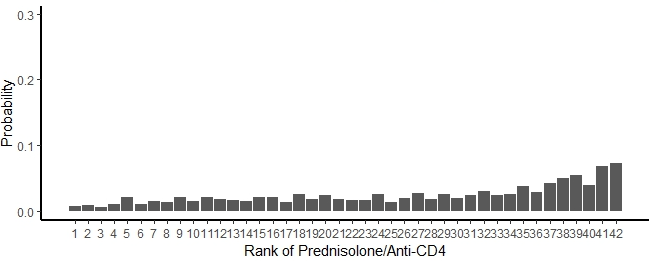

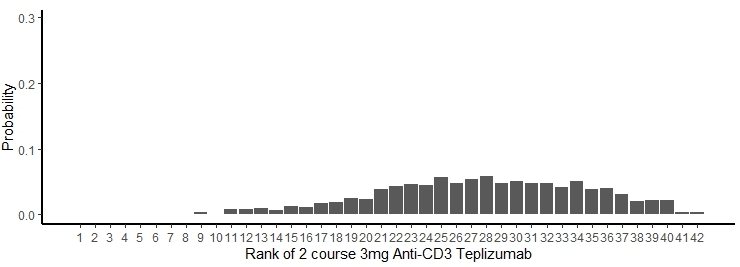

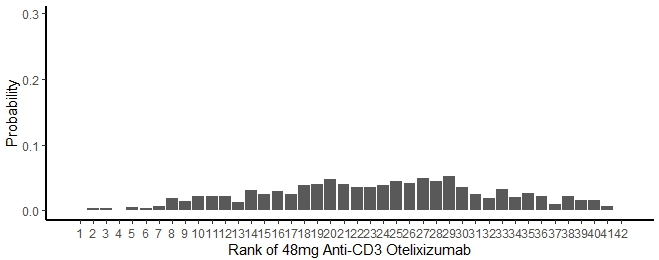


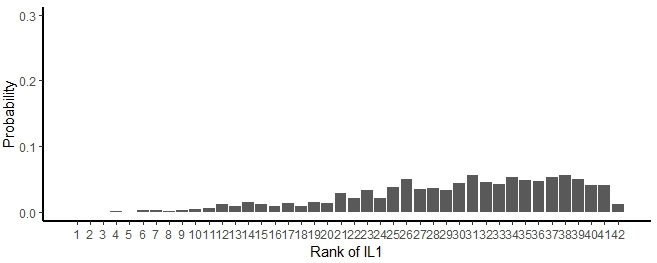

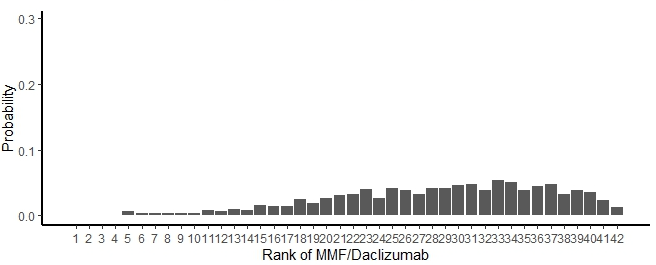


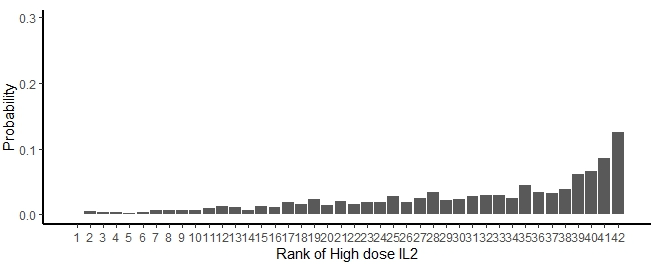

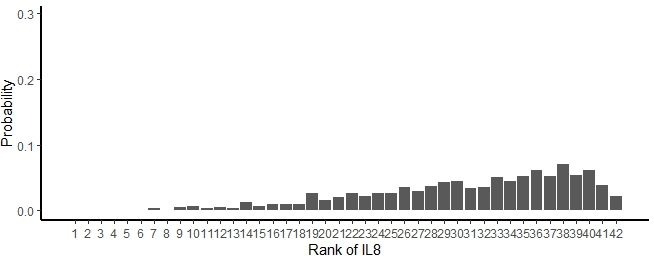


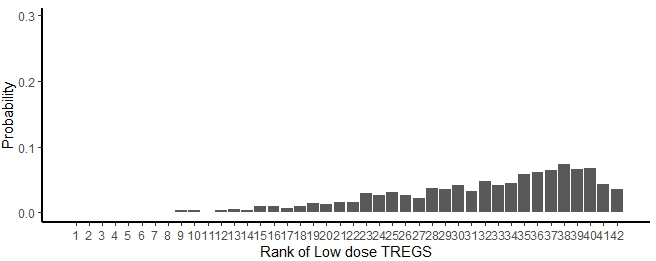

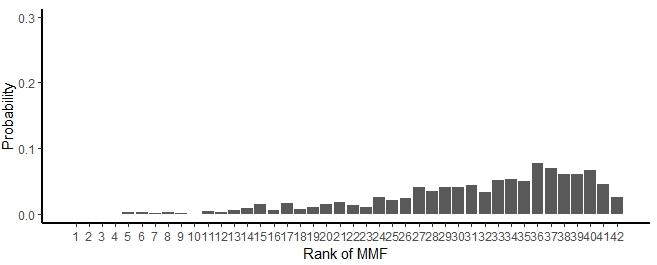


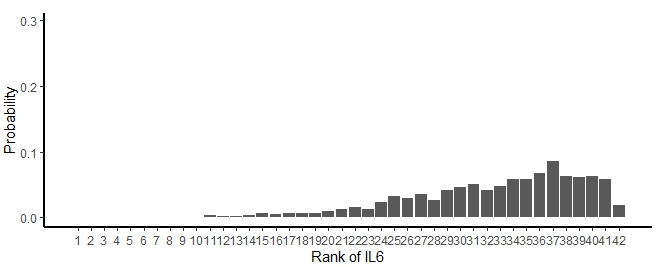

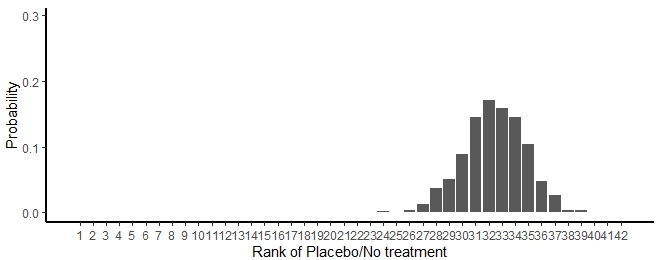


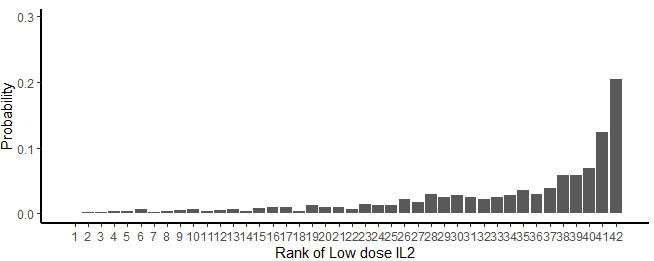

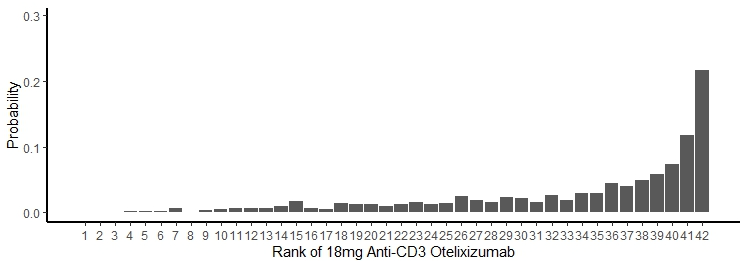


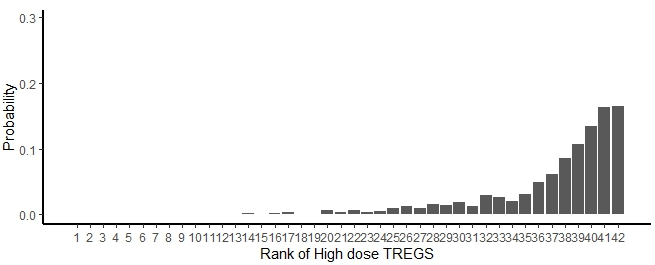

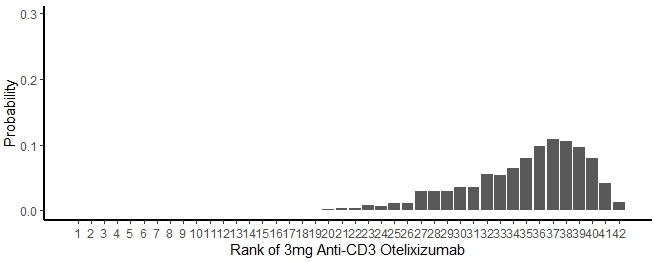


Figure S2. Non-cumulative ranking curves for each intervention included in the main analysis. The above figures display the ranking probabilities for each intervention included in the main analysis. Each curve reports the probabilities of one intervention being ranked at each possible ranking position. The interventions are ordered by highest probability of ranking first.

# Further results from secondary outcomes and sensitivity analyses

| **Intervention** | **Median Rank** | **95% Confidence interval** |
| --- | --- | --- |
| Autologous dendritic cells | 3 | 1 – 14 |
| Interferon alpha 5000IU | 3 | 1 – 12 |
| Anti-TNF Golimumab | 7 | 1 – 19 |
| MSC (Wharton's Jelly) | 7 | 1 – 22 |
| Low dose ATG | 8 | 2 – 20 |
| 9mg Anti-CD3 Otelixizumab | 9 | 1 – 26 |
| JAK Inhibitor Baricitinib | 9 | 2 – 22 |
| IL-12/23 Ustekinumab | 10 | 2 – 23 |
| Medium dose IL2 | 10 | 2 – 27 |
| 1 course 9mg Anti-CD3 Teplizumab | 13 | 3 – 25 |
| Interferon alpha 30000IU | 13 | 3 – 25 |
| 2 course 9/11mg Anti-CD3 Teplizumab | 14 | 6 – 23 |
| Anti-CD2 Alefacept | 14 | 3 – 26 |
| ATG/GCSF | 14 | 4 – 26 |
| Tyrosine Kinase inhibitor Imatinib | 14 | 3 – 25 |
| 27mg Anti-CD3 Otelixizumab | 15 | 2 – 28 |
| CTLA4 | 15 | 4 – 26 |
| 48mg Anti-CD3 Otelixizumab | 16 | 5 – 27 |
| 2 course 3mg Anti-CD3 Teplizumab | 19 | 8 – 27 |
| High dose ATG | 19 | 6 – 28 |
| IL1 | 21 | 7 – 28 |
| IL8 | 21 | 7 – 29 |
| Placebo/No treatment | 21 | 17 – 25 |
| Low dose TREGS | 22 | 9 – 28 |
| High dose IL2 | 23 | 6 – 29 |
| IL6 | 23 | 9 – 29 |
| 18mg Anti-CD3 Otelixizumab | 25 | 7 – 29 |
| Low dose IL2 | 25 | 9 – 29 |
| High dose TREGS | 26 | 14 – 29 |

Table 5. Median rankings and 95% confidence intervals for each intervention in the low risk of bias subset of trials from the main analysis

Figure S3. Forest plot displaying results of network meta-analysis of daily insulin doses (units/kg/day) in therapies included in the main C-peptide analysis versus placebo or no treatment.


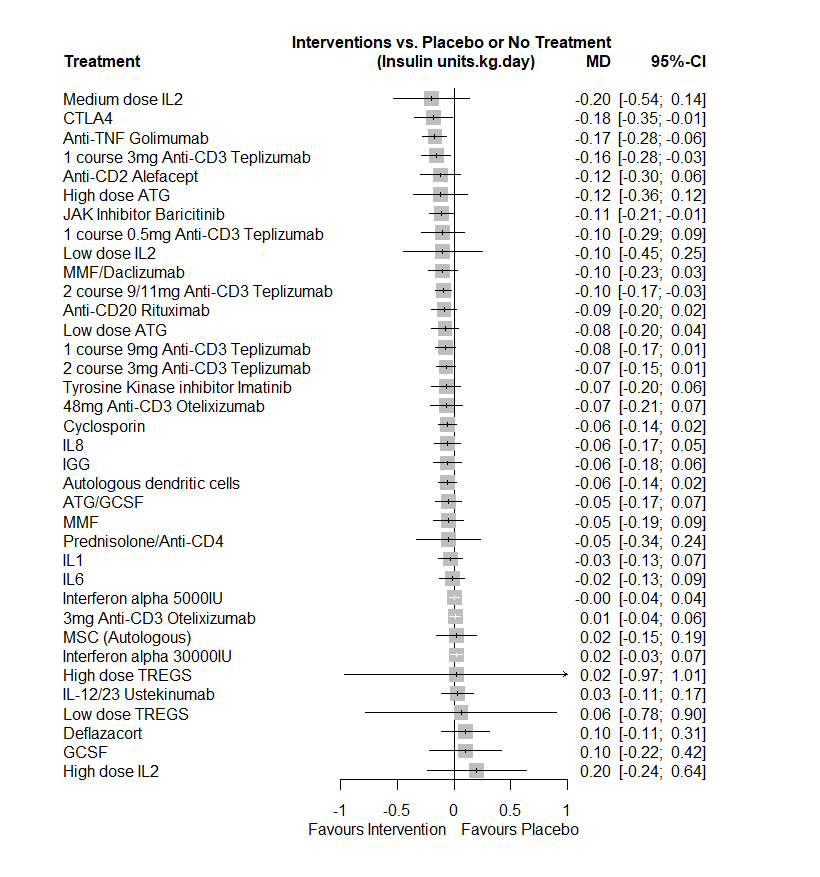


*Outcome metric is mean difference (and 95% confidence intervals). Summary estimates for each intervention versus comparator are listed. Effect estimates to the left of the line of no effect demonstrate a reduction in daily insulin dose compared to placebo/no comparator.*

Table 6. Median rankings and 95% confidence intervals for each intervention in the insulin dose outcome

| **Intervention** | **Median Rank** | **95% Confidence interval** |
| --- | --- | --- |
| Medium dose IL2 | 4 | 1 – 33 |
| Anti-TNF Golimumab | 5 | 2 – 15 |
| CTLA4 | 5 | 1 – 21 |
| 1 course 3mg Anti-CD3 Teplizumab | 6 | 2 – 19 |
| Anti-CD2 Alefacept | 10 | 2 – 31 |
| High dose ATG | 10 | 1 – 33 |
| JAK Inhibitor Baricitinib | 11 | 3 – 24 |
| 1 course 0.5mg Anti-CD3 Teplizumab | 13 | 2 – 33 |
| 2 course 9/11mg Anti-CD3 Teplizumab | 13 | 6 – 22 |
| Anti-CD20 Rituximab | 13 | 4 – 27 |
| Low dose IL2 | 13 | 1 – 35 |
| MMF/Daclizumab | 13 | 3 – 29 |
| 1 course 9mg Anti-CD3 Teplizumab | 16 | 6 – 27 |
| 48mg Anti-CD3 Otelixizumab | 16 | 5 – 32 |
| Low dose ATG | 16 | 5 – 30 |
| Tyrosine Kinase inhibitor Imatinib | 16 | 5 – 32 |
| 2 course 3mg Anti-CD3 Teplizumab | 17 | 7 – 27 |
| Cyclosporin | 18 | 8 – 28 |
| IL8 | 18 | 6 – 32 |
| ATG/GCSF | 19 | 6 – 32 |
| Autologous dendritic cells | 19 | 9 – 30 |
| IGG | 19 | 6 – 32 |
| Prednisolone/Anti-CD4 | 19 | 2 – 36 |
| MMF | 20 | 6 – 33 |
| IL1 | 23 | 10 – 33 |
| IL6 | 24 | 10 – 34 |
| Interferon alpha 5000IU | 27 | 20 – 32 |
| Placebo/No treatment | 27 | 22 – 31 |
| 3mg Anti-CD3 Otelixizumab | 28 | 21 – 33 |
| Interferon alpha 30000IU | 29 | 22 – 34 |
| MSC (Autologous) | 29 | 9 – 36 |
| IL-12/23 Ustekinumab | 30 | 15 – 35 |
| High dose TREGS | 33 | 1 – 27 |
| Deflazacort | 34 | 14 – 37 |
| GCSF | 34 | 6 – 27 |
| Low dose TREGS | 34 | 1 – 37 |
| High dose IL2 | 35 | 7 – 27 |

Figure S4. Forest plot displaying results of network meta-analysis of HbA1c levels (%) in therapies included in the main C-peptide analysis versus placebo or no treatment


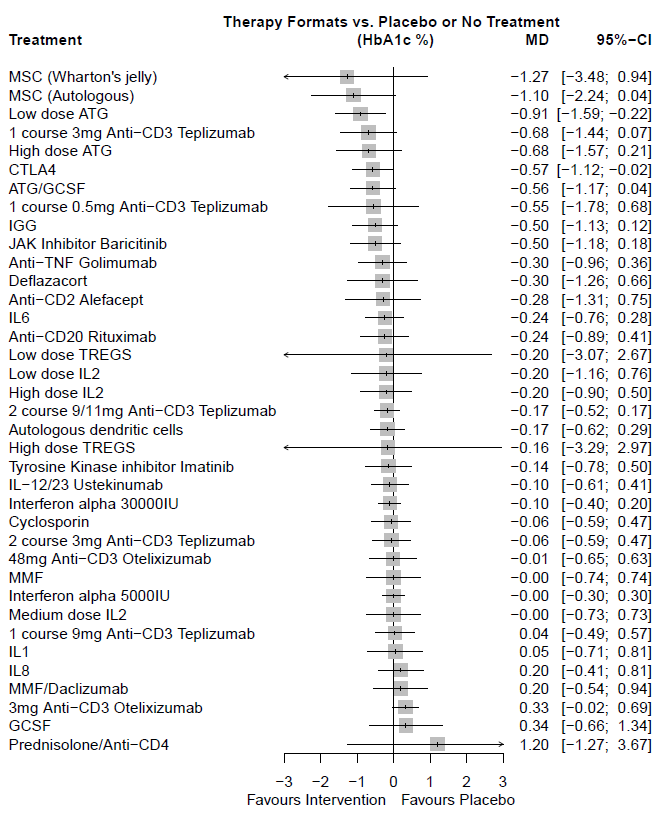


*Outcome metric is mean difference (and 95% confidence intervals). Summary estimates for each intervention versus comparator are listed. Effect estimates to the left of the line of no effect demonstrate a reduction in HbA1c compared to placebo/no comparator.*

Table 7. Median rankings and 95% confidence intervals for each intervention in the HbA1c outcome

| **Intervention** | **Median Rank** | **95% Confidence interval** |
| --- | --- | --- |
| MSC (Autologous) | 3 | 1 – 19 |
| MSC (Wharton's jelly) | 3 | 1 – 35 |
| Low dose ATG | 5 | 2 – 14 |
| 1 course 3mg Anti-CD3 Teplizumab | 8 | 2 – 24 |
| High dose ATG | 8 | 2 – 28 |
| 1 course 0.5mg Anti-CD3 Teplizumab | 10 | 2 – 35 |
| ATG/GCSF | 10 | 3 – 24 |
| CTLA4 | 10 | 3 – 22 |
| JAK Inhibitor Baricitinib | 10 | 3 – 27 |
| IGG | 11 | 3 – 26 |
| Anti-TNF Golimumab | 16 | 5 – 32 |
| Deflazacort | 16 | 3 – 36 |
| Anti-CD2 Alefacept | 17 | 3 – 36 |
| Anti-CD20 Rituximab | 17 | 6 – 34 |
| Low dose TREGS | 17 | 1 – 38 |
| High dose IL2 | 18 | 6 – 34 |
| IL6 | 18 | 7 – 31 |
| Low dose IL2 | 18 | 4 – 36 |
| 2 course 9/11mg Anti-CD3 Teplizumab | 19 | 11 – 29 |
| Autologous dendritic cells | 19 | 9 – 31 |
| High dose TREGS | 19 | 1 – 38 |
| Tyrosine Kinase inhibitor Imatinib | 21 | 7 – 34 |
| IL-12/23 Ustekinumab | 22 | 9 – 33 |
| Interferon alpha 30000IU | 22 | 13 – 31 |
| 2 course 3mg Anti-CD3 Teplizumab | 23 | 10 – 34 |
| Cyclosporin | 23 | 10 – 34 |
| 48mg Anti-CD3 Otelixizumab | 25 | 9 – 36 |
| Interferon alpha 5000IU | 25 | 16 – 33 |
| Medium dose IL2 | 25 | 8 – 36 |
| Placebo/No treatment | 25 | 21 – 30 |
| IL1 | 26 | 9 – 36 |
| MMF | 26 | 9 – 36 |
| 1 course 9mg Anti-CD3 Teplizumab | 27 | 12 – 35 |
| MMF/Daclizumab | 30 | 13 – 37 |
| IL8 | 31 | 15 – 37 |
| 3mg Anti-CD3 Otelixizumab | 33 | 26 – 37 |
| GCSF | 33 | 11 – 38 |
| Prednisolone/Anti-CD4 | 37 | 5 – 38 |

Figure S5. Forest plot displaying results of network meta-analysis of HbA1c levels (%) in a subset of therapies which were considered low risk of bias from trials in the main analysis.


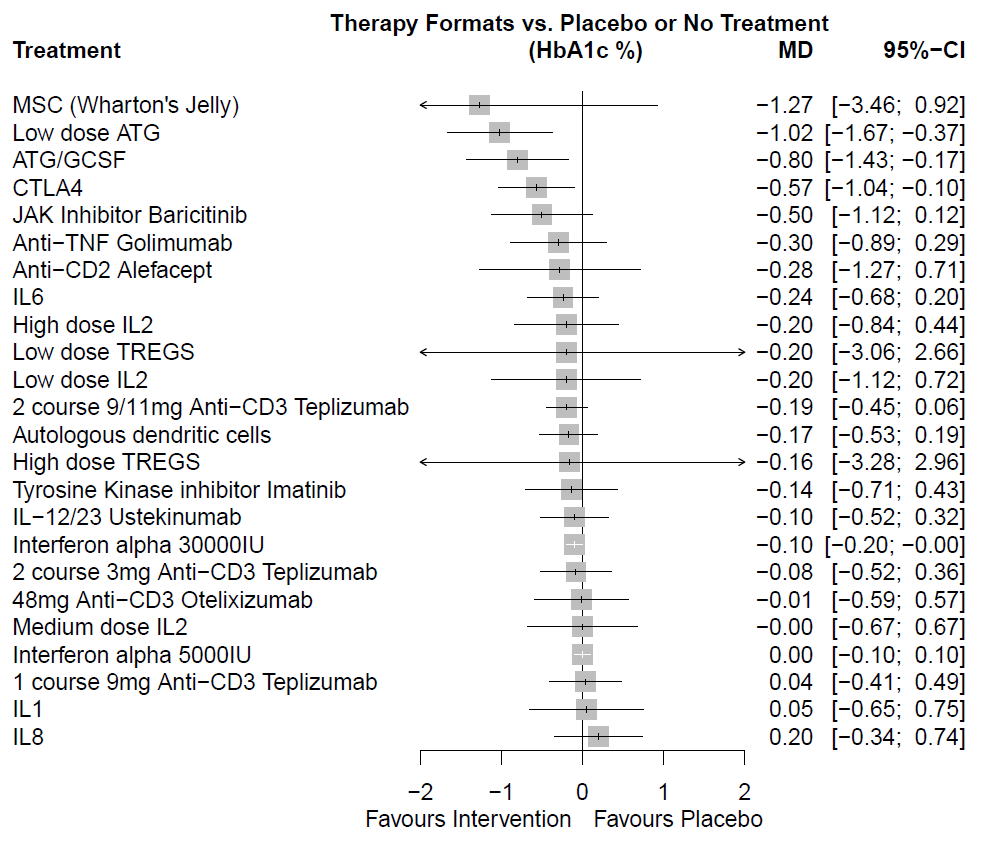


*Outcome metric is mean difference (and 95% confidence intervals). Summary estimates for each intervention versus comparator are listed. Effect estimates to the left of the line of no effect demonstrate a reduction in HbA1c compared to placebo/no comparator.*


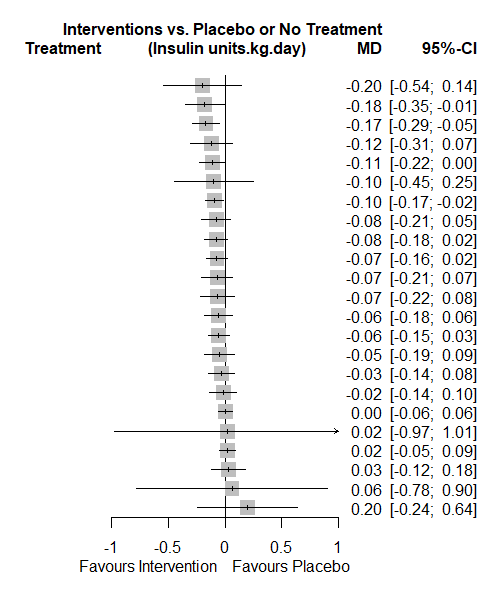
Figure S6. Forest plot displaying results of network meta-analysis of daily insulin doses (units/kg/day) in a subset of therapies which were considered low risk of bias from trials in the main analysis.

Medium dose IL2

CTLA4

Anti-TNF Golimumab

Anti-CD2 Alefacept

JAK Inhibitor Baricitinib

Low dose IL2

2 course 9/11mg Anti-CD3 Teplizumab

Low dose ATG

1 course 9mg Anti-CD3 Teplizumab

2 course 3mg Anti-CD3 Teplizumab

Tyrosine Kinase inhibitor Imatinib

48mg Anti-CD3 Otelixizumab

IL8

Autologous dendritic cells

ATG/GCSF

IL1

IL6

Interferon alpha 30000IU

High dose TREGS

Interferon alpha 5000IU

IL-12/23 Ustekinumab

Low dose TREGS

High dose IL2

*Outcome metric is mean difference (and 95% confidence intervals). Summary estimates for each intervention versus comparator are listed. Effect estimates to the left of the line of no effect demonstrate a reduction in daily insulin dose compared to placebo/no comparator.*

Table 8. Summary of heterogeneity and inconsistency results across analyses

| Analysis | Overall network I^2^ % (95% confidence interval) | Tau^2^ | Tau | Global consistency assessment: Full design-by-treatment interaction model P-value |
| --- | --- | --- | --- | --- |
| Main analysis all C-peptide data | 66.5 (34.5 – 82.8) | 0.101 | 0.318 | 0.004 |
| Low risk of bias analysis | 94 (81 – 98.1) | 0.232 | 0.482 | < 0.001 |
| Sensitivity analyses of C-peptide | | | | |
| Removal of baseline imbalances | 73.2% (42.4% - 87.5%) | 0.099 | 0.316 | < 0.001 |
| Mean change from baseline data | 65.4% (0.0% - 92.1%) | 0.039 | 0.198 | --  (small no. comparisons) |
| Endpoint data | 0% (0.0% - 67.6%) | 0 | 0 | 0.642 |
| Main analysis but with 4-hour AUC data instead of 2-hour | 65.5% (32.5% - 82.4%) | 0.097 | 0.312 | <0.001 |
| 2 and 4-hour AUC C-peptide data only | 77.6 (50.2 – 89.9) | 0.117 | 0.342 | 0.002 |
| Secondary outcomes | | | | |
| HbA1c | 14.1 (0 – 56.9) | 0.021 | 0.145 | 0.327 |
| Low risk of bias analysis | 0 | 0 | 0 | 0.525 |
| Insulin dose | 11.9 (0 – 74.3) | <0.001 | 0.02 | 0.603 |
| Low risk of bias analysis | 29.7 (Not given) | 0.001 | 0.031 | 0.233 |

*Heterogeneity is reported with the I^2^ and Tau^2^ metrics. I^2^ describes the percentage of the variability in effect estimates that is due to heterogeneity rather than sampling error (chance). I^2^ levels of 0-40% indicate levels which may not be important, 30-60% may represent moderate heterogeneity, 50-90% may represent substantial heterogeneity and 75-100% may represent considerable heterogeneity. Tau^2^ reflects an estimate of the between-study variance whilst tau demonstrates the estimated standard deviation of underlying effects across studies.*

*Global inconsistency (assessment of coherence between direct and indirect evidence in the network) is reported with a full design-by-treatment interaction model, with p-values of <0.05 demonstrating statistically significant inconsistency.*

Figure S7. Sensitivity analysis: Forest plot displaying results of network meta-analysis of C-peptide data in a sensitivity analysis removing trials with baseline imbalances in C-peptide


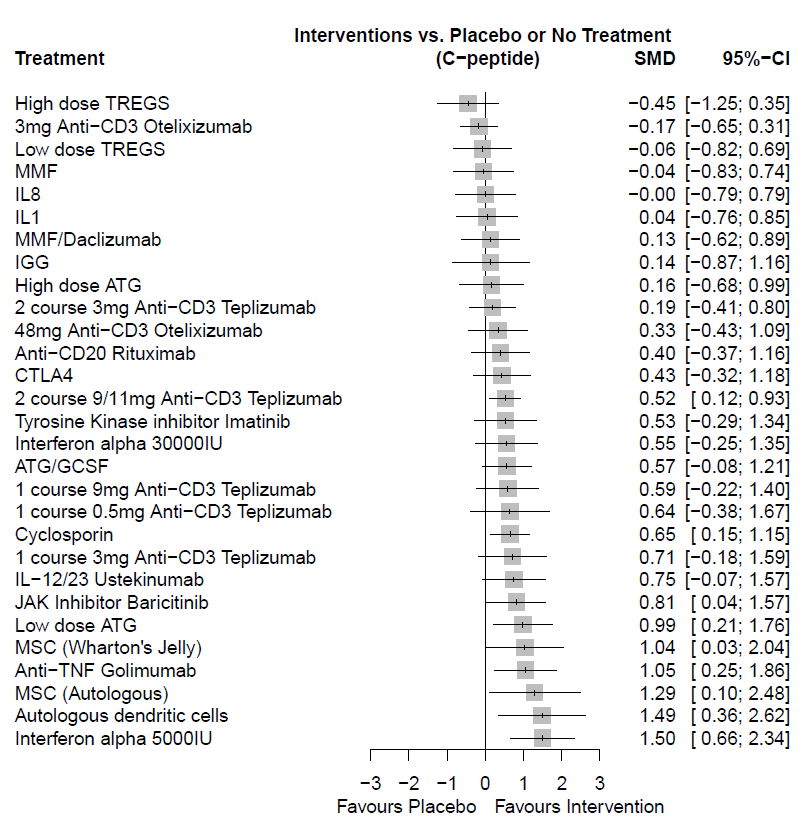


*Outcome metric is standardised mean difference (and 95% confidence intervals). Summary estimates for each intervention versus comparator are listed. Effect estimates to the right of the line of no effect demonstrate a higher level of C-peptide compared to placebo/no comparator at 12 months.*

Table 9. Sensitivity analysis: Median intervention rankings and 95% confidence intervals where trials with baseline imbalances have been removed

| **Intervention** | **Median Ranking** | **95% Confidence interval** |
| --- | --- | --- |
| Autologous dendritic cells | 3 | 1 – 14 |
| Interferon alpha 5000IU | 3 | 1 – 10 |
| MSC (Autologous) | 3 | 1 – 20 |
| Anti-TNF Golimumab | 6 | 2 – 16 |
| Low dose ATG | 6 | 2 – 18 |
| MSC (Wharton's Jelly) | 6 | 1 – 20 |
| JAK Inhibitor Baricitinib | 9 | 2 – 20 |
| IL-12/23 Ustekinumab | 10 | 3 – 22 |
| 1 course 3mg Anti-CD3 Teplizumab | 11 | 3 – 25 |
| 1 course 0.5mg Anti-CD3 Teplizumab | 12 | 2 – 27 |
| Cyclosporin | 12 | 5 – 19 |
| 1 course 9mg Anti-CD3 Teplizumab | 13 | 4 – 26 |
| ATG/GCSF | 13 | 5 – 23 |
| 2 course 9/11mg Anti-CD3 Teplizumab | 14 | 8 – 21 |
| Interferon alpha 30000IU | 14 | 4 – 25 |
| Tyrosine Kinase inhibitor Imatinib | 14 | 4 – 26 |
| CTLA4 | 16 | 5 – 26 |
| Anti-CD20 Rituximab | 17 | 6 – 27 |
| 48mg Anti-CD3 Otelixizumab | 18 | 6 – 28 |
| 2 course 3mg Anti-CD3 Teplizumab | 20 | 10 – 28 |
| High dose ATG | 21 | 8 – 29 |
| IGG | 22 | 7 – 30 |
| MMF/Daclizumab | 22 | 10 – 39 |
| IL1 | 23 | 9 – 30 |
| IL8 | 24 | 12 – 30 |
| Placebo/No treatment | 24 | 21 – 27 |
| Low dose TREGS | 25 | 14 – 30 |
| MMF | 25 | 12 – 30 |
| 3mg Anti-CD3 Otelixizumab | 26 | 19 – 30 |
| High dose TREGS | 29 | 20 – 30 |

Figure S8. Sensitivity analysis: Forest plot displaying results of network meta-analysis of mean change from baseline C-peptide data


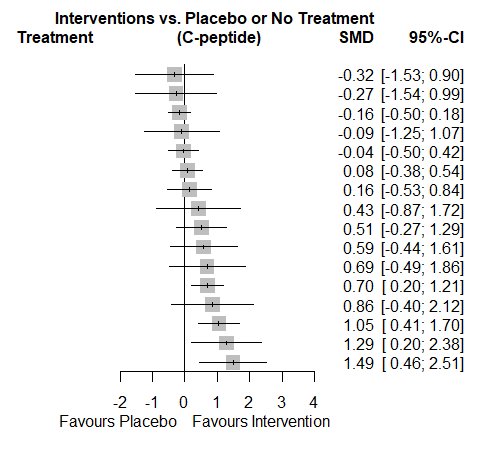


Low dose IL2

18mg Anti-CD3 Otelixizumab

3mg Anti-CD3 Otelixizumab

High dose IL2

2 course 3mg Anti-CD3 Teplizumab

2 course 9/11mg Anti-CD3 Teplizumab

High dose ATG

27mg Anti-CD3 Otelixizumab

Anti-CD2 Alefacept

GCSF

Medium dose IL2

Cyclosporin

9mg Anti-CD3 Otelixizumab

Anti-TNF Golimumab

MSC (Autologous)

Autologous dendritic cells

*Outcome metric is standardised mean difference (and 95% confidence intervals. Summary estimates for each intervention versus comparator are listed. Effect estimates to the right of the line of no effect demonstrate a higher level of C-peptide compared to placebo/no comparator.*

Table 10. Sensitivity analysis: Median intervention rankings and 95% confidence intervals for trials reporting C-peptide mean change from baseline data

| **Intervention** | **Median Rank** | **95% Confidence interval** |
| --- | --- | --- |
| Autologous dendritic cells | 2 | 1 – 7 |
| MSC (Autologous) | 3 | 1 – 8 |
| Anti-TNF Golimumab | 4 | 1 – 8 |
| 9mg Anti-CD3 Otelixizumab | 5 | 1 – 13 |
| Cyclosporin | 6 | 3 – 10 |
| Medium dose IL2 | 6 | 2 – 15 |
| GCSF | 7 | 2 – 16 |
| Anti-CD2 Alefacept | 8 | 3 – 14 |
| 27mg Anti-CD3 Otelixizumab | 8 | 2 – 16 |
| High dose ATG | 11 | 6 – 16 |
| 2 course 9/11mg Anti-CD3 Teplizumab | 11 | 7 – 16 |
| Placebo/No treatment | 12 | 9 – 15 |
| High dose IL2 | 13 | 5 – 17 |
| 2 course 3mg Anti-CD3 Teplizumab | 13 | 8 – 16 |
| 3mg Anti-CD3 Otelixizumab | 14 | 10 – 17 |
| 18mg Anti-CD3 Otelixizumab | 15 | 6 – 17 |
| Low dose IL2 | 16 | 7 – 17 |

Figure S9. Sensitivity analysis: Forest plot displaying results of network meta-analysis of endpoint C-peptide data


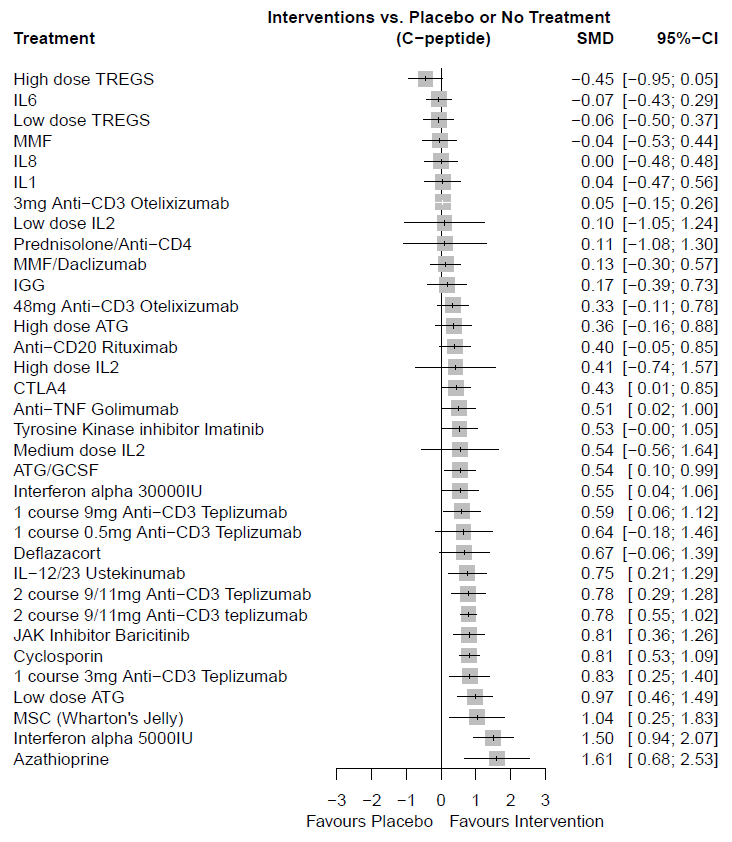


*Outcome metric is standardised mean difference (and 95% confidence intervals). Summary estimates for each intervention versus comparator are listed. Effect estimates to the right of the line of no effect demonstrate a higher level of C-peptide compared to placebo/no comparator.*

Table 11. Sensitivity analysis: Median intervention rankings and 95% confidence intervals for trials reporting C-peptide endpoint data

| **Intervention** | **Median Rank** | **95% Confidence interval** |
| --- | --- | --- |
| Azathioprine | 2 | 1 – 9 |
| Interferon alpha 5000IU | 2 | 1 – 5 |
| MSC (Wharton's Jelly) | 5 | 1 – 20 |
| Low dose ATG | 6 | 2 – 16 |
| 1 course 3mg Anti-CD3 Teplizumab | 9 | 3 – 21 |
| Cyclosporin | 9 | 4 – 16 |
| JAK Inhibitor Baricitinib | 9 | 4 – 19 |
| 2 course 9/11mg Anti-CD3 teplizumab | 10 | 5 – 16 |
| 2 course 9/11mg Anti-CD3 Teplizumab | 10 | 4 – 21 |
| IL-12/23 Ustekinumab | 11 | 3 – 22 |
| 1 course 0.5mg Anti-CD3 Teplizumab | 13 | 3 – 29 |
| Deflazacort | 13 | 3 – 28 |
| 1 course 9mg Anti-CD3 Teplizumab | 15 | 5 – 26 |
| ATG/GCSF | 15 | 7 – 24 |
| Interferon alpha 30000IU | 15 | 6 – 26 |
| Medium dose IL2 | 15 | 2 – 33 |
| Anti-TNF Golimumab | 17 | 6 – 26 |
| CTLA4 | 18 | 9 – 27 |
| Anti-CD20 Rituximab | 19 | 10 – 28 |
| High dose IL2 | 19 | 2 – 34 |
| High dose ATG | 20 | 8 – 31 |
| 48mg Anti-CD3 Otelixizumab | 21 | 11 – 30 |
| IGG | 25 | 14 – 34 |
| MMF/Daclizumab | 25 | 17 – 33 |
| Low dose IL2 | 26 | 6 – 35 |
| 3mg Anti-CD3 Otelixizumab | 27 | 23 – 32 |
| IL1 | 27 | 17 – 34 |
| Prednisolone/Anti-CD4 | 27 | 4 – 35 |
| IL8 | 29 | 20 – 34 |
| Placebo/No treatment | 29 | 25 – 32 |
| IL6 | 30 | 23 – 34 |
| Low dose TREGS | 30 | 21 – 34 |
| MMF | 30 | 20 – 34 |
| High dose TREGS | 34 | 30 – 35 |

Figure S10. Sensitivity analysis: Forest plot displaying results of network meta-analysis of all C-peptide data but with 4-hour AUC data used in place of 2-hour AUC data where a trial reported both measures


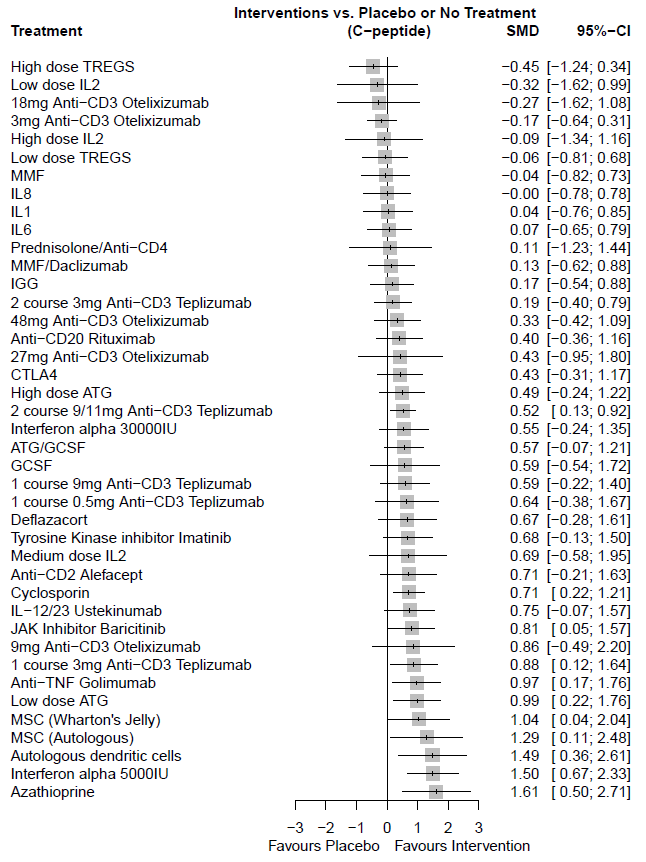


*Outcome metric is standardised mean difference (and 95% confidence intervals). Summary estimates for each intervention versus comparator are listed. Effect estimates to the right of the line of no effect demonstrate a higher level of C-peptide compared to placebo/no comparator.*

Table 12. Sensitivity analysis: Median intervention rankings and 95% confidence intervals for all C-peptide data but with 4-hour AUC data used in place of 2-hour AUC data where a trial reported both measures

| **Intervention** | **Median Rank** | **95% Confidence Interval** |
| --- | --- | --- |
| Azathioprine | 3 | 1 – 16 |
| Interferon alpha 5000IU | 3 | 1 – 13 |
| Autologous dendritic cells | 4 | 1 – 20 |
| MSC (Autologous) | 5 | 1 – 25 |
| MSC (Wharton's Jelly) | 8 | 2 – 27 |
| Anti-TNF Golimumab | 10 | 3 – 25 |
| Low dose ATG | 10 | 3 – 25 |
| 1 course 3mg Anti-CD3 Teplizumab | 12 | 4 – 27 |
| 9mg Anti-CD3 Otelixizumab | 12 | 2 – 37 |
| JAK Inhibitor Baricitinib | 13 | 4 – 28 |
| Cyclosporin | 15 | 7 – 26 |
| IL-12/23 Ustekinumab | 15 | 4 – 31 |
| Medium dose IL2 | 15 | 2 – 38 |
| Anti-CD2 Alefacept | 16 | 4 – 33 |
| Deflazacort | 16 | 4 – 35 |
| Tyrosine Kinase inhibitor Imatinib | 16 | 5 – 33 |
| 1 course 0.5mg Anti-CD3 Teplizumab | 18 | 3 – 37 |
| 1 course 9mg Anti-CD3 Teplizumab | 19 | 6 – 35 |
| ATG/GCSF | 19 | 7 – 33 |
| GCSF | 19 | 4 – 39 |
| Interferon alpha 30000IU | 19 | 6 – 36 |
| 2 course 9/11mg Anti-CD3 Teplizumab | 20 | 12 – 28 |
| High dose ATG | 20 | 7 – 36 |
| CTLA4 | 22 | 8 – 37 |
| 27mg Anti-CD3 Otelixizumab | 23 | 3 – 41 |
| Anti-CD20 Rituximab | 24 | 8 – 36 |
| 48mg Anti-CD3 Otelixizumab | 25 | 9 – 38 |
| 2 course 3mg Anti-CD3 Teplizumab | 28 | 16 – 39 |
| IGG | 29 | 14 – 40 |
| MMF/Daclizumab | 29 | 14 – 40 |
| IL6 | 31 | 16 – 40 |
| Prednisolone/Anti-CD4 | 31 | 6 – 42 |
| IL1 | 32 | 16 – 41 |
| IL8 | 33 | 17 – 41 |
| MMF | 33 | 18 – 41 |
| Placebo/No treatment | 33 | 29 – 36 |
| Low dose TREGS | 34 | 19 – 41 |
| High dose IL2 | 35 | 10 – 42 |
| 3mg Anti-CD3 Otelixizumab | 36 | 26 – 41 |
| 18mg Anti-CD3 Otelixizumab | 38 | 13 – 42 |
| Low dose IL2 | 38 | 13 – 42 |
| High dose TREGS | 39 | 27 – 42 |

Figure S11. Sensitivity analysis: Forest plot displaying results of network meta-analysis of all 2 and 4-hour AUC C-peptide data only (2-hour data where both measures of C-peptide were reported)


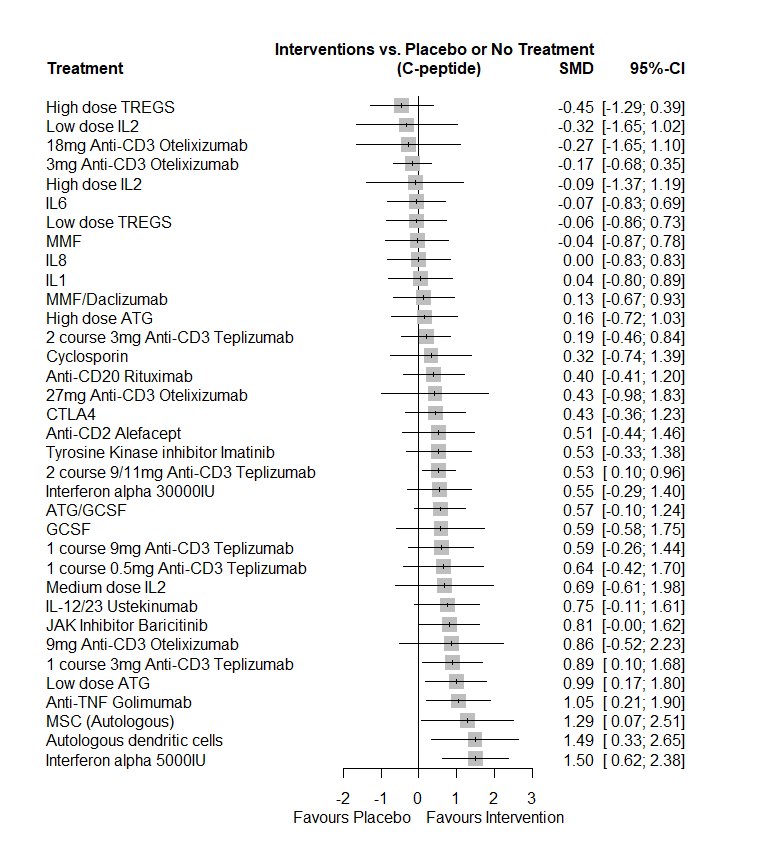


*Outcome metric is standardised mean difference (and 95% confidence intervals). Summary estimates for each intervention versus comparator are listed. Effect estimates to the right of the line of no effect demonstrate a higher level of C-peptide compared to placebo/no comparator.*

# Post-hoc analysis

In a post-hoc sensitivity analysis undertaken on duration of type 1 diabetes at baseline, all studies in the NMA had duration inclusion criteria or mean/median duration of <7 months (all studies had less than 6 months duration except for one). Two studies had a longer mean duration of 12 months (Haller 2014b (ATG/GCSF) and Gaglia 2024 (Autologous dendritic cells)). Removal of these studies in the post-hoc analysis made no difference to the results, other than removing autologous dendritic cells from the analysis and the slight attenuation of the effect of ATG/GCSF due to the removal of one of these trials. See below for ranking results.

Table 13. Table of probabilities of each intervention being ranked first as frequentist p-scores, with the new post-hoc analysis results on the left-hand side and the original analysis results on the right-hand side.

| **New analysis with studies removed** | **P-score** | **Original analysis** | **P-score** |
| --- | --- | --- | --- |
| Interferon alpha 5000IU | 0.9155 | Interferon alpha 5000IU | 0.9080 |
| Azathioprine | 0.9139 | Azathioprine | 0.9072 |
| MSC (Autologous) | 0.8333 | Autologous dendritic cells | 0.8804 |
| Anti-TNF Golimumab | 0.7911 | MSC (Autologous) | 0.8247 |
| MSC (Wharton's Jelly) | 0.7673 | Anti-TNF Golimumab | 0.7807 |
| Low dose ATG | 0.7477 | Low dose ATG | 0.7577 |
| 1 course 3mg Anti-CD3 Teplizumab | 0.7269 | MSC (Wharton's Jelly) | 0.7570 |
| JAK Inhibitor Baricitinib | 0.6908 | 1 course 3mg Anti-CD3 Teplizumab | 0.7133 |
| 9mg Anti-CD3 Otelixizumab | 0.6764 | JAK Inhibitor Baricitinib | 0.6791 |
| IL-12/23 Ustekinumab | 0.6603 | 9mg Anti-CD3 Otelixizumab | 0.6660 |
| Cyclosporin | 0.6245 | IL-12/23 Ustekinumab | 0.6485 |
| Deflazacort | 0.6138 | Cyclosporin | 0.6115 |
| Medium dose IL2 | 0.6113 | Deflazacort | 0.6020 |
| 1 course 0.5mg Anti-CD3 Teplizumab | 0.5997 | Medium dose IL2 | 0.6004 |
| 1 course 9mg Anti-CD3 Teplizumab | 0.5821 | 1 course 0.5mg Anti-CD3 Teplizumab | 0.5881 |
| GCSF | 0.5686 | 1 course 9mg Anti-CD3 Teplizumab | 0.5698 |
| Interferon alpha 30000IU | 0.5622 | ATG/GCSF | 0.5615 |
| 2 course 9/11mg Anti-CD3 Teplizumab | 0.5598 | GCSF | 0.5572 |
| Tyrosine Kinase inhibitor Imatinib | 0.5495 | Interferon alpha 30000IU | 0.5499 |
| Anti-CD2 Alefacept | 0.5386 | 2 course 9/11mg Anti-CD3 Teplizumab | 0.5449 |
| High dose ATG | 0.5385 | Tyrosine Kinase inhibitor Imatinib | 0.5371 |
| ATG/GCSF | 0.5219 | Anti-CD2 Alefacept | 0.5265 |
| CTLA4 | 0.5006 | High dose ATG | 0.5208 |
| 27mg Anti-CD3 Otelixizumab | 0.4955 | CTLA4 | 0.4880 |
| Anti-CD20 Rituximab | 0.4814 | 27mg Anti-CD3 Otelixizumab | 0.4849 |
| 48mg Anti-CD3 Otelixizumab | 0.4471 | Anti-CD20 Rituximab | 0.4688 |
| 2 course 3mg Anti-CD3 Teplizumab | 0.3658 | 48mg Anti-CD3 Otelixizumab | 0.4346 |
| Prednisolone/Anti-CD4 | 0.3606 | 2 course 3mg Anti-CD3 Teplizumab | 0.3534 |
| IGG | 0.3572 | Prednisolone/Anti-CD4 | 0.3507 |
| MMF/Daclizumab | 0.3435 | IGG | 0.3455 |
| IL1 | 0.3014 | MMF/Daclizumab | 0.3317 |
| IL8 | 0.2789 | IL1 | 0.2901 |
| High dose IL2 | 0.2743 | IL8 | 0.2679 |
| MMF | 0.2573 | High dose IL2 | 0.2651 |
| Low dose TREGS | 0.2493 | MMF | 0.2467 |
| Placebo/No treatment | 0.2434 | Low dose TREGS | 0.2387 |
| IL6 | 0.2434 | Placebo/No treatment | 0.2336 |
| 18mg Anti-CD3 Otelixizumab | 0.2152 | IL6 | 0.2327 |
| Low dose IL2 | 0.1998 | 18mg Anti-CD3 Otelixizumab | 0.2073 |
| 3mg Anti-CD3 Otelixizumab | 0.1805 | Low dose IL2 | 0.1921 |
| High dose TREGS | 0.1112 | 3mg Anti-CD3 Otelixizumab | 0.1717 |
|  |  | High dose TREGS | 0.1043 |

# Adverse events

Table 14. Summary of adverse events of all trials included in the review

| **Study ID** | **Sub-Group** | **Follow up time AEs reported** | **No. patients** | **Intervention** *No. pts with events/no. events* | **Comparator** *No. pts with events/no. events* | **Hypoglycaemia?** | **Ketoacidosis?** | **Other key data** | **No. pts who discontinued study (intervention)** | **No. pts who discontinued study (comparator)** | **Side effects directly relating to intervention?** |
| --- | --- | --- | --- | --- | --- | --- | --- | --- | --- | --- | --- |
| Rigby 2013 | Alefacept | 24m (no. discontinued is only reported at 12m) | Intervention: 33 Comparator: 16 | Serious AEs 1 participant had 1 event AEs 1076 events in 33 participants (all participants experienced an event) | Serious AEs 0 AEs 707 events in 16 participants (all participants experienced an event) | 609 major hypoglycaemic events in 30 participants in intervention group 525 major hypoglycaemic events in 15 participants in placebo group | NR | There was no significant between-group difference in glycaemic control or adverse events | 3 LTFU | 4 LTFU | Serious AEs related to study drug 0 AEs related to study drug: intervention 365 events in 29 participants; placebo 198 events in 15 participants |
| Herold 2002 | Teplizumab | 12m | Intervention: 12  Comparator: 12 | AEs: total number of participants with events NR  9 had fever  9 had anaemia  1 nausea  1 vomiting  1 arthralgia  1 headache  7 pruritic urticarial rash | NR | No severe hypoglycaemia events in either arm | NR | NR | NR | NR | NR |
| Herold 2005 | Anti-CD3 | Yes; all adverse events; Over 24 months | Intervention: 21 Comparator: 21 | NR | NR | NR | NR | Fever (36.4%), headache (72.7%), myalgia (22.7%), or arthralgia (13.6%), generally occurring with the first full dose of the drug on the 3rd or 5th day of drug administration.  Less frequent adverse events included nausea, diarrhoea, or vomiting; rigors; or fatigue. These were generally mild and controlled with nonsteroidal anti-inflammatory drugs, acetaminophen, and/or antihistamine and resolved after the initial full doses of the drug.  90.9% Rash (90.9%), generally after the 5th dose of drug. One patient developed grade 3 thrombocytopenia. | 0 | n=1 LTTFU at 6 months n=1 became pregnant after month 18 of follow up | Cytokine release syndrome-like symptoms/signs are consistent with the finding of cytokine release after drug administration that has been reported previously. Rashes: generally urticarial appearance, involved the hands, but distribution and appearance were variable. Intervention caused a decrease in the number of circulating lymphocytes. The nadir in lymphocyte count generally occurred after the first full dose of drug, but the number of circulating cells increased thereafter despite continued administration of the drug.  Patients were followed for 2 years and were seen by their physicians and/or the study investigators at ~3- to 6-month intervals.  No long-term side effects could be related to drug treatment. None of the patients developed an unusual infectious illness, and no changes in the number or types of circulating lymphocytes were detected. |
| Herold 2009 | Anti-CD3 | 24m | Intervention: 6 Comparator: 6 | 202 events in 6 patients (2 patients reported serious AEs) | 50 events in 4 patients (1 patient reported serious AEs) | NR | NR | NR | 0 | 0 | Of the 202, 67% were judged to be related to the use of the study drug |
| Sherry 2011 | Anti-CD3 | 24m | 14-day full dose 209 14-day low dose 102 6-day full dose 106 Comparator: 99 | 417 patients in total 14-day full-dose group 207 events in 209 patients (19 serious AEs);  14-day low-dose group 101 events in 102 patients (11 serious AEs);  6-day full-dose group 106 events in 106 patients (12 serious AEs) | 98 events in 99 patients (9 serious AEs) | 1 event reported in each group of patients (n=4 in total) | 14-day full dose group 5 events 14-day low dose group 3 events 6-day full dose group 1 event 0 in placebo group | NR | Across all groups:  18 LTTFU 23 withdrew consent 4 withdrew due to adverse events 2 other | 5 LTTFU 2 withdrew consent | Grade 3 adverse events were increased in teplizumab groups, but this difference versus placebo was primarily due to lymphopenia, an expected consequence of the mechanism of action. In particular, no differences were apparent between groups in the incidence of infections overall, or by specific types, with the possible exception of herpes zoster.  No rashes or cytokine release events occurred during the second year because the drug was not administered during this period. |
| Herold 2013 a | Anti-CD3 | 12m | Intervention: 34 Comparator: 27 | 711 events in 33 patients (1 serious event) | 644 events in 27 patients (4 serious event) | NR | NR | NR | 3 withdrew before treatment was given | 2 withdrew before treatment  1 LTTFU | There were 30 treatment-related adverse events in the teplizumab group and 25 in the placebo group All adverse events resolved and were expected based on past experiences with teplizumab in type 1 diabetes |
| Herold 2013 b | Anti-CD3 | 24m | Intervention: 56 Comparator: 27 | 1227 events in 52 patients (11 serious events) | 228 events in 23 of 25 patients (2 serious events) | 21 of 52 patients in the intervention group experienced 26 events 6 of 25 patients in the placebo group experienced 7 events | 1 event in the intervention group only | NR | 4 withdrew after randomisation but before receiving the treatment | 2 withdrew before baseline data taken | Drug-related events were transient and resolved |
| Keymeulen 2005 | Anti-CD3 | 48 months | Intervention: 20 Comparator: 20 | at 12 months - 40 patients: 38 with fever, 40 headache, 39 GI symptoms, 40 arthralgia, 35 myalgia, 29 rash at day 11-15, 30 sore throat, 13 fever, 10 cervical adenopathy | at 12 months - 40 patients: 1 with fever, 14 headache, 7 GI symptoms, 2 arthralgia, 8 myalgia, 2 rash at day 11-15, 3 sore throat, 1 fever, 3 cervical adenopathy | Severe hypoglycaemia; Intervention group 1 patient; Comparator group 3 patients | NR | Over the 48 month follow-up period, no biological or clinical signs of EBV reactivation or EBV-related disease were observed; there was no higher incidence of infections, and no lymphoma or other types of cancer. No differences in CD3+ lymphocyte counts were found between months 6 and48 | During 0-18 months 1 discontinued treatment  1 pregnancy | During 0-18 months 2 withdrew from further testing 2 moved abroad | NR |
| Ambery 2014 | Anti-CD3 | 12 months | Intervention: 118 Comparator: 61 | 112 of 118 patients reported any adverse events | 54 of 61 patients reported any adverse events | Hypoglycaemia; Intervention group 13 patients; Comparator group 6 patients | NR | NR | 3 Subject/legal representative request  1 Investigator recommended  4 Admin Reasons  6 LTTFU 0 Adverse Event  1 Other | 7 Subject/legal representative request 1 Investigator recommended  0 Admin Reasons  2 LTTFU 1 Adverse Event  0 Other | The most commonly reported adverse events included headache, nausea and fatigue, consistent with cytokine release. No Epstein–Barr virus reactivation was seen |
| Aronson 2014 | Anti-CD3 | 12 months | Intervention: 181 Comparator: 91 | 181 patients: 155 with headache, 60 with nausea, 35 with vomiting, 34 with pyrexia, 24 with chills, 23 with hypoglycaemia, 21 with myalgia, 20 with arthralgia | 91 patients: 45 with headache, 20 with nausea, 13 with vomiting, 10 with pyrexia, 6 with chills, 8 with hypoglycaemia, 14 with myalgia, 2 with arthralgia | Hypoglycaemia; intervention group 23 patients (severe episode 4 patients); Comparator 8 patients (severe episode 2 patients) | NR | NR | 2 Lost to follow-up 6 Requested withdrawal 1 Investigator recommendation  0 Other | 3 Lost to follow-up  4 Requested withdrawal  1 Investigator recommendation  1 Other | Adverse events were more common in the otelixizumab group, and included, for example, headache, fever, rash, nausea, which were consistent with known side effects of anti-CD3 antibodies |
| Keymeulen 2021 | Anti-CD3 | 24m | 9mg: 9 18mg: 8 27mg: 7 Comparator: 5 | During dosing: 2 patients had events leading to permanent discontinuation of study treatment 1 patient had serious AE related to study treatment Post dose to week 6: 21/24 patients had AEs related to study treatment Week 6-month 24: 18/24 patients had AEs related to study treatment | During dosing: 4 patients had AEs related to study treatment Post dose to week 6: 3 patients had AEs related to study treatment Week 6-month 24: 5 patients had AEs related to study treatment | NR | NR | One individual in the 9 mg group on active treatment stopped participation before the end of dosing because of CRS-related AEs (which did not meet the protocol stopping criteria), and one participant in the 27 mg group on active treatment was diagnosed with cytomegalovirus primoinfection on day 5 of the dosing period, reported as a drug related SAE. This participant was not given the last dose of otelixizumab (day 6) and was immediately treated as per standard of care (ganciclovir). Beyond Week 6, no patterns were observed in AEs related to study treatment, and there were no differences between active treatment and placebo. | 1 LTTFU (18mg group) | 1 withdrew before treatment  1 withdrew during f/up | The frequency and severity of AEs were dose dependent, with a higher frequency in participants receiving otelixizumab (vs placebo) around the time of dosing. During the dosing period, drug-related grade 2 and 3 AEs occurred with all otelixizumab doses and in similar percentages of participants AEs related to study treatment at dosing: All patients in the otelixizumab groups experienced an AE  Placebo: 4 patients 1 serious AE in the 27mg group |
| Saudek 2004 | ATG | 12m | Intervention: 11 Comparator: 6 | All 11 participants had a rise in body temperature following ATG administration 7 participants had chills 2 participants had phlebitis 6 participants elevated body temperature and arthralgia attributable to serum sickness 1 participant had transient lymphadenopathy | 1 participant had transient lymphadenopathy  1 participant had low grade fever | NR | NR | N/A | 4  (1 refused further ATG doses after the first) | 2 (reasons NR) | No adverse event attributable to the study treatment occurred in any patient later than one month after study entry. |
| Gitelman 2013 | ATG | 24m | Intervention: 38 Comparator: 20 | *Report AEs that were reported in 15% or more participants at 24m* 38 participants had 1148 events 5 participants had at least 1 serious AE | *Report AEs that were reported in 15% or more participants at 24m* 20 participants had 415 events 2 participants had at least 1 serious AE | Intervention 495 events in 38 participants (1 serious) Placebo 201 events in 16 participants | 1 participant in the placebo group had serious ketoacidosis | AEs were also evaluated by age group of the participants, grouping them into younger (12–21 years) and older (22–35 years) cohorts, and we did not find an appreciable difference in the nature or severity of the early events (CRS and serum sickness) or later findings between the younger and older ATG-treated participants. | 3 not assessed at 12m onwards 1 pregnant at the time 2 improper sample handling | 4 LTTFU | Almost all participants in the treatment group experienced cytokine release syndrome during the ATG infusion, and all experienced serum sickness 1–2 weeks later, with complete resolution of all symptoms within the first 3–4 weeks after therapy. A comparable number of infections per participant was noted in the ATG and placebo groups. |
| Haller 2018 | ATG/GCSF | 24m | ATG/GCSF: 29 ATG: 29 Comparator: 31 | 12m ATG only: 29 participants had 152 events  43 events grade 3/4 ATG/GCSF: 28 participants had 161 events  52 events grade 3/4 24m ATG only: 29 participants had 14 events 4 events grade 3/4 ATG/GCSF: 28 participants had 15 events 3 events grade 3/4 | 12m 31 participants had 67 events 12 events grade 3/4 24m  31 participants had 27 events 9 events grade 3/4 | ATG/GCSF group: 1 participant had 1 event Placebo group: 2 participants had 2 events | NR | N/A | ATG group  1 participant withdrew ATG/GCSF group  1 participant withdrew after randomisation | 5 withdrew consent | First 12m: No subjects required extended hospitalisation or readmission due to cytokine release or serum sickness. No subjects who received ATG/GCSF or ATG alone developed a serious infection. There were no cases of grade 4 serum sickness or cytokine release. There were no grade 5 adverse events 12-24m: There were no increases in AEs in ATG- or ATG/GCSF-treated subjects |
| Haller 2014b | ATG/GCSF | 24m | Intervention: 17 Comparator: 8 | 12m: 520 events across both groups 24m: 21 events in 17 participants | 12m: 520 events across both groups 24m: 10 events in 8 participants | 12m: No episodes of severe hypoglycaemia were observed, but numerous episodes of hypoglycaemia were reported 24m: 11 episodes of hypoglycaemia across both groups. 1 grade 3 in intervention group, 1 in the placebo | NR | Of the 17 subjects who received ATG, cytokine release syndrome (CRS) occurred during drug infusion in 14, whereas serum sickness developed in 13. There were no reports of grade 4 CRS or serum sickness. | 1 LTTFU | 0 | 24m: One patient in the ATG+G-CSF treatment arm presented transiently with reduced CD4+ T-cell counts possibly related to treatment There were no differences in reported frequency of infections in either group at any follow up |
| Harrison 1985 | Azathioprine | 12m | Intervention: 13 Comparator: 11 | One patient developed lymphopenia and another thrombocytopenia within 2 weeks of starting treatment, necessitating temporary withdrawal of azathioprine for 3 wks. and reintroduction at half dose | None reported | NR | NR | N/A | 5 patients discontinued treatment (though continued in the trial) | 0 |  |
| Cook 1989 | Azathioprine | 12m | Intervention: 25 Comparator: 25 | 5 skin lesions 5 neutropenia 1 minor transient rise in serum aspartate transaminase levels | 6 neutropenia | NR | NR | Serum immunoglobulin levels, lymphocyte responses to mitogens, and neutrophil function tests were not different between the two groups at 3 and 12 m | 1 Did not return for follow up and was withdrawn | 0 | There was no statistical difference in the number of infections between the two groups. All infections were mild. However, skin lesions, which included two cases of acne, two of multiple warts, and one of herpes simplex, all occurred in azathioprine-treated patients. |
| Pescovitz 2009 | B-cell rituximab | 24m | Intervention: 57 Comparator: 30 | 57 participants had 392 events 11 participants had 13 serious AEs | 30 participants had 148 events 9 participants had 17 serious AEs | 5 events in 5 participants in intervention group 4 events in 4 participant in placebo group | NR | In the first 12 months, 1 participant in the intervention group had a grade 3 infusion reaction consisting of shortness of breath and rash | 8 1 Did not receive intervention because parent withdrew consent 4 Had infusions suspended  1 Withdrew consent 1 Became pregnant 1 Did not complete the evaluation because of difficulty placing an intravenous catheter | 1 Had infusions suspended owing to FDA safety alert | Infections were slightly more common in the rituximab group, but this difference was not significant. IgM levels fell from baseline in the rituximab group, an effect that persisted at 24 months (P , 0.0001). The IgG concentrations did not differ significantly between the two groups and remained similar to that at baseline. No subject required treatment for hypogammaglobulinemia. There were no adverse effects on routine laboratory parameters. |
| Zielinski 2022 | B-cell rituximab | 24m | Intervention: 12 Comparator: 13 | Intervention: 12 patients reported 76 events Infections/infestations: 7 patients with 11 events Metabolism/nutritional disorders: 3 patients with 8 events Blood/lymphatic disorders: 4 patients with 4 events GI disorders: 4 patients with 14 events Nervous system disorders: 2 patients with 5 events Respiratory, thoracic and mediastinal disorders: 3 patients with 5 events | Comparator: 9 patients reported 28 events Infections/infestations: 6 patients with 9 events Metabolism/nutritional disorders: 2 patients with 3 events Blood/lymphatic disorders: 0 events GI disorders: 1 patient with 5 events Nervous system disorders: 1 patient with 1 event Respiratory, thoracic and mediastinal disorders: 0 events | Intervention: 1 patient had an episode Comparator: 1 patient had an episode | Intervention: 0 events Comparator: 1 patient had an event | No deaths or AEs leading to withdrawal of the study drug occurred | 0 | 0 | AE related to the study treatment: Intervention: 12 patients with 59 events Comparator: 8 patients with 10 events |
| Hehmke 1994 | CD4/Pred | 12m | Pred/Anti-CD4 group: 5 Comparator: 6 | 0 | 0 | NR | NR | NR | 0 | 0 | The therapy was well tolerated and no side effects were observed |
| Orban 2011 | CTLA4 | 36m | Intervention: 77 Comparator: 35 | 266 events occurred in all 77 participants. 1 accidental death occurred unrelated to the study | 111 events occurred in all 35 participants. | There were 7 episodes of hypoglycaemia reported as an adverse event, two of which were severe hypoglycaemia (one in each group). | NR | NR | 14 4 declined follow up at 36m 3 LTTFU  1 extended travel  1 admin mistake  2 conflicting responsibilities 1 withdrew consent 1 refused 1 died (unrelated to study) 1 dropped out due to pregnancy but returned afterwards | 8 5 were not assessed at 24 months 2 lost to follow-up 1 withdrew consent 1 refused 1 travel issues 3 declined follow up at 36m | Infusion-related adverse events occurred with low frequency (47 of 2514 infusions [2%] involving 27 patients) and were not clinically significant. Of these, 36 reactions occurred in 17 (22%) of 77 patients on abatacept and 11 reactions in six (17%) of 35 patients on placebo (p=0·62 for proportion of participants by Fisher’s exact test). |
| Vague 1989 | Cyclosporin | 36 months | Intervention: 15 Comparator: 13 | Not reported, though paper states 0 serious adverse events | Not reported, though paper states 0 serious adverse events | NR | NR | NR | 0 | 0 | NR |
| Chase 1990 | Cyclosporin | 36 months | Intervention: 22 Comparator: 21 | NR | NR | NR | NR | Hypertrichosis (16 subjects), fatigue (6 subjects), gingivitis (4 subjects), and paraesthesia (4 subjects) were noted but were never a reason to decrease the cyclosporine A dosage. Elevation of serum creatinine levels as a marker of nephrotoxicity was not detected. Microalbumin levels were <18 ıtg/minute in all subjects at 3 years. Anaemia was not detected in any of the subjects at any time | 2 moved and could not be studied further after 4 months 2 elected not to participate at 36 months | 1 moved and could not be studied further after 4 months 1 elected not to participate at 36 months | NR |
| Martin 1991 | Cyclosporin | 12 month data only | Intervention: 93 Comparator: 94 | Of 93 patients: 87 had upper respiratory tract infection, 14 with GI infection, 27 with skin infection, 58 with gum hyperplasia, 71 with hypertrichosis, 51 with headache, 45 with nausea/vomiting, 17 with diarrhoea | Of 94 patients: 77 had upper respiratory tract infection, 19 with GI infection, 22 with skin infection, 24 with gum hyperplasia, 16 with hypertrichosis, 39 with headache, 27 with nausea/vomiting, 4 with diarrhoea | NR | NR | NR | 3 Nausea, vomiting  1 Hair growth*  1 Hypertension  1 High bilirubin  2 Epstein-Barr virus  1 Noncompliance,  3 test drug noncompliance,  1 birth control  3 Voluntary withdrawal | 1 Headaches  1 Hypertension  2 Pregnancy  4 Noncompliance, test drug  2 Voluntary withdrawal | Follow-up showed normalization of all abnormal parameters within 6 months of discontinuation.  However, previous kidney biopsies had shown structural damage (moderate arteriolopathy, tubular atrophy or interstitial fibrosis) in specimens from some CsA patients |
| Skyler 1992 | Cyclosporin | 12m | Intervention: 12 Comparator: 13 | 5 pts Elevation in serum creatinine (1 cyclosporine subject showed a persistent creatinine of 1.1 mg/dL after completion of the study) 6 pts Elevation in blood urea nitrogen  1 patient global increase in mesangial matrix with linear staining of IgG and complement, consistent with early diabetic glomerulosclerosis, and without either interstitial fibrosis or tubular atrophy on renal biopsy 2 pts mild anaemia 5 pts hirsutism 4 pts gingival hyperplasia  2 pts abdominal discomfort  4 pts paraesthesia | 4 pts Elevation in serum creatinine (1 placebo subject showed a persistent creatinine of 1.3 mg/dL after completion of the study) 2 pts Elevation in blood urea nitrogen  1 pts patient minimal focal and segmental increase in the mesangial matrix on both light and electron microscopy on renal biopsy 1 pt gingival hyperplasia  1 pt abdominal discomfort 1 pt paraesthesia | NR | NR | Of greatest concern has been potential nephrotoxicity. Although inspection of the data concerning serum creatinine and creatinine clearance suggests a difference between groups, closer inspection reveals that baseline differences may account for the separation. This was confirmed by statistical analyses that showed no difference between baseline and 12 month data in either placebo or cyclosporine groups | 0 | 0 | NR |
| Pozzilli 1994 | Cyclosporin | 24m | Cyclosporin and nicotinamide: 30 Nicotinamide: 30 Comparator: 30 | 12m only: 4 pts in CyA/NCT group hair loss 1 pt Hyperbilirubinemia in CyA/NCT group 2 pts raised serum transaminase in CyA/NCT group No side-effects were noted in patients receiving NCT alone. | NR | NR | NR | NR | 8 discontinued (reasons NR) | 2 discontinued (reasons NR) | NR |
| Pozzilli 1994 | Deflazacort | 12m | Deflazacort/Nicotinamide: 18 Comparator: 18 | NR | In one patient of comparator, flush was detected at the first administration of NCT. | NR | NR | NR | 3 patients were unable to follow physician's recommendations and therefore were excluded | 0 | NR |
| Mastrandrea 2009 | Etanercept | 9m | Intervention: 10 Comparator: 8 | 3 very mild episodes of self-resolving paresthesia in 1 subject  reported twice as frequently in the etanercept group compared with the placebo group 9 episodes of abdominal pain | 0 episodes self-resolving paresthesia | NR | NR | The frequency of events was similar in the two groups with the exception of those events listed in the intervention/comparator columns | 0 | 1 discontinued at week 16 due to poor adherence with insulin therapy/meal plan | A positive ANA titer was detected at baseline in ﬁve subjects assigned to etanercept and two assigned to placebo. The ANA titer results at baseline for one subject in the etanercept group were missing. This subject had a positive ANA titer in the samples drawn thereafter. None of the subjects in the etanercept group with negative results at baseline converted to positive ANA status, and one subject in the placebo group converted to positive status after discontinuation of study drug. |
| Haller 2014a | GCSF | 12m | Intervention: 14 Comparator: 7 | 24 events in 14 participants | 5 events in 7 participants | NR | NR | N/A | 2 (reasons not reported) | 0 | NR |
| Hessner 2013 | IL1 | 9m | Intervention: 22 Comparator: 25 | NR | NR | NR | NR | NR | NR | NR | NR |
| Moran 2013a | IL1 | 12m | Intervention: 47 Comparator: 22 | 81 events in 47 participants 1 serious AE of suicidal ideation unrelated to study drug | 40 events in 22 participants 1 serious AE of appendicitis unrelated to study drug | NR | NR | N/A | 2 withdrew consent | 1 withdrew consent | The number and severity of adverse events did not differ between groups. Despite its potential anti-inflammatory effects, canakinumab did not result in more frequent or more severe infections. |
| Moran 2013b | IL1 | 12m | Intervention: 35 Comparator: 34 | 90 events in 35 participants No serious AEs | 51 events in 34 participants No serious AEs | NR | NR | N/A | 10 3 adverse reactions 3 requested withdrawal 2 non-compliance  2 LTTFU | 8 3 had adverse reactions  3 requested withdrawal  1 LTTFU  1 other reason | The anakinra group reported significantly higher grades of adverse events than the placebo group, primarily as a result of higher frequency grade 2 events in the anakinra group. Analysis of AEs by category revealed that dermatological and skin events was the only category significantly different between treatment groups. The difference was attributable to 17 and four participants with injection site reactions from the anakinra and placebo groups, respectively |
| Rosenzwajg 2020 | IL2 | 14m | 0.152MIU/m2 dose: 5 0.25MIU/m2 dose: 6 0.5MIU/m2 dose: 6 Comparator: 7 | 0.152MIU/m2 dose: 76 events 0.25MIU/m2 dose: 102 events 0.5MIU/m2 dose: 124 events | 46 events | NR | NR | No serious adverse events occurred | 1 withdrew due to adverse event at 270 days (in 0.5 dose group) | 0 | During the treatment period, there was a dose–effect relationship for all non-serious AEs taken together. Local reactions at the injection site accounted for most of the common non-serious AEs, from 3.4% of administrations for placebo-treated patients to 37.9% for intervention patients, with a dose–effect relationship corresponding to 26.2%, 36.9% and 47.7% at the 0.125, 0.25 and 0.5 MIU/m2 day−1 doses, respectively. The other non-serious AEs (headache, gastrointestinal symptoms, transient asthenia and fever) had the same frequency in the different therapy groups (23.3%)and placebo (19.2%). |
| Gottlieb 2010 | IL2/MMF | 24m | MMF/DZB: 41 MMF/Plac: 31 Placebo: 41 | MMF/DZB group:  Infection 59 events in 26 participants GI toxicity 13 events in 7 participants Haematologic events 9 events in 7 participants Serious AEs 19 events in 14 participants  MMF/Plac group:  Infection 40 events in 17 participants GI toxicity 12events in 9 participants Haematologic events 14 events in 9 participants  Serious AEs 9 events in 5 participants | Infection 47 events in 24 participants GI toxicity 12 events in 9 participants Haematologic events 9 events in 6 participants Serious AEs 3 events in 3 participants | Major hypoglycaemic events were reported for 27 subjects, with an average of two each, with no difference among groups. | NR | More grade 2 or higher AEs occurred in MMF plus DZB subjects (167 or 4.1 events/subject) compared with MMF alone (117, 3.8 events/subject) or control subjects (133, 3.2 events/subject) | MMF/DZB: 9  MMF/Plac: 7 Reasons not given | 7 (reasons not given) | No difference in the occurrence of infectious or gastrointestinal events among groups. Eight individuals had asymptomatic reactivation of previous EBV infection using a sensitive PCR assay (five in MMF plus DZB, one in MMF, and two in control). Neutropenia and leukopenia, both side effects of MMF and DZB, occurred approximately equally among the three groups. A slight excess of elevated liver enzymes occurred in the MMF plus DZB group. |
| Von Herrath 2021 | IL21 | 18m | Anti IL-21 + Liraglutide 77 Anti IL-21 alone 77 Liraglutide alone 77 Comparator: 77 | Anti IL-21 + Liraglutide: 75.9 patients with events Anti IL-21 alone: 75 patients with events Liraglutide alone: 75.2 patients with events | 74.5 patients with events | Anti IL-21 + Liraglutide: 4419 events per 100 pt years exposure  Anti IL-21: 4560 events per 100 pt years exposure Liraglutide: 4287 events per 100 pt years exposure Comparator: 4579 events per 100 pt years exposure | 0 events | No safety concerns related to hypersensitivity reactions, injection-site or infusion-site reactions, development of anti-drug antibodies, neoplasms, pancreatitis, or thyroid disease were identified | Anti IL-21 + Liraglutide: 9 discontinued treatment 3 adverse events 1 protocol violation 1 pregnancy 4 withdrew from trial Anti IL-21 alone: 12 discontinued treatment 4 adverse events 1 lost to follow-up 7 withdrew from trial Liraglutide alone: 8 discontinued treatment 2 adverse events 1 death 1 protocol violation 3 withdrew from trial 2 other reasons | 11 discontinued treatment 2 adverse events 1 protocol violation 1 pregnancy 7 withdrew from trial | Intervention: 9 patients withdrew from study across intervention groups Comparator: 2 patients withdrew AEs considered possibly or probably related to study treatment appeared similar across groups |
| Greenbaum 2021 | IL6 | 12m | Intervention: 89  Comparator: 47 | 83 patients had 547 events SAEs: 3 patients with 3 events Infections: 62 patients with 168 events Bleeding events: 1 patient with 1 event Hypersensitivity: 3 patients with 3 events | 44 patients had 305 events SAEs:4 patients with 4 events Infections: 33 patients with 91 events Bleeding events: 0 Hypersensitivity: 0 | Grade 3 or higher major hypoglycaemic events Intervention: 29 patients with 73 events Comparator: 14 patients with 29 events | Significant diabetic ketoacidosis (DKA) was reported together with grade 4 hyperglycaemia | AEs leading to discontinuation of the study drug Intervention: 5 patients with 5 events Comparator: 1 patient with 1 event | 3 1 never treated 1 LTTFU  1 withdrew consent | 2 1 LTTFU  1 withdrew consent | AEs related to study treatment: 55 patients had 133 events in intervention, compared to 25 patients with 61 events in the comparator No SAEs in the tocilizumab group were considered related to study therapy. 1 in the placebo group was considered treatment related Rate of infusion reactions in both cohorts was higher in the tocilizumab compared with the placebo groups |
| Piemonti 2022 | IL8 | 12m | Intervention: 50 Comparator: 26 | 37 patients had treatment emergent AEs 3 patients had serious treatment emergent AEs (unrelated to study therapy) | 21 patients had treatment emergent AEs 1 patient had treatment emergent serious AE (unrelated to study therapy) | Intervention: 2 patients had severe hypoglycaemic events Comparator: 1 patient had severe hypoglycaemic event | NR | There were no statistically significant differences between intervention and comparator | 2 1 LTTFU 1 did not receive treatment and withdrew consent | 1 withdrew consent | Adverse drug reactions (related to study treatment): Intervention: 20 patients had 52 events Comparator: 8 patients had 17 events ADRs occurring in 10% or more of patients included dyspepsia (LDX = 16% vs. placebo = 0%) and headache (LDX = 16% vs. placebo = 15.4%). |
| Panto 1990 | Immunoglobulin | 6m | Intervention: 8 Comparator: 8 | NR | NR | NR | NR | NR | 0 | 0 | NR |
| Lorini 1991 | Immunoglobulin | 12m | Intervention: 10 Comparator: 15 | "No side effects were observed in the patients treated with IVGG" | NR | NR | NR | NR | 0 | 0 | NR |
| Colagiuri 1996 | Immunoglobulin | 24m | IVIG: 17 Transfer factor: 18 Comparator: 17 | IVIG group: 1 participant had severe side effects which required overnight hospitalisation 13 headache 7 nausea 7 lethargy  3 oedema 1 anorexia | NR | NR | NR | NR | IVIG group: 13 (details not given but reported common occurrence of side effects as a major reason for discontinuation) Transfer factor group: 8 (reasons not given) | 9 (reasons not given) | NR |
| Rother 2009 | Interferon a | 12m | 5000 units: 39 30000 units: 45 Comparator: 44 | 5000 units group: 50 events in 40 participants 30000 units group: |  |  |  | There was no difference in the occurrence of adverse events among the three treatment groups | 5000 units group:  6 withdrew consent  4 withdrew for personal reasons 2 were excluded from analysis for changing study site 30000 units group: 8 withdrew consent 5 withdrew for personal reasons  1 excluded from analysis for changing study site | 6 withdrew consent 1 withdrawn by investigator (elevated ANA) 5 withdrew for personal reasons 2 analysis for changing study site | No SAE was considered to be related to therapy. |
| Cabrera-Rode 2022 | Itolizumab | 12m | 0.4mg/kg 3 0.8mg/kg 3 1.6mg/kg 3 Comparator: 3 | No severe or serious adverse events were observed in the patients receiving Itolizumab during the follow-up period. Fever and chills associated with the first administration of the investigational product appeared in 91.7% (11/12) of the patients. The most frequent adverse reactions were: rash 75% (9), pruritus 75% (9), headache 50% (6), fever 50% (6)and chills 41.7% (5 | NR | NR | NR | N/A | 1 patient withdrew in 0.8mg/kg group | 0 | No haematological or infectious adverse events associated with the administration of Itolizumab were reported in any of the subjects involved in the study. Thus, the most adverse events were minor and did not require treatment modification. |
| Buckingham 2000 | Methotrexate | 36m | Intervention: 5 Comparator: 5 | No. participants with side effects of methotrexate 1 scattered mouth ulcers 1 upper respiratory infection (causing 1 dose to be withheld)) 1 gastroenteritis  1 severe case of varicella  1 transient increase in liver function tests | NR | NR | NR | N/A | 0 | 1 moved after 3 months | See intervention column. AEs for the comparator group were not reported |
| Hu 2013 | MSC | 24m | Intervention: 15 Comparator: 14 | NR | NR | NR | NR | N/A | 0 | 1 Immigration to other distant city | There were no obviously adverse reactions after stem cell therapy in any of the patients who completed the study protocol, and no chronic side effects or lingering effects appeared during the follow-up. |
| Carlsson 2015 | MSC | 12m | Intervention: 10 Comparator: 10 | No side effects. No tumours or chronic infections diagnosed. Neither did any of the patients report infections requiring treatment with antibiotics. Several patients had episodes of viral upper respiratory tract infections, but with similar frequency in both study groups | One patient diagnosed with microscopic colitis and Horton headache Several patients had episodes of viral upper respiratory tract infections, but with similar frequency in both study groups | None | None | N/A | 1 Due to lack of time after moving away | 1 Lack of motivation | No side effects of MSC treatment were observed |
| Izadi 2022 | MSC | 12m | Intervention: 11 Comparator: 10 | General disorders/administration site conditions: 3 patients had events Metabolism/nutrition disorders: 1 patient had an event Skin disorder: 1 patient had an event | General disorders/administration site conditions: 4 patients had events Metabolism/nutrition disorders: 6 patients had an event Skin disorder: 0 patients had an event | Intervention: GRADE 1 8 pts with 208 events GRADE 2 5 pts with 38 events GRADE 3 2 pts with 2 events GRADE 4 1 pt with 1 event Comparator: GRADE 1 9 pts with 504 events GRADE 2 9 pt with 9 events GRADE 3 3 pts with 3 events GRADE 4 2 pts with 4 events | NR | No serious adverse events in either group. | 0 | 0 | There were no major transplantation-related adverse events observed in either group. |
| Ludvigsson 2001 | Photophoresis | 36m | Intervention: 25 Comparator: 24 | 18/19 patients in had 43 episodes of common cold (11 with fever), seven of gastroenteritis, seven of tonsillitis, and four of unspecified fever. | 17/21 patients had 41 episodes of common cold (11 with fever), three of gastroenteritis, six of tonsillitis, and four of unspecified fever. One child in this group had varicella | NR | NR | On several occasions the number of white blood cells decreased somewhat during the treatment; this occurred in both the active and the placebo group | 1 technical problem,  1 afraid of the needles  1 an allergic reaction with urticaria 1 nausea 1 living too far away from the hospital 1 it was boring study | 1 changed his mind 1 afraid of the needles 1 living too far away from the hospital | One patient withdrew from the study because of nausea, and another because of urticaria. Two other children, also in the actively treated group, experienced a short period of nausea in connection with one of the treatments. Otherwise the treatment was well accepted |
| Silverstein 1988 | Pred/Azathioprine | 12m | Intervention: 23 Comparator: 23 | 3 patients stopped azathioprine therapy after 6 months; one patient had intractable nausea and vomiting, a second had frequent upper respiratory tract infections coincident with entering a day-care centre, and a third reported unexplained low-grade fever, aches and joint pains that persisted after the drug treatment was stopped. We observed no instances of bone marrow suppression, hepatotoxicity or cancer. A few patients reported hair loss during combing but no frank alopecia occurred. | NR | NR | NR | NR | 1 intractable nausea and vomiting 1 frequent upper respiratory tract infections  1 unexplained low grade fever, aches and joint pains that persisted after the drug treatment had stopped. | 3 Did not return for follow up after the initial visit. | NR |
| Secchi 1990 | Prednisolone | 36m | Pred: 10 Indomethacin: 5 Comparator: 10 | NR | NR | NR | NR | NR | Pred group: 1 Patient moved to another town Indomethacin group: 1 indomethacin treated patinet complained of a headache: withdrawal of indomethacin led to its disappearance and the patient dropped out of the study. | 0 | Only minor side effects were observed as a consequence of treatment. Two prednisone-treated patients complained of epigastralgia and facies lunaris, respectively, which did not lead to the interruption of treatment. One indomethacin-treated patient complained of headache: withdrawal of indomethacin led to its disappearance and the patient dropped out of the study. |
| Curtin 2020 | Temelimab | 6m | Intervention: 43 Comparator: 21 | The most frequent AEs were nasopharyngitis and upper respiratory tract infections, with a slightly higher frequency in the group of patients receiving temelimab during both periods. | A higher proportion of subjects in the placebo group experienced TEAEs and related TEAEs at each intensity grade compared with subjects in the temelimab group | Intervention: rate 2.09 (95% CI 1.41-3.09) Comparator: rate 2.92 (95% CI 1.66-5.13) | NR | During the double-blind period, the percentage of subjects experiencing at least one treatment emergent adverse event (TEAE) was equal between the temelimab and placebo groups | 2 withdrew consent and discontinued intervention | 1 withdrew consent and discontinued intervention | Subjects in the intervention group reported fewer TEAEs considered to be related to the study drug compared with the placebo group. |
| Quattrin 2020 | TNF | 24m | Intervention: 56 Comparator: 28 | 54 patients had events  Related to trial agent: 24 patients Serious AE: 5 patients  Any infection: 42 patients  Injection-site reaction: 13 patients | 25 patients had events  Related to trial agent: 12 patients Serious AE: 2 patients  Any infection: 19 patients Injection-site reaction: 8 patients | Any hypoglycaemic event: Intervention 37 events per patient year; Comparator 28 events per patient year | No cases of diabetic ketoacidosis reported at 12 months | N/A | 11 5 withdrew  3 LTTFU  1 withdrew to join another trial  2 went into open label extension | 5 2 LTTFU 1 study too demanding 2 non-compliance | AEs related to trial agent: Intervention: 24 patients Comparator: 12 patients |
| Gitelman 2021 | Tyrosine kinase | 24m | Intervention: 45 Comparator: 22 | 172 events in 32 patients (grade 2 or worse) Serious AEs: 4 patients with 8 events | 28 events in 13 patients (grade 2 or worse) Serious AEs: 3 patients with 3 events | Intervention: 1 event Comparator: 1 event | 1 event in the comparator group | Of the 45 participants in the imatinib group, 20 (44%) had no modification in study drug administration, 17 (38%) had temporary modifications to dosing due to adverse events (seven for neutropaenia, four for rashes, two for abnormal liver function tests, and one each for gastrointestinal issues, thrombocytopaenia, cramps, and mental health concerns), and six (13%) were permanently discontinued due to adverse events (four for liver function abnormalities, one for pre-existing cardiac arrhythmia noted after randomisation, and one for persisting allergic skin rash).  15 (68%) of 22 participants in the placebo group had no drug modification, five (23%) had transient adjustments due to adverse events | 4 2 before 12m - 1 withdrew consent, 1 died 2 before 24m withdrew consent | 1 withdrew consent before 12m | When evaluating adverse events deemed likely to be attributable to study drug, a substantially higher proportion of participants in the imatinib group had probable drug-related adverse events, with most events in the mild to moderate categories. 1 grade 5 adverse event (death) occurred in the imatinib group, which was due to an acute asthma exacerbation from a pollen storm in the region, and deemed unrelated to study drug. |
| Carlsson 2023 | MSC | 12m | Intervention: 10 Comparator: 5 | 19 events occurred | 5 events occurred | No severe hypoglycaemic events were reported | NR | 1 serious AE was reported (pregnancy) but unclear which group There was no statistically significant difference between treatment and placebo group for any category of AE. | Unclear. 1 participant dropped out due to low motivation but unclear which arm | Unclear. 1 participant dropped out due to low motivation but unclear which arm | Two participants reported AEs connected to infusion of the investigational product; one perceived the smell of corn for 72 h after infusion and one had a headache which self-resolved within 1 h of drug administration. No serious AEs related to treatment were reported |
| Bender 2024 | Tregs | 24m | Intervention: Low dose 40  High dose 24  Comparator: 46 | Low dose: 38 participants had 271 events  High dose: 24 participants had 179 events | 46 participants had 318 events | One case of hypoglycaemia in the high dose group | Low dose: 2 pts had 5 events  Comparator: 3 pts had 3 events | 21 serious AEs occurred overall | Low dose: 1 withdrew before 12m (refused labs)  4 withdrew before 24m (1 LTFU, 1 cited school, 2 refused labs)  High dose: 1 not treated, 4 reassigned to placebo, 7 reassigned to low dose  2 withdrew before 24m (1 parent work schedule, 1 refused labs) | 2 withdrew before 12m (1 LTFU, 1 withdrew consent)  5 withdrew before 24m (1 withdrew consent, 4 refused labs | 706 events (91.4%) attributed as unrelated or unlikely related to treatment; none were attributed as likely or definitely related.  Possibly related most common AEs: 6 events in  3 placebo participants (6.5%), 5 in 4 low-dose  Participants (10%), and 6 in 2 high-dose participants (17%), viral infection (2 events in 4 low-dose participants (5%), 1 in 1 high-dose participant (4%)), oropharyngeal pain (2 events in 2 placebo participants (2.2%), 3 in 1 low-dose participants (2.5%) and cough (1 event in 1 placebo participant (2.2%), 2 in 2 low-dose participants (5%), and 1 in 1 high-dose participant (4.2%)). |
| Waibel 2023 | Baricitinib | 12m | Intervention: 60  Comparator: 31 | 152 total AEs  47 (78.3%) participants with event  median AE per participant 2 [IQR 1-3]  Severe AEs: 3 participants with 5 events | 100 total AEs  26 (83.9%) participants with AE  median AE per participant 3 [IQR 1-5]  Severe AEs: 1 participant with 2 events | Severe hypoglycaemia: 1 pt with 1 event in the placebo arm | 1 pt with 2 events in the intervention arm and 1 pt with 2 events in the placebo | 7 serious AEs  22 of 60 patients in the baricitinib  group and 16 of 31 patients in the placebo group  had Covid-19 during the trial. Seventeen patients  stopped taking baricitinib and 10 patients stopped  taking placebo while they had Covid-19 | 1 discontinued intervention | 1 discontinued intervention | Seven serious adverse events recorded, none of  which were attributed to baricitinib or placebo.  The percentages of patients with infection, including upper respiratory tract infection and skin infection, were similar in the baricitinib and placebo groups. Shingles was diagnosed in 1 patient in the baricitinib group. |
| Ramos 2023 | Teplizumab | 18m | Intervention: 217  Comparator: 111 | 216 participants had events | 108 participants had events | Clinically important hypoglycaemia (mean events per patient-year)  Intervention: 4.66 (8.89)  Comparator:  4.39 (9.40) | There were no events of diabetic ketoacidosis | Covid-19 was reported  in 49 patients (22.6%) in the teplizumab  group and in 26 patients (23.4%) in the placebo  group. | 22 discontinued study:  5 AEs  11 withdrew consent  6 ‘other’  30 also withdrew from treatment but were still included in analysis | 10 discontinued study  0 AEs  8 withdrew consent  2 ‘other’  15 also withdrew from treatment but were still included in analysis | AEs leading to discontinuation of intervention: 15 participants; comparator: 3 participants  AEs leading to trial withdrawal: intervention: 12 participants; comparator: 6 participants  2 cytokine release syndrome events in the teplizumab group (both resolved within 7 days) |
| Mathieu 2024 | AG019 + teplizumab | 12m | Intervention: 15  Comparator: 3 | Adults: 10 participants reported 127 Treatment Emergent Adverse Event (TEAE)  Adolescents: 5 participants reported 26 TEAEs | Adults: 2 participants had 37 TEAEs  Adolescents: 1 participant had 2 TEAEs | NR | NR | 95.3% of TEAEs were of CTCAE grade 1 or 2. Nine TEAEs of grade 3 or higher  were reported in six AG019/teplizumab-treated participants  (none in placebo). | 1 discontinued treatment due to AE but remained in trial | None | One AG019/teplizumab-treated adult reported two grade 3 TEAEs considered reasonably related to AG019 and teplizumab (diarrhoea and vomiting) but AG019 or teplizumab treatment was not discontinued. Teplizumab treatment was discontinued in five participants due to TEAEs; all participants continued  AG019 treatment and completed the study. |
| Gaglia 2024 | Autologous dendritic cells | 12m | Intervention: 16  Comparator: 11 | Participants with any TEAE 9  Participants with any PTEAE 16 | Participants with any TEAE 6  Participants with any PTEAE 8 | Rate of  Severe hypoglycaemic events – no events in either arm | NR | No deaths, no serious adverse events, no local site reactions reported. | None | 2 withdrew consent before intervention; 1 withdrew consent during follow up | NR |
| Chujo 2023 | ATG/pegfilgrastim | 24m | Intervention: 6  Comparator: 6 | Grade 3 lymphocytopenia, which is considered to be the clinical effect of ATG, was noted in all 6 patients in the intervention arm. (No further data reported) | One severe AE, cellulitis, was reported in the Ctrl (no further data reported) | NR | NR | No grade 4 and 5 AEs were reported in either group. The frequencies of infection were not significantly different between the two groups. | NR | NR | Grade 3 lymphocytopenia, which is considered to be the clinical effect of ATG, was noted in all 6 patients in the intervention arm. |
| Tatovic 2024 | Ustekinumab | 12m | Intervention: 47  Comparator: 25 | 253 events in 41 participants  Mild: 232 events in 41 participants  Moderate: 21 events in 15 participants  Severe: 0 events | 125 events in 22 participants  Mild: 113 events in 22 participants  Moderate: 12 events in 8 participants  Severe: 0 events | No significant difference was seen in between the groups with  regard to number of clinical hypoglycaemic  events | NR | Frequency and type of side effects  were comparable between the ustekinumab and the placebo groups. | 1 withdrew, too  much commitment  1 withdrew from trial due to  health concerns | 1 withdrew, too  much commitment  1 withdrew from treatment  due to unblinding during home  dosing visit as a result of COVID | Ustekinumab was very well tolerated with no serious adverse events considered to be treatment related. |
| *NR=not reported; N/A=not applicable; AE=adverse event; f/up=follow up; LTFU=Lost to follow up; CTCAE=Common Terminology Criteria for Adverse Events; PTEAE=post-treatment adverse events* | | | | | | | | | | | |
